# Cellular assembly and functional resilience of the mammalian RNA exosome

**DOI:** 10.1038/s44318-026-00824-x

**Published:** 2026-06-25

**Authors:** Tsimafei Navalayeu, Nikolaus Beer, Monika Bebjaková, Robert W Kalis, Ulrich Hohmann, Karel Stejskal, Gabriela Krššáková, Nina Fasching, Veronika A Herzog, Niko Popitsch, Elisabeth Roitinger, Clemens Plaschka, Johannes Zuber, Stefan L Ameres

**Affiliations:** 1 Max Perutz Labs, Vienna BioCenter (VBC), Dr.-Bohr-Gasse 9, A-1030 Vienna, Austria; 2 University of Vienna, Max Perutz Labs, Dr.-Bohr-Gasse 9, A-1030 Vienna, Austria; 3 Vienna BioCenter PhD Program, a Doctoral School of the University of Vienna and the Medical University of Vienna, A-1030 Vienna, Austria; 4 Institute of Molecular Biotechnology of the Austrian Academy of Sciences (IMBA), Vienna BioCenter (VBC), A-1030 Vienna, Austria; 5 Research Institute of Molecular Pathology (IMP), Vienna BioCenter (VBC), 1030 Vienna, Austria; 6 Medical University of Vienna, Vienna BioCenter (VBC), Vienna, Austria

**Keywords:** RNA Biology, Structural Biology

## Abstract

Most eukaryotic proteins assemble into multisubunit complexes that coordinate essential cellular functions, yet the principles governing their assembly and proteostatic control remain largely undefined. Here, we systematically dissect the cellular assembly and functional organization of the RNA exosome, an essential ribonucleolytic complex, using an inducible dual-guide CRISPR/Cas9 system in mouse embryonic stem cells. We reveal a sequential assembly pathway where Exosc2, Exosc4, and Exosc7 initiate complex formation, facilitating the incorporation of barrel and cap subunits in a defined hierarchy. Unlike other structural subunits, the terminally incorporated cap subunit Exosc1 is dispensable for cell viability, revealing a modular, functionally resilient architecture. We demonstrate that orphan subunits are selectively degraded via the ubiquitin-proteasome system, enforcing stringent quality control over RNA exosome biogenesis. These findings define an assembly logic of of the mammalian exosome and uncover previously unrecognized plasticity in the composition and function of this essential ribonucleolytic complex.

## Synopsis

The RNA exosome is an essential multisubunit ribonuclease complex, but how its assembly and quality control are coordinated in cells remains poorly understood. This study shows that the mammalian RNA exosome assembles through a hierarchical pathway coupled to proteasomal surveillance yet retains functional resilience despite incomplete complex integrity.

- RNA exosome assembly is initiated by a defined nucleation module and proceeds through hierarchical incorporation of structural subunits.
- Distinct assembly intermediates are detected as discrete subcomplexes in cells.
- Unassembled subunits are selectively degraded through ubiquitin–proteasome-mediated quality control.
- Catalytic subunits associate with the exosome at distinct stages of core assembly.
- The late-assembling cap subunit Exosc1 is dispensable for mESC viability and partially dispensable for core RNA decay activity.

## Abstract (web summary)

Hierarchical assembly and proteasomal surveillance shape RNA exosome biogenesis, while late incorporation of Exosc1 reveals unexpected functional resilience.

## Graphical abstract

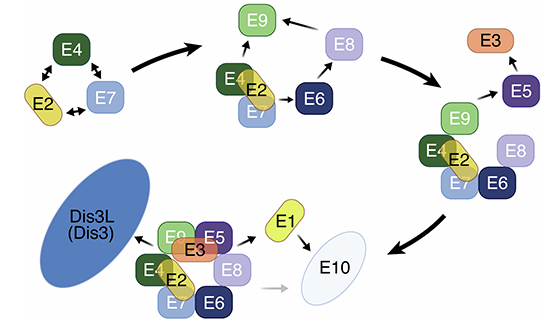

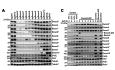

## Introduction

The biogenesis and stability of multiprotein complexes are fundamental to cellular function. Most eukaryotic proteins act within structured assemblies that coordinate essential processes such as transcription, translation and RNA metabolism (Havugimana et al, 2012; Huttlin et al, 2017; Drew et al, 2017; Brown and McMurray, 2025; Shiber et al, 2018; Aloy et al, 2004). The accurate assembly of these complexes is tightly regulated, with dedicated quality control mechanisms recognizing and eliminating orphan subunits that fail to integrate (Juszkiewicz and Hegde, 2018; Pla-Prats and Thomä, 2022). Despite the prevalence of multiprotein complexes, their assembly pathways and proteostatic dependencies remain poorly understood. While detailed studies have elucidated the stepwise biogenesis of large protein assemblies such as the ribosome and proteasome (Pla-Prats and Thomä, 2022; Murata et al, 2009; Peña et al, 2017; Bernardini et al, 2023; Morales-Polanco et al, 2022), how RNA-processing complexes form and how their stability is maintained in cells remains largely obscure.

The RNA exosome is a conserved ribonucleolytic complex that regulates the processing and degradation of most coding and non-coding RNA species across all eukaryotic domains (Chlebowski et al, 2013). In mammals, the RNA exosome consists of a catalytically inert core, comprised of a cap formed by Exosc1, Exosc2, and Exosc3, and a barrel-module composed of Exosc4, Exosc5, Exosc6, Exosc7, Exosc8, and Exosc9 (Liu et al, 2006). This RNA exosome core provides a scaffold for 3’-5’ exoribonucleases, namely Exosc10 and Dis3 in the nucleus, and Dis3L in the cytoplasm (Wasmuth et al, 2014; Tomecki et al, 2010; Staals et al, 2010). The RNA exosome is crucial for clearing products of pervasive transcription, such as promoter upstream transcripts (PROMPTs) and enhancer RNAs (eRNAs) in the nucleus, as well as for quality control and degradation of mRNA in the cytoplasm (Kalisiak et al, 2017; van Dijk et al, 2007; Kurosaki et al, 2019; Davidson et al, 2019; Gockert et al, 2022; Ogami and Suzuki, 2021). In addition to its RNA decay function, the RNA exosome contributes to the processing and maturation of various non-coding RNA species, including small nuclear RNA (snRNA), small nucleolar RNA (snoRNA) and ribosomal RNA (rRNA) (Davidson et al, 2019; Allmang et al, 1999; Tafforeau et al, 2013; Pirouz et al, 2019). Given its central role in RNA metabolism, it is essential to define how the RNA exosome assembles in a cellular context and how its integrity is monitored.

Several in vitro reconstitution approaches of the yeast and mammalian RNA exosome provided important insights into its architecture and inter-subunit interactions and indicated that pre-formed dimeric or trimeric subcomplexes may coalesce to form the RNA exosome (Liu et al, 2006; Wasmuth et al, 2014; Gerlach et al, 2018; Kowalinski et al, 2016; Kögel et al, 2024). However, whether exosome assembly follows a defined sequence in vivo or whether subunits can integrate independently remains unclear. It is also unknown whether the incorporation of all subunits is an obligatory requirement for RNA exosome function or whether partially assembled complexes can retain activity.

Genetic perturbation studies suggest that the individual contributions of RNA exosome subunits to complex function may differ. While most core components are required for cell viability in diverse model systems (Kalisiak et al, 2017; van Dijk et al, 2007; Kurosaki et al, 2019; Davidson et al, 2019; Gockert et al, 2022; Ogami and Suzuki, 2021), Exosc1 appears to be an exception. In *Trypansoma brucei*, depletion of all RNA exosome subunits except Exosc1 resulted in severe growth defects (Estévez et al, 2001). In mice, comparison of Exosc1 and Exosc2 null embryos revealed substantial phenotypic differences. Exosc2 mutants exhibit impaired uterine implantation at embryonic day 3.5 to 4 and cannot be recovered at post-implantation stages. In contrast, Exosc1 null embryos are able to progress further and form an egg-cylinder, but fail to initiate gastrulation and develop morphologically past embryonic day 5.5 (Srinivasan et al, 2024). These findings raise the possibility that Exosc1 is not strictly required for RNA exosome function and that a structurally incomplete complex may still retain activity. However, the physiological and molecular consequences of Exosc1 loss remain unresolved, and its potential role in exosome assembly and stability has not been systematically examined.

Current evidence suggests that many multiprotein complexes are assembled in a manner tightly linked to proteostatic surveillance, where orphan or misassembled subunits are selectively degraded to maintain complex integrity (Pla-Prats and Thomä, 2022; Murata et al, 2009; Peña et al, 2017; Bernardini et al, 2023; Morales-Polanco et al, 2022). Whether similar principles apply to RNA exosome biogenesis, however, remains unknown. While few proteostatic interdependencies were reported for the RNA exosome subunits (Tomecki et al, 2010; Gockert et al, 2022; Fujiwara et al, 2022; Yoshino et al, 2020; van Dijk et al, 2007; Boczonadi et al, 2014; Kammler et al, 2008; Mure et al, 2018; Belair et al, 2019), it is unclear whether their turnover is a direct consequence of failed assembly or if additional regulatory mechanisms actively govern exosome stability. Moreover, it is unknown whether RNA exosome formation requires stepwise stabilization of specific intermediates or if it follows a more flexible incorporation pathway that allows dynamic subunit exchange. Resolving these questions is critical for understanding how the RNA exosome is maintained as a functional entity under physiological and stress conditions.

Here, we systematically investigate RNA exosome assembly and proteostatic regulation in mouse embryonic stem cells (mESCs). Using inducible dual-guide CRISPR/Cas9 genome engineering alongside acute auxin-inducible degron (AID) protein depletion, we define a sequential assembly hierarchy and uncover mechanisms that regulate RNA exosome stability. We show that orphan subunits are selectively degraded via the ubiquitin-proteasome system, linking RNA exosome biogenesis to proteostatic surveillance. We further demonstrate that Exosc1, an integral member of the RNA exosome cap structure, is dispensable for RNA exosome function, revealing an unexpected resilience in exosome architecture. These findings provide a framework for understanding the cellular principles of RNA exosome assembly and expand our knowledge of how multiprotein complexes are dynamically formed, stabilized, and regulated in cells.

## Results

### A dual-guide iCas9 system probes the essential cellular function of RNA exosome subunits in mESCs

**Figure 1:**
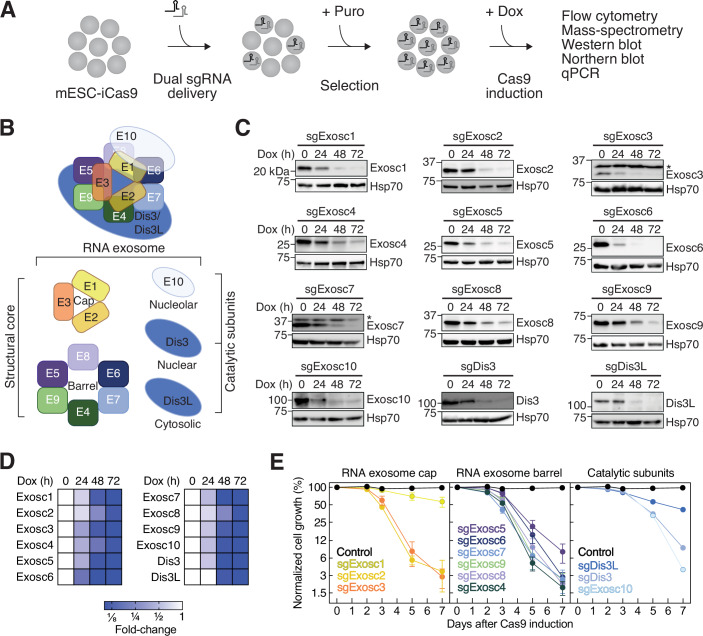
A dual-guide iCas9 system probes the essential cellular function of RNA exosome subunits in mESCs. (**A**) Outline of the experimental approach. Dual sgRNAs targeting RNA exosome subunits were delivered to iCas9 mESCs, followed by Puromycin (Puro) selection and Cas9 induction with doxycycline (Dox). (**B**) Schematic of the mammalian RNA exosome, highlighting core (cap and barrel) and catalytic subunits. “E” denotes Exosc subunits (e.g., E1 =  Exosc1). (**C**) Western blot analysis of iCas9 mESCs expressing dual sgRNAs targeting the indicated RNA exosome component and treated with Dox for 0, 24, 48, and 72 h. Hsp70 serves as a loading control. Asterisk (*) indicates a non-specific band. (**D**) Western blot quantifications from (**C**) are shown as a heatmap. Protein levels were normalized to Hsp70 and expressed relative to the 0 h time point (*n* = 2 biological replicates). (**E**) Competitive proliferation assays of inducible Exosc knockout and Olfr10 (control) mESCs relative to a parental cell line. Data were normalized to Day0 and presented as mean ± SD (*n* = 3 biological replicates). See also Fig. EV1.

**Figure EV1:**
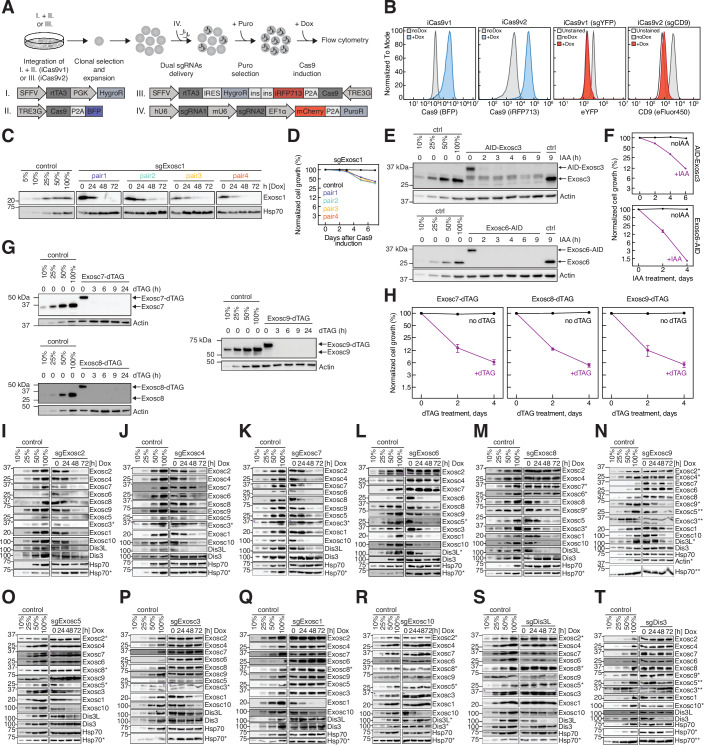
Establishment, validation, and benchmarking of a dual-guide iCas9 system to interrogate proteostatic dependencies among RNA exosome components, related to Figs. 1 and 2. (**A**) Schematic of the generation of clonal iCas9 cell lines. Roman numbers indicate vectors used for the engineering of the first (iCas9v1) and second (iCas9v2) versions of iCas9. For iCas9v1, expression cassettes for rtTA3 and Cas9 were packaged in lentiviruses and sequentially integrated into the genome. For iCas9v2, the “all-in-one” cassette was site-specifically integrated in the expression-stable locus on Chr15 (see Materials and Methods). (**B**) (Left): Flow cytometry evaluation of inducible Cas9 expression in iCas9v1 and iCas9v2 in the presence or absence of Dox. (Right): Evaluation of the editing efficiency of endogenously expressed YFP (iCas9v1) or surface marker CD9 (iCas9v2) in iCas9 cells transduced with the respective sgRNAs. For CD9 analysis, cells were immunostained before flow cytometry measurements. (**C**) iCas9 cells expressing different pairs of dual sgRNAs against Exosc1 (pair1–4) were treated with Dox for 0, 24, 48, and 72 h. Total proteins were analysed with Western blot against Exosc1 and Hsp70. Dilution series (5, 10, 25, 50, 100%) were used to estimate the sensitivity of the antibody, and Hsp70 served as a loading control. (**D**) Competitive proliferation assays of iCas9 cells harboring dual sgRNAs against Exosc1 (pair1–4) and Olfr10 (control). The percentage of sgRNA+ cells was monitored with flow cytometry. Values were normalized to Day0 and are represented as mean ± sd (*n* = 2 technical replicates). (**E**) Evaluation of the depletion efficiency of AID-tagged proteins upon IAA administration in AID-Exosc3 (top) and Exosc6-AID (bottom) cells. Tir1 (control) is a parental cell line that served as a negative control. Total proteins were analysed with Western blot using the indicated antibody. Dilution series (10, 25, 50, 100%) were used to estimate the sensitivity of the antibody, and actin represents a loading control. (**F**) Competitive proliferation assay of AID-Exosc3 (top) and Exosc6-AID (bottom) in the presence and absence of IAA. The percentage of AID-positive cells was monitored with flow cytometry. Values were normalized to Day0 and are represented as mean ± sd (*n* = 2 biological replicates). (**G**) Evaluation of the depletion efficiency of dTAG-tagged proteins upon dTAG administration in Exosc7-dTAG, Exosc8-dTAG and Exosc9-dTAG cells. Parental iCas9v2 cell line (control) served as a negative control. Total proteins were analysed with Western blot using the indicated antibody. Dilution series (10, 25, 50, and 100%) were used to estimate the sensitivity of the antibody, and actin represents a loading control. (**H**) Competitive proliferation assay of Exosc7-dTAG, Exosc8-dTAG, and Exosc9-dTAG cells in the presence and absence of dTAG. The percentage of dTAG-positive cells was monitored with flow cytometry. Values were normalized to Day0 and are represented as mean ± sd (*n* = 3 biological replicates). (**I**–**T**) iCas9 cells harboring dual sgRNAs against Exosc2 (**I**), Exosc4 (**J**), Exosc7 (**K**), Exosc6 (**L**), Exosc8 (**M**), Exosc9 (**N**), Exosc5 (**O**), Exosc3 (**P**), Exosc1 (**Q**), Exosc10 (**R**), Dis3L (**S**), and Dis3 (**T**) were treated with Dox for 0, 24, 48, and 72 h. Total proteins were analyzed with a Western blot for the indicated RNA exosome components. Dilution series (10, 25, 50, and 100%) were used to estimate the sensitivity of the antibody, and Hsp70 served as a loading control. Black asterisk (*) refers to the corresponding gel and purple asterisk (*) indicates a nonspecific band.

To systematically assess the role of individual Exosc proteins under conditions that take into account the essential role of the RNA exosome, we established a doxycycline (Dox)-inducible Cas9 system (iCas9) in mESCs (Figs. 1A and EV1A) (de Almeida et al, 2021). For gRNA design, we utilized the VBC score, which considers predicted gRNA activity, frequency of indel formation as well as amino acid composition and conservation of the targeted region to increase knockout efficiency and penetrance across a cell population (Michlits et al, 2020). To enhance protein depletion efficiency, we employed a dual gRNA targeting strategy that mitigates the potential poor editing efficiency of single gRNAs and diminishes reversions emerging from in-frame repair of single sites (Fig. 1A). We functionally validated this iCas9 system by targeting a surface marker (CD9) and yellow fluorescent protein (YFP), confirming consistent and complete protein depletion only in the presence, but not in the absence of Dox (Fig. EV1B). Upon introducing dual gRNAs targeting each one of the nine structural core components (Exosc1 to Exosc9; Fig. 1B) of the RNA exosome or its associated catalytic subunits (Exosc10, Dis3, and Dis3L; Fig. 1B), followed by Cas9 induction, we observed a progressive and consistent reduction in target protein abundance by Western blot analyses, reaching maximal depletion (i.e., ≤10%) after 72 h (Fig. 1C,D). This was consistent with previously reported RNA exosome protein half-life measurements in murine cells (Schwanhäusser et al, 2011).

To explore the physiological consequences following the loss of individual RNA exosome components, we conducted a competitive proliferation assay by mixing parental iCas9 mESCs with dual gRNA (and mCherry) expressing cells in the presence of Dox (Fig. 1E). To exclude potential target-independent adverse effects of Cas9 and/or dual-gRNA expression on cell growth, we utilized as a control a gRNA pair targeting the olfactory receptor 10 (Olfr10) gene, which is not normally expressed in mESCs. In contrast to Olfr10-targeting, which caused no discernible proliferation defects, significant growth impairment was observed upon the depletion of most structural RNA exosome core subunits, except for Exosc1. Upon Exosc1 depletion, the fraction of knockout cells merely dropped to ~50% after 7 days of Cas9 induction, in contrast to the less than 10% of gRNA-expressing cells remaining after the depletion of each other's RNA exosome core component. Targeting Exosc1 with additional pairs of gRNAs exhibited similarly weak proliferation defects, and CRISPR-based viability screens in human cancer cell lines indicate that Exosc1 knockout has a much lower impact on cell survival than other core subunits (Meyers et al, 2017; Dempster et al, 2019; Pacini et al, 2021), confirming its subordinate role in sustaining cellular proliferation (Fig. EV1C,D).

To benchmark the proliferation defects observed upon depletion of individual structural RNA exosome core subunits by iCas9 against an orthogonal acute protein depletion strategy, we introduced an auxin-inducible degron (AID) tag at the endogenous N-terminus of Exosc3 or the C-terminus of Exosc6 in Tir1-expressing mESCs (Nishimura et al, 2009), as well as dTAG-degrons at the C-termini of Exosc7, Exosc8 and Exosc9 (Nabet et al, 2018). In the absence of indole-3-acetic acid (IAA) or dTAG reagent, AID- or dTAG-tagged RNA exosome subunits showed only minor changes in protein abundance and no detectable growth defects, indicating that endogenous tagging did not perturb RNA exosome integrity (Fig. EV1E–H). Acute administration of IAA or dTAGv-1 resulted in rapid depletion of the tagged proteins (Fig. EV1E,G) and ultimately induced pronounced growth defects, closely mirroring the proliferation phenotypes observed upon inducible knockout using iCas9 (Fig. 1E).

Lastly, we performed growth competition experiments following the inducible knockout of the RNA exosome-associated catalytic subunits (Fig. 1E). Depletion of the nuclear catalytic subunits Exosc10 and Dis3 resulted in pronounced growth impairment, while the loss of the cytoplasmic catalytic subunit Dis3L led to moderate proliferation defects, in agreement with previous reports (Davidson et al, 2019; Brouze et al, 2025; Papadopoulos et al, 2022).

In summary, we established and benchmarked a dual-guide iCas9 system in mESCs to show that the depletion of individual RNA exosome subunits substantially impairs cell proliferation, while Exosc1 and Dis3L play a subordinate role.

### Selective decay of orphan exosome subunits by the ubiquitin-proteasome system reveals an ordered cellular assembly of the RNA exosome core complex

**Figure 2:**
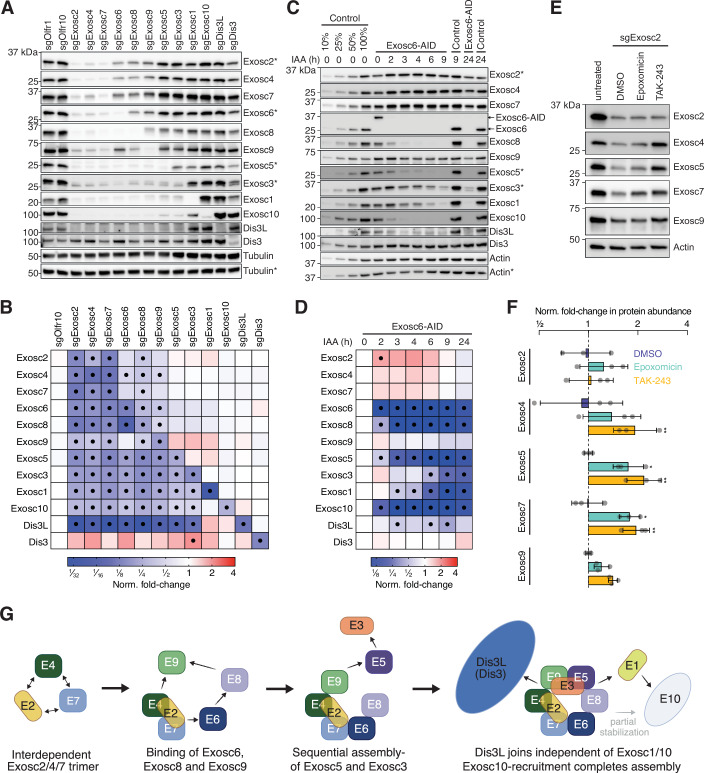
Proteolytic degradation of orphan subunits by the ubiquitin-proteasome system reveals an ordered cellular assembly of the RNA exosome. (**A**) Western blot analysis was performed on iCas9 mESCs harboring dual sgRNAs targeting the indicated RNA exosome components or Olfr1 and Olfr10 (controls), treated with Dox for 72 h. Tubulin serves as a loading control. Asterisk (*) indicate matching membranes. (**B**) Relative abundance of RNA exosome subunits was assessed 72 h after induction of sgRNAs targeting the indicated Exosc, normalized to sgOlfr1 control. Protein abundance was determined by shotgun or targeted mass-spectrometry. Values are shown relative to sgOlfr1 control sample. Statistical significance was determined using two-way ANOVA with Holm-Šídák multiple comparison correction. Black dots indicate instances where log_2_FC <−1 or log_2_FC >1 and *p*_adj_ < 0.05. (**C**) Auxin-inducible degradation of Exosc6 was analysed in Exosc6-AID mESCs treated with IAA for the indicated time in hours (h). Actin serves as a loading control. Asterisk (*) refers to matching membranes. (**D**) Western blot quantifications from (**C**) were normalized to Actin and expressed relative to untreated Exosc6-AID mESCs (*n* = 2 biological replicates). Statistical significance was determined using two-way ANOVA with Holm–Šídák multiple comparison correction. Black dots indicate instances where *p*_adj_ < 0.05. (**E**) Western blot analysis was performed on iCas9 mESCs harboring dual sgRNAs targeting Exosc2 treated with Dox for 42 h, followed by 6 h of DMSO, Epoxomicin and TAK-243 treatment. Actin serves as a loading control. (**F**) Western blot quantifications from (**E**) were normalized to Actin and expressed relative to DMSO control as mean ± sd (*n* = 3 biological replicates). Statistical significance was determined using two-way ANOVA with Holm–Šídák multiple comparison correction (*p_adj_ < 0.05, **p_adj_ < 0.01, ***p_adj_ < 0.001, ****p_adj_ < 0.0001). *P* values = 0.0187 and 0.0132 (DMSO vs Epoxomicin from top to bottom); 0.0045, 0.0032, and 0.0096 (DMSO vs TAK-243 from top to bottom). (**G**) Model of the cellular assembly of the RNA exosome. See text for details. See also Fig. EV1; Dataset EV1.

**Figure EV2:**
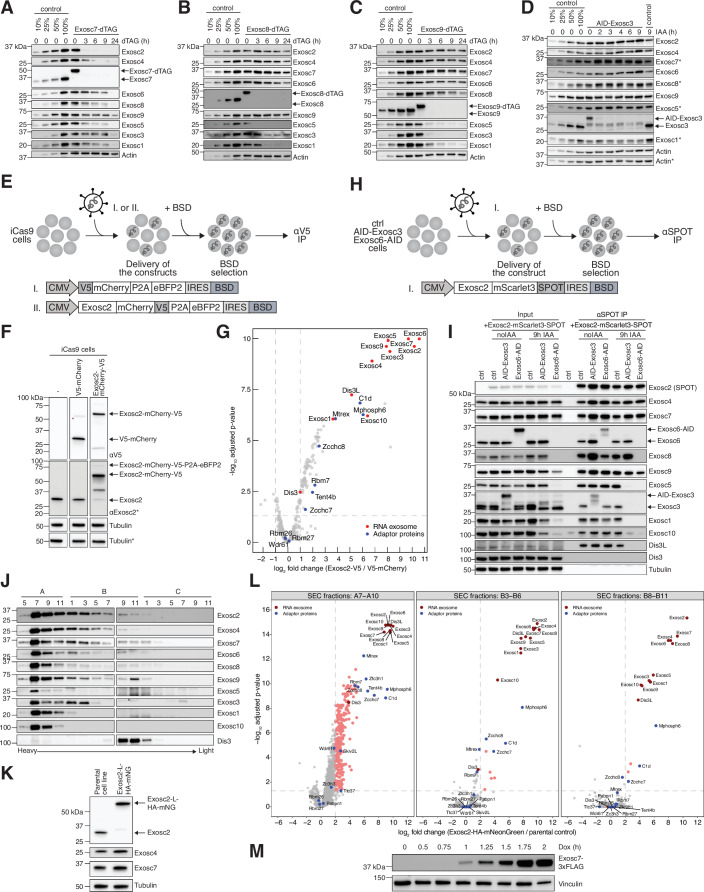
Rapid depletion of RNA exosome subunits and interrogation of RNA exosome assembly intermediates, related to Figs. 2 and 3. (**A**–**C**) Exosc7-dTAG (**A**), Exosc8-dTAG (**B**), and Exosc9-dTAG (**C**) cells were treated with dTAG for the indicated period pf time. Parental iCas9v2 cell line (control) served as a negative control. Total proteins were analyzed with Western blot using the indicated antibody. Dilution series (10, 25, 50, and 100%) were used to estimate the sensitivity of the antibody, and Actin served as a loading control. (**D**) Parental Tir1 (ctrl) and AID-Exosc3 cells were treated with IAA for the indicated period of time. Total proteins were analyzed with Western blot using the indicated antibody. Dilution series (10, 25, 50, and 100%) were used to estimate the sensitivity of the antibody, and Actin served as a loading control. Asterisk (*) refers to the corresponding gel. (**E**) Control V5-mCherry-P2A-eBFP2 or Exosc2-mCherry-V5-P2A-eBFP2 cassettes were lentivirally delivered to iCas9 cells, followed by Blasticidin S (BSD) selection. (**F**) Western blot analysis of the cell lines from (**A**) with αV5 and αExosc2 antibody. Tubulin served as a loading control. Black asterisk (*) refers to the corresponding gel. Purple asterisk (*) indicates the signal from V5-mCherry-P2A-eBFP2. (**G**) Mass-spectrometry analysis of αV5-immunoprecipitation from Exosc2-mCherry-V5 or V5-mCherry control samples. The log_2_ fold change (x axis) is plotted against the −log10 of Benjamini–Hochberg adjusted limma-moderated two-sided *t*-test *p* value (y axis; *p*_adj_). Dashed lines indicate the thresholds of *p*_adj_ < 0.05 and log_2_FC <−1 or >1 (*n* = 3 biological replicates). (**H**) Generation of ectopic expression of Exosc2-mScarlet3-SPOT in the parental Tir1 (ctrl), AID-Exosc3, and Exosc6-AID cell lines. The cassette was virally delivered, and cells were selected with Blasticidin S (BSD). (**I**) αSPOT-IP of cells from (**H**). Input and bead-bound proteins were analyzed with a Western blot using the indicated antibody. Tubulin served as a loading control. (**J**) Total protein extract of wild-type mESCs was subjected to size-exclusion chromatography followed by western blot analysis using the indicated antibodies. (**K**) Generation and validation of Exosc2-HA-mNeonGreen (labeled as Exosc2-L-HA-mNG) knockout-rescue clonal cell line. Total protein was analyzed with a Western blot using the indicated antibodies. (**L**) Total protein extract of Exosc2-HA-mNeonGreen and untagged parental control was subjected to size-exclusion chromatography, and indicated fractions were pooled, followed by αHA-immunoprecipitation and mass-spectrometry analysis. The log_2_ fold change (x axis) is plotted against the −log10 of Benjamini–Hochberg adjusted limma-moderated two-sided *t*-test *p* value (y axis; *p*_adj_). Dashed lines indicate the threshold of *p*_adj_ < 0.05 and log_2_FC >2 (*n* = 3 biological replicates). (**M**) Cells harboring doxycycline-inducible Exosc7-3xFLAG cassette were subjected to doxycycline (Dox) treatment for the indicated period of time, followed by western blot analysis. Vinculin served as a loading control.

During cellular assembly, protein complexes are frequently surveilled to detect and remove unassembled orphan subunits (Juszkiewicz and Hegde, 2018; Pla-Prats and Thomä, 2022). We systematically investigated whether the loss of individual RNA exosome subunits affects the overall integrity of the RNA exosome by monitoring reciprocal protein depletion. To this end, we targeted each structural component of the RNA exosome (i.e., Exosc1 to Exosc9) for 24, 48, and 72 h by iCas9, followed by Western blot analyses of every RNA exosome subunit (Figs. 2A and EV1I–T). To quantitatively characterize these co-destabilization patterns, we analyzed samples subjected to 72 h of inducible knockout using mass-spectrometry (Fig. 2B; Dataset EV1). In addition, we subjected endogenously tagged AID-Exosc3, Exosc6-AID, Exosc7-dTAG, Exosc8-dTAG, and Exosc9-dTAG to IAA or dTAGv-1 treatment and analyzed RNA exosome subunit abundance by Western blotting (Figs. 2C,D and EV2A–D). Using these orthogonal depletion strategies, we observed distinct and reproducible patterns of exosome protein co-destabilization that delineate ordered interdependencies among RNA exosome components and are compatible with a stepwise assembly logic in cells.

First, depletion of Exosc2, Exosc4, or Exosc7 resulted in strong co-destabilization of all RNA exosome core components (Figs. 2A,B and EV1I–K and EV2A). At the same time, these subunits remained stable in knockouts of the other RNA exosome core subunits, suggesting that Exosc2-4-7 form a stable trimeric complex that initiates RNA exosome assembly (Fig. EV1L–Q). Consistently, structural studies show extensive interaction surfaces (~2000 Å^2^ each) among these three subunits (Liu et al, 2006).

Depletion of other RNA exosome core subunits triggered selective proteostatic responses. Exosc6 depletion strongly impacted the stability of Exosc8, Exosc5, Exosc3, and Exosc1, while Exosc2, Exosc4, Exosc7 and Exosc9 were comparatively less affected (Figs. 2A,B and EV1L). Given the dependence of Exosc6 stability on Exosc2, Exosc4, and Exosc7 (Fig. EV1I–K), we conclude that Exosc6 incorporates after Exosc2/4/7 by associating with Exosc7, which forms extensive contacts (2700 Å^2^) with Exosc6 in structural analyses (Liu et al, 2006). In contrast, the dependence of Exosc9 on Exosc2/4/7 but not on Exosc6 suggests that Exosc6 and Exosc9 associate independently with the Exosc2/4/7 trimer (Figs. 2C,D and EV1I–L and EV2A).

Depletion of Exosc8 preferentially destabilized Exosc5, Exosc3, and Exosc1, while exerting a weaker effect on Exosc2, Exosc4, Exosc7, Exosc6, and Exosc9, suggesting that Exosc8 joins after the formation of the Exosc2/4/7/6 or Exosc2/4/7/6/9 subcomplexes (Figs. 2A,B and EV1M and EV2B). Consistent with this interpretation, structural analyses indicate that Exosc8 interacts with Exosc6 but does not directly contact Exosc2/4/7 (Liu et al, 2006).

Depletion of Exosc9 similarly destabilized Exosc5, Exosc3, and Exosc1 but left Exosc2, Exosc4, Exosc7, Exosc6, and Exosc8 largely unaffected (Figs. 2A,B and EV1N and EV2C). Structural data indicate that Exosc9 directly contacts Exosc4, suggesting it can associate with the Exosc2/4/7, Exosc2/4/7/6, or Exosc2/4/7/6/8 subcomplexes via Exosc4 binding that is available in all of these assemblies (Liu et al, 2006).

Targeting Exosc5 resulted in co-depletion of Exosc3 and Exosc1, indicating that Exosc5 integrates after the formation of the Exosc2/4/7/6/8/9 subcomplex, completing the ring formed by the barrel subunits of the RNA exosome (Figs. 2A,B and EV1O). Consistently, Exosc5 shares substantial interaction surfaces with Exosc9 (3800 Å^2^) and Exosc8 (2400 Å^2^) (Liu et al, 2006).

Exosc3 depletion resulted in co-depletion of Exosc1 (Figs. 2A,B and EV1P and EV2D), suggesting it incorporates after the formation of the Exosc2/4/7/6/8/9/5 subcomplex. This finding is surprising, given that structural data show minimal direct contact between Exosc3 and Exosc1 (Liu et al, 2006). Thus, Exosc3 may stabilize Exosc1 indirectly by enforcing its interaction with Exosc6 (~1400 Å^2^) and Exosc8 (~300 Å^2^), potentially by promoting a cap-binding competent conformation of the barrel through direct contacts with Exosc9 and Exosc5.

Finally, Exosc1 depletion did not affect any other core subunit (Figs. 2A,B and EV1Q), confirming its role as the final component of RNA exosome core assembly, where it directly interacts with Exosc8 and Exosc6 (Liu et al, 2006). Overall, our observations indicate that depletion of RNA exosome subunits arrests complex assembly at specific stages, while unassembled subunits undergo proteolytic degradation.

Previous studies of disease-associated RNA exosome mutations have shown that impaired assembly or destabilization of the complex can engage cellular protein quality-control pathways, including ubiquitin-proteasome system (UPS)-dependent degradation of affected components (Fasken et al, 2017; Yang et al, 2020). Building on these observations, we next investigated whether unassembled RNA exosome core subunits generated experimentally are similarly subject to regulated turnover. To do so, we depleted Exosc2 by iCas9 for 42 h, followed by proteasome inhibition with Epoxomicin (Meng et al, 1999) or inhibition of the main E1-ubiquitin ligase (Uba1) through TAK-243 for 6 h (Hyer et al, 2018). Western blot analyses revealed stabilization of orphan RNA exosome subunits Exosc4, Exosc5, Exosc7, and Exosc9, but not the genetically targeted Exosc2, upon Epoxomycin or TAK-243 treatment compared to a DMSO control (Fig. 2E,F).

In summary, our experiments demonstrate that RNA exosome integrity is actively monitored by proteostatic control mechanisms that recognize and degrade orphan core components via the ubiquitin-proteasome system. By integrating these findings with published structural studies (Liu et al, 2006), we propose a model for RNA exosome core assembly (Fig. 2G): Exosc2, Exosc4, and Exosc7 form an interdependent heterotrimer that serves as the foundation for the incorporation of Exosc6, Exosc8, and Exosc9, followed by stepwise association of Exosc5, Exosc3, and finally, Exosc1.

### Catalytic subunits associate with the RNA exosome core at distinct assembly steps

We next examined the proteostatic relationship between structural and catalytic subunits of the RNA exosome complex. Depletion of Dis3L, Dis3, or Exosc10 using iCas9 did not affect the protein levels of RNA exosome core components (Figs. 2A,B, and EV1R–T), indicating that catalytic subunits do not play a direct role in the assembly of the RNA exosome core.

By contrast, depletion of any RNA exosome core component, except for Exosc1, resulted in substantial co-depletion of the cytoplasmic catalytic subunit Dis3L (Figs. 2A–D and EV1I–Q). This behavior is consistent with structural analyses of the human RNA exosome, which show that DIS3L associates with the RNA exosome core via its PIN domain through interactions with Exosc4, Exosc7 and Exosc9 (Kögel et al, 2024). Together, these observations suggest that Dis3L associates with the cytoplasmic RNA exosome core at a late assembly stage, independently of Exosc1.

In contrast to Dis3L, the nuclear catalytic subunit Dis3 remained stable under all conditions except upon direct depletion by iCas9 (Figs. 2A–D and EV1I–T), indicating that Dis3 is largely insensitive to assembly-dependent proteostatic control.

Finally, depletion of any RNA exosome core component led to pronounced co-depletion of the nuclear catalytic subunit Exosc10 (Figs. 2A–D and EV1I,Q), indicating that Exosc10 stability strongly depends on a fully assembled RNA exosome core. Consistent with this requirement, Exosc10/Rrp6 associates with the RNA exosome core via interactions with Exosc1/Csl4, Exosc6/Mtr3, and Exosc8/Rrp43 (Weick et al, 2018; Makino et al, 2013).

Taken together, these findings show that, unlike Dis3, both Dis3L and Exosc10 are subject to orphan protein decay, which is prevented by their association with late-stage or a fully assembled RNA exosome core complexes, respectively.

### RNA exosome assembly intermediates form biochemically tractable subcomplexes

**Figure 3:**
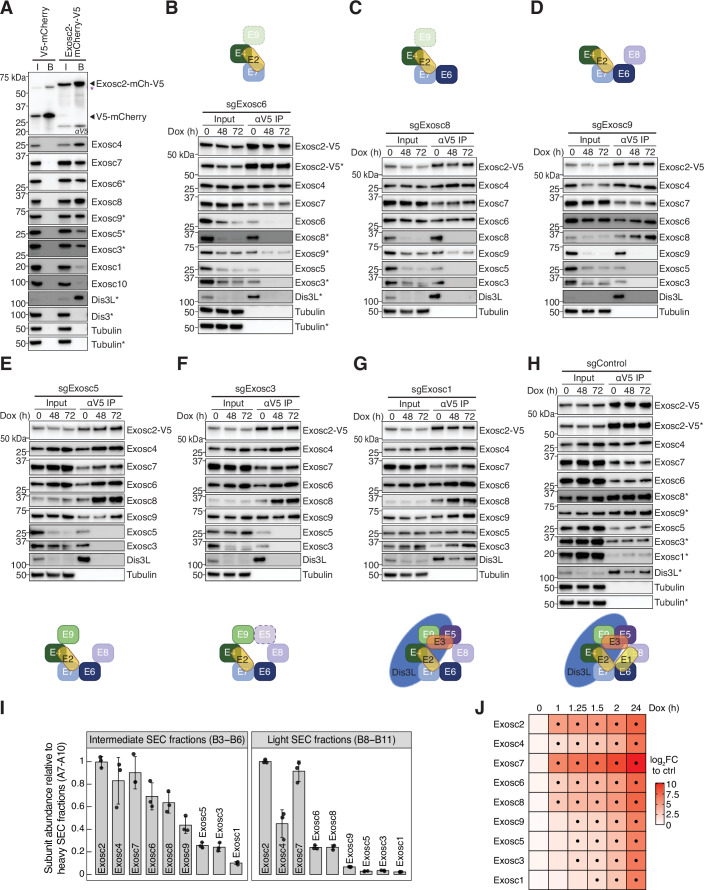
RNA exosome assembly intermediates form biochemically tractable subcomplexes and are present in wild-type cells. (**A**) V5-Immunoprecipitation (IP) was performed on the lysate of mESCs expressing V5-mCherry-P2A-eBFP2 (control) or Exosc2-mCherry-V5-P2A-eBFP2. Tubulin serves as a loading control. Black asterisk (*) refers to matching membranes. Purple asterisk (*) refers to unprocessed V5-mCherry-P2A-eBFP2. I indicates input and B indicates bead-bound proteins. (**B**–**H**) RNA exosome assembly intermediates were analysed in the lysate of mESCs expressing Exosc2-mCherry-V5-P2A-eBFP2, upon dual sgRNA-mediated depletion of Exosc6 (**B**), Exosc8 (**C**), Exosc9 (**D**), Exosc5 (**E**), Exosc3 (**F**), Exosc1 (**G**), or control Olfr10 (**H**) for the indicated time (in hours, h). αV5-IP was performed, and input and bead-bound proteins were analysed by Western blotting. Schematics depict expected RNA exosome assembly intermediates. Asterisk (*) refers to matching membranes. (**I**) HA-immunoprecipitation of Exosc2-HA-mNeonGreen from the indicated size-exclusion chromatography fractions. Protein abundance was quantified by mass-spectrometry relative to the untagged control, normalized to Exosc2 bait and presented relative to the heavy A7–A10 fractions as mean ± sd (*n* = 3 biological replicates). (**J**) FLAG-immunoprecipitation of doxycycline-inducible Exosc7-3xFLAG at the indicated time-points. Protein abundance was quantified by mass-spectrometry, and enrichment was calculated relative to the untagged control. Black dots indicate instances with log_2_FC >1.5 and *p*_adj_ < 0.05. See also Fig. EV2; Datasets EV3 and EV4.

The selective proteolytic degradation of RNA exosome components in perturbation settings suggests a model for the RNA exosome organization in cells that is consistent with the formation of biochemically tractable subcomplexes (Fig. 2G). To investigate this, we generated a cell line with ectopic expression of Exosc2-mCherry-V5 (Fig. EV2E). Notably, expression of Exosc2-mCherry-V5 led to the destabilization of endogenous Exosc2 (Fig. EV2F), a phenomenon previously observed in *Trypanosoma* brucei (Estévez et al, 2003) and consistent with orphan protein decay mechanisms described in this study. In contrast to the V5-mCherry control, immunoprecipitation of Exosc2-mCherry-V5 recovered all RNA exosome subunits, validating the effective and specific recovery of fully assembled RNA exosome complexes (Figs. 3A and EV2G; Dataset EV2). Consistent with previous reports, Dis3, unlike Dis3L, was hardly detectable upon Exosc2-V5 immunoprecipitation, perhaps reflecting the absence of assembly-dependent proteostatic control for Dis3 (Tomecki et al, 2010; Staals et al, 2010; Amorim et al, 2024; Latini et al, 2025).

We interfered with RNA exosome assembly by depleting individual core components using iCas9 for 48 or 72 h and examined complex formation by Exosc2-mCherry-V5 immunoprecipitation and Western blot analyses of the bead-bound fraction (Fig. 3B–H). Indeed, this approach allowed us to isolate compositionally distinct RNA exosome subcomplexes: Depletion of Exosc6 or Exosc8 resulted in the recovery of Exosc2/4/7 and Exosc2/4/7/6, respectively (Fig. 3B,C). Both subcomplexes also retained Exosc9, consistent with the idea that binding of Exosc9 to an immature RNA exosome core promotes resistance against orphan protein decay. In line with this assembly model, targeting Exosc9 by iCas9 also copurified the RNA exosome subcomplex Exosc2/4/7/6/8 (Fig. 3D).

Strikingly, while Exosc5 was not destabilized upon the loss of Exosc3 (Fig. EV1P), immunoprecipitation of Exosc2-mCherry-V5 from Exosc3-iKO did not co-precipitate Exosc5 (Fig. 3F), suggesting a weak or transient association of Exosc5 with the Exosc2/4/7/6/8/9 assembly intermediate. Provided that Exosc5 was copurified together with Exosc2/4/7/6/8/9/3 in Exosc1-iKO (Fig. 3G), this indicates that association of Exosc3 with the RNA exosome core is required for stable binding of Exosc5.

Finally, Exosc1 was not required for the stable association of Dis3L, consistent with the prediction that an Exosc1-deficient RNA exosome core can still form a catalytically competent complex (Fig. 3G). Targeting negative control Olfr10 did not affect RNA exosome complex formation (sgControl; Fig. 3H), and the formation of the same subcomplexes was independently corroborated following acute depletion of AID-Exosc3 and Exosc6-AID (Fig. EV2H,I). Together, these observations indicate that depletion of RNA exosome core components stalls cellular RNA exosome assembly at defined intermediate states that evade orphan protein decay by forming biochemically stable subcomplexes.

### Physiological evidence for RNA exosome assembly intermediates

To determine whether the predicted RNA exosome assemblies exist under the physiological conditions, we subjected total protein lysate from unperturbed wild-type mESCs to size-exclusion chromatography (SEC) followed by Western blot analysis (Fig. EV2J). All RNA exosome core subunits co-migrated in high-molecular-weight fractions (A7-A11), whereas progressively lighter fractions showed a selective reduction of late-stage assembly components, including Exosc5, Exosc3 and Exosc1. In the lightest fractions (B9–B11), we detected predominant co-migration of early-stage assembly components Exosc2, Exosc4, Exosc7, Exosc6, Exosc8, and Exosc9.

To overcome sensitivity limitations of Western blotting and to directly assess RNA exosome subunit associations within defined SEC fractions, we generated a knockout-rescue mES cell line expressing Exosc2-HA-mNeonGreen (Fig. EV2K). Whole-cell extracts from cells expressing Exosc2-HA were subjected to SEC, heavy (A7–A10) and lighter (B3–B6 or B8–B11) fractions were pooled, and αHA-immunoprecipitation followed by mass spectrometry was performed (Fig. EV2L; Dataset EV3). RNA exosome core subunits were significantly enriched in all analyzed fractions (log_2_FC >2, *p*_adj_ < 0.05, Fig. EV2L). In addition, RNA exosome adapter proteins, including C1d and Mphosph6, as well as components of NEXT-, PAXT-, TRAMP-, and SKI-complexes, were prominently enriched only in the heavy A7–A10 fractions, indicating RNA exosome complexes in these fractions are fully assembled and competent for adapter engagement.

To quantify subunit composition across SEC fractions, the abundance of each RNA subunit was normalized to Exosc2 bait and expressed relative to its level in the fully assembled complex (A7–A10; Fig. 3I). Whereas the intermediate B3–B6 fractions retained all RNA exosome core subunits, they showed a progressive reduction of Exosc9, Exosc5, Exosc3, and Exosc1, consistent with late-stage assembly (Fig. 2G). In contrast, the lightest B8–B11 fractions were strongly enriched for Exosc2, Exosc4, Exosc7, followed by Exosc6, and Exosc8, in agreement with early-stage assembly intermediates (Figs. 2G and 3I). Together, these data demonstrate that RNA exosome subcomplexes are detectable in unperturbed cells.

To further substantiate these findings using an independent kinetic approach, we generated a mES cell line carrying a doxycycline-inducible Exosc7-3xFLAG expression cassette integrated at a safe-harbor locus (Moussa et al, 2019). We reasoned that brief induction of Exosc7 would preferentially capture early-stage assembly associations, whereas late-stage components would accumulate only at later time points. As Exosc7-3xFLAG expression was first robustly detectable after 60 min of doxycycline treatment (Fig. EV2M), parental cells (negative control) and Exosc7-3xFLAG cells were collected after 0, 60, 75, 90, 120 min, and 24 h of induction and analyzed by FLAG immunoprecipitation followed by mass spectrometry (FLAG-IP-MS; Fig. 3J; Dataset EV4). Early-stage assembly subunits Exosc2, Exosc4, Exosc7, Exosc6, and Exosc8 were significantly enriched at 60 min (log₂FC >1.5, *p*_adj_ < 0.05), followed by accumulation of Exosc9, Exosc5, and Exosc3 at 75 min. By 90 min, all RNA exosome core subunits were significantly enriched.

Taken together, these complementary biochemical and kinetic approaches provide strong evidence that an early Exosc2–4–7–6–8 assembly subcomplex precedes the incorporation of late-stage assembly subunits Exosc9, Exosc5, Exosc3, and Exosc1 under physiological conditions.

### An immature core complex principally supports RNA exosome function and prevents apoptosis

**Figure 4:**
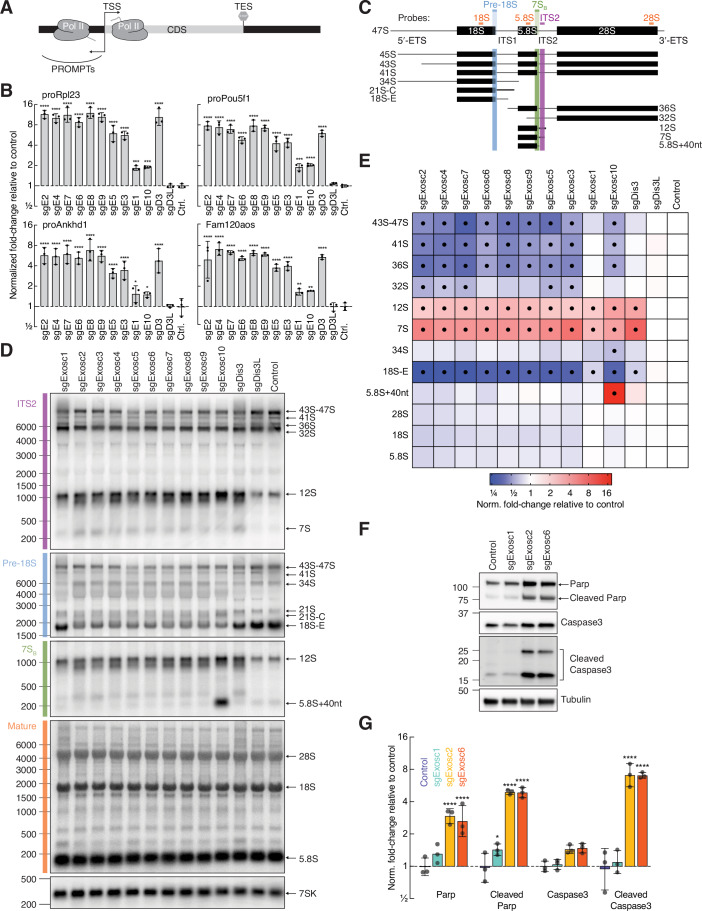
Functional assessment of RNA exosome assembly intermediates. (**A**) Schematic represents promoter upstream transcripts (PROMPTs), transcribed from the antisense strand upstream of active gene promoters. (**B**) Reverse transcription quantitative PCR (RT-qPCR) analysis of PROMPTs was performed in mESCs expressing dual sgRNAs targeting the indicated RNA exosome subunit upon treatment with Dox for 72 h. Values were normalized to Gapdh mRNA and expressed relative to sgOlfr10 control. Bargraph reports mean ± SD of three independent replicates; individual measurements are indicated. Statistical significance was determined using two-way ANOVA with Holm–Šídák multiple comparison correction. (*p_adj_ < 0.05, **p_adj_ < 0.0021, ***p_adj_ < 0.0002, ****p_adj_ < 0.0001). Exact adjusted *p* values (sgOlfr10 vs indicated condition): proRpl23: 7.4 × 10^−4^ (Exosc1), <1.0 × 10^−15^ (Exosc2), <1.0 × 10^−15^ (Exosc3), <1.0 × 10^−15^ (Exosc4), <1.0 × 10^−15^ (Exosc5), <1.0 × 10^−15^ (Exosc6), <1.0 × 10^−15^ (Exosc7), <1.0 × 10^−15^ (Exosc8), <1.0 × 10^−15^ (Exosc9), 5.4 × 10^−4^ (Exosc10), <1.0 × 10^−15^ (Dis3); proPou5f1: 4.1 × 10^−4^ (Exosc1), <1.0 × 10^−15^ (Exosc2), 7.6 × 10^−14^ (Exosc3), <1.0 × 10^−15^ (Exosc4), 1.5 × 10^−13^ (Exosc5), 5.0 × 10^−15^ (Exosc6), <1.0 × 10^−15^ (Exosc7), <1.0 × 10^−15^ (Exosc8), <1.0 × 10^−15^ (Exosc9), 9.9 × 10^−5^ (Exosc10), <1.0 × 10^−15^ (Dis3); proAnkhd1: 2.2 × 10^−2^ (Exosc1), <1.0 × 10^−15^ (Exosc2), 4.9 × 10^−11^ (Exosc3), <1.0 × 10^−15^ (Exosc4), 9.1 × 10^−10^ (Exosc5), <1.0 × 10^−15^ (Exosc6), <1.0 × 10^−15^ (Exosc7), <1.0 × 10^−15^ (Exosc8), <1.0 × 10^−15^ (Exosc9), 2.21 × 10^−2^ (Exosc10), <1.0 × 10^−15^ (Dis3); Fam120aos: 6.1 × 10^−3^ (Exosc1), 2.0 × 10^−15^ (Exosc2), 1.2 × 10^−12^ (Exosc3), <1.0 × 10^−15^ (Exosc4), 6.3 × 10^−12^ (Exosc5), <1.0 × 10^−15^ (Exosc6), <1.0 × 10^−15^ (Exosc7), <1.0 × 10^−15^ (Exosc8), <1.0 × 10^−15^ (Exosc9), 4.6 × 10⁻³ (Exosc10), <1.0 × 10^−15^ (Dis3). (**C**) A schematic of rRNA processing intermediates is shown. Individual probes are indicated. (**D**) Northern blot analysis was performed on total RNA extracted from iCas9 mESCs expressing dual sgRNAs targeting the indicated RNA exosome subunits or Olfr10 control 72 h after Dox treatment to detect the indicated rRNA processing intermediates. Probes used are shown in (**C**). (**E**) Average change in the abundance of rRNA processing intermediates as detected in (**D**), with rRNA intermediate abundance normalized to 7SK RNA and expressed relative to control (*n* = 3 biological replicates). Statistical significance was determined using two-way ANOVA with Holm–Šídák multiple comparison correction. Black dots indicate instances where *p*_adj_ < 0.05. (**F**) Western blot analysis was performed on the lysate of iCas9 mESCs expressing dual sgRNAs targeting Olfr10 (control), Exosc1, Exosc2, and Exosc6, and treated with Dox for 72 h. Tubulin serves as a loading control. (**G**) Quantification of (**F**), with protein abundance normalized to Tubulin and expressed relative to sgOlfr10 control as mean ± sd (*n* = 3 biological replicates). Statistical significance was determined using two-way ANOVA with Holm–Šídák multiple comparison correction (*p_adj_ < 0.05, **p_adj_ < 0.0021, ***p_adj_ < 0.0002, ****p_adj_ < 0.0001). Exact adjusted *p* values (sgOlfr10 vs indicated condition): PARP: 1.29 × 10⁻^6^ (Exosc2), 5.14 × 10⁻^6^ (Exosc26; Cleaved PARP: 3.13 × 10⁻² (Exosc1), 2.85 × 10⁻^10^ (Exosc2), 2.85 × 10⁻^10^ (Exosc6); Cleaved Caspase3: 1.25 × 10⁻^12^ (Exosc1), 1.25 × 10⁻^12^ (Exosc2). See also Figs. EV3–EV5; Dataset EV5.

**Figure EV3:**
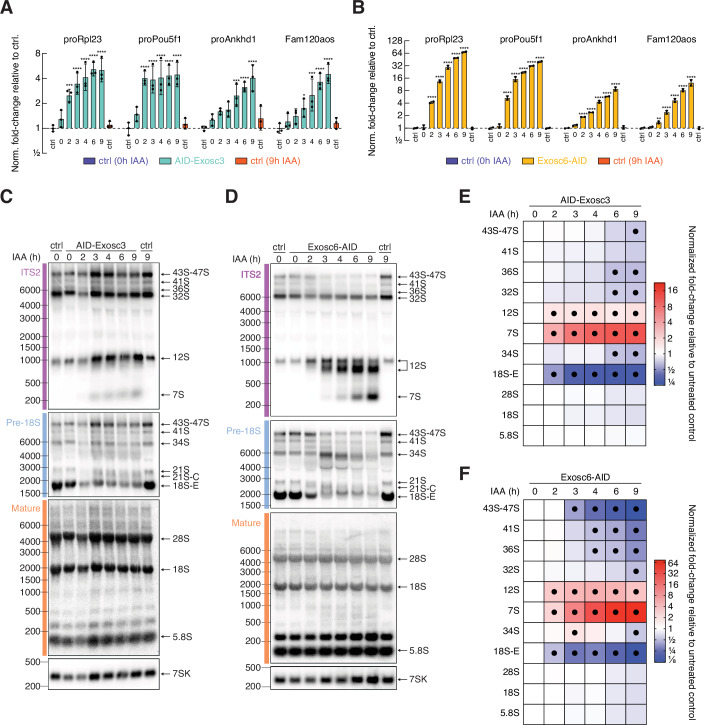
Stabilization of PROMPTs and accumulation of rRNA processing defects upon depletion of Exosc3 and Exosc6 by AID, related to Fig. 4. (**A**,** B**) Parental Tir1 (ctrl), AID-Exosc3 (**A**) and Exosc6-AID (**B**) cells were treated with IAA for the indicated time period. Total RNA was isolated, and PROMPTs abundance was measured with RT-qPCR. Values were normalized to Gapdh mRNA, and mean ± sd is shown relative to ctrl 0 h IAA (*n* = 3 biological replicates for AID-Exosc3 (**A**) and *n* = 2 biological replicates for AID-Exosc6 (**B**)). Statistical significance of the difference between a given sample and “0 h IAA ctrl” was determined with a two-way ANOVA test and Holm–Šídák multiple comparison correction (*p_adj_ < 0.05, **p_adj_ < 0.0021, ***p_adj_ < 0.0002, ****p_adj_ < 0.0001, non-significant *p*_adj_ > 0.05 is not marked). Exact adjusted *p* values: (**A**) AID-Exosc3 vs control (0 h IAA): proRpl23: 1.38 × 10⁻⁴, 5.4 × 10⁻^7^, 2.23 × 10⁻^8^, 4.34 × 10⁻^10^, 5.89 × 10⁻^10^; proPou5f1: 2.44 × 10⁻^8^, 3.92 × 10⁻^8^, 2.33 × 10⁻^8^, 9.57 × 10⁻^9^, 6.55 × 10⁻^9^; proAnkhd1: 2.53 × 10⁻⁴, 4.94 × 10⁻^6^, 4.35 × 10⁻^8^; Fam120aos: 3.38 × 10⁻^2^, 1.85 × 10⁻⁴, 2.80 × 10⁻^7^, 5.28 × 10⁻^9^; (**B**) Exosc6-AID vs control (0 h IAA): proRpl23: <1.0 × 10^−15^, <1.0 × 10^−15^, <1.0 × 10^−15^, <1.0 × 10^−15^, <1.0 × 10^−15^; proPou5f1: <1.0 × 10^−15^, <1.0 × 10^−15^, <1.0 × 10^−15^, <1.0 × 10^−15^, <1.0 × 10^−15^; proAnkhd1: 8.75 × 10^−8^, 4.42 × 10^−11^, <1.0 × 10^−15^, <1.0 × 10^−15^, <1.0 × 10^−15^; Fam120aos: 1.95 × 10^−3^, 3.64 × 10^−11^, <1.0 × 10^−15^, <1.0 × 10^−15^, <1.0 × 10^−15^. (**C**,**D**) Parental Tir1 (ctrl), AID-Exosc3 (**C**) and Exosc6-AID (**D**) cells were treated with IAA for the indicated time period. Total RNA was resolved on the agarose gel, followed by northern blot analysis. (**E**,**F**) Heatmap of northern blot quantifications for AID-Exosc3 (**E**) and Exosc6-AID (**F**). Abundance of rRNA processing intermediates was normalized to 7SK RNA, and the mean is shown relative to untreated AID-Exosc3 or Exosc6-AID samples (*n* = 3 for AID-Exosc3 and *n* = 2 for Exosc6-AID biological replicates). Statistical significance of the difference between a given sample and untreated AID-Exosc3 or Exosc6-AID controls was determined with a two-way ANOVA test and Holm–Šídák multiple comparison correction. Black dots indicate instances with *p*_adj_ < 0.05.

To investigate if RNA exosome assembly intermediates retained activity, we focused on well-established RNA exosome targets, including promoter upstream transcripts (PROMPTs; Fig. 4A), and rRNA precursors (i.e., pre-28S, pre-18S, and pre-5.8S rRNA; Fig. 4C) (Gockert et al, 2022; Ogami and Suzuki, 2021; Allmang et al, 1999; Tafforeau et al, 2013; Pirouz et al, 2019). Using RT-qPCR, we measured the RNA abundance of four prototypic PROMPTs (proRpl23, proPou5f, proAnkhd1, and Fam120aos) following depletion of RNA exosome subunits or the Olfr10 control for 72 h using iCas9 (Fig. 4B). As expected, depletion of the cytoplasmic catalytic subunit Dis3L had no effect on PROMPT levels. In contrast, the loss of RNA exosome core components, as well as the nuclear catalytic subunits Exosc10 and Dis3, led to a significant upregulation of PROMPTs (*p* < 0.05; two-way ANOVA and Holm–Šídák multiple comparison correction). Among the nuclear catalytic RNA exosome components, depletion of Exosc10 had a modest impact on PROMPT stabilization compared to Dis3, consistent with prior reports assigning a dominant role in PROMPT degradation to Dis3 (Davidson et al, 2019; Lubas et al, 2011). Notably, depletion of components that joined the RNA exosome core at late assembly stages (i.e., Exosc5, Exosc3, and particularly Exosc1) caused consistently weaker stabilization of PROMPTs compared to all other core components or Dis3 (Fig. 4B). These findings, along with the independent validation of PROMPT upregulation as early as 2 h following acute depletion of Exosc3 and Exosc6 using AID (Fig. EV3A,B), suggest that a minimal RNA exosome core structure lacking Exosc1 is partially compatible with Dis3-dependent decay of transcriptional byproducts.

To determine the impact of RNA exosome component depletion on rRNA processing, we employed Northern hybridization experiments using probes against specific rRNA biogenesis intermediates (Fig. 4C). As expected, depletion of the cytoplasmic catalytic subunit Dis3L, similar to the Olfr10 control, did not significantly impact pre-rRNA processing (*p* > 0.05; two-way ANOVA test and Holm–Šídák multiple comparison; Fig. 4D,E). However, depletion of RNA exosome core components significantly affected the processing of internal transcribed spacer (ITS) 1 (pre-18S, shown in blue, Fig. 4C–E) and ITS2 (shown in purple, Fig. 4C–E), as indicated by the downregulation of the rRNA processing intermediates 43S–47S, 41S, 36S, and 18S-E, and the upregulation of 7S and 12S (*p* < 0.05; two-way ANOVA test and Holm–Šídák multiple comparison; Fig. 4E). Furthermore, the loss of any RNA exosome core subunit led to the appearance of a non-discrete signal around the 18S-E precursor, a phenotype previously associated with the heterogenous population of 21S trimming intermediates, with 21S-C RNA representing the dominant species (Fig. 4D) (Tafforeau et al, 2013). Notably, and as observed for PROMPTs (Fig. 4B), depletion of Exosc1 resulted in a markedly weaker effect size compared to all other core subunits, suggesting that RNA exosome core structures lacking Exosc1 partially retained rRNA processing activity (Fig. 4E).

Targeting of the nuclear catalytic subunit Dis3 using iCas9 primarily upregulated 7S and 12S rRNA precursors, underscoring its selective role in ITS2 processing. We observed only a slight reduction in the abundance of the 18S-E intermediate, consistent with the notion that this processing step occurs in the nucleolus while Dis3 is mainly localized in the nucleoplasm (Fig. 4D,E) (Sloan et al, 2013).

Loss of Exosc10 resulted in a pronounced impairment of rRNA processing similar to that observed for the depletion of core subunits, but with the distinctive appearance of a longer isoform of the 5.8S precursor (Fig. 4D,E). This finding aligns with prior observations that 7S intermediate is initially trimmed by Dis3 to ~40 nucleotides from the 3´ end of mature 5.8S rRNA, followed by further processing by Exosc10 (Tafforeau et al, 2013; Henras et al, 2015; Fromm et al, 2017; Schuller et al, 2018).

To corroborate the rRNA processing defects observed upon the loss of RNA exosome core subunits, we subjected AID-Exosc3 and Exosc6-AID cell lines to an IAA treatment time-course (Fig. EV3C–F). Upregulation of 7S and 12S and downregulation of 18S-E precursors were evident after 2 h of IAA treatment, while 43S–47S, 41S, and 36S intermediates became downregulated at later time points. These observations in the AID system align with the results obtained in the iCas9 system.

The weak functional phenotype observed in PROMPT degradation and rRNA biogenesis upon Exosc1 depletion aligns with our previously noted mild effect on cell growth (Fig. 1E). To further validate these results at the cellular level, we assessed apoptosis markers by Western blot analysis following the depletion of Exosc1 for 72 h with iCas9 (Fig. 4F) (Kari et al, 2022). As expected, depletion of the early-assembled core components Exosc2 and Exosc6, but not Exosc1 or the negative control Olfr10, led to a substantial upregulation of cleaved Poly (ADP-ribose) polymerase (PARP) and cleaved Caspase3 (Fig. 4G).

In summary, we conclude that the terminal RNA exosome core component Exosc1 is partially dispensable for RNA exosome function at both the molecular and cellular levels. These findings highlight that the ordered assembly of core components establishes a functional hierarchy for the cell-essential regulation of key RNA exosome targets.

### Exosc1 is dispensable for viability in mESCs

**Figure EV4:**
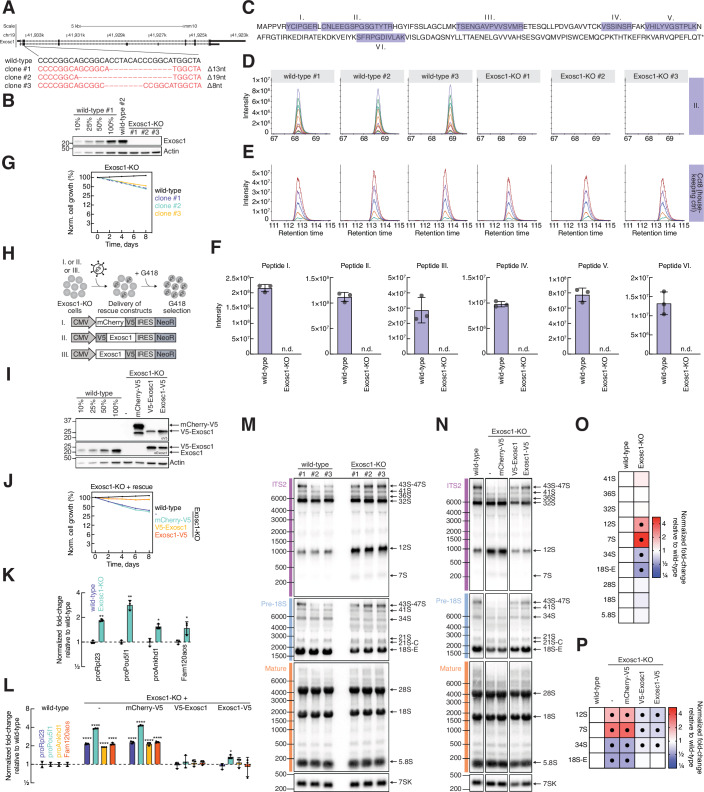
Establishment, validation and characterization of mESC lines depleted of Exosc1 by CRISPR/Cas9 genome engineering, related to Fig. 4. (**A**) Schematic of Exosc1 genomic loci. Frameshift mutations were introduced in the second coding exon with the CRISPR/Cas9 system. Genotypes of three Exosc1-KO clones used in this study are indicated. (**B**) Western blot analysis of wild-type and clonal Exosc1-KO cell lines. Dilution series (10, 25, 50, 100%) were used to estimate the sensitivity of the antibody, and actin represents a loading control. (**C**) Protein sequence of Exosc1. Six peptides that were measured with parallel reaction monitoring (PRM) mass-spectrometry are highlighted with blue rectangles and labeled with roman numbers (I-VI). (**D**) Example PRM analysis of wild-type and Exosc1-KO cells for the peptide II. PRM intensity signal (y-axis) is plotted against the retention time (x-axis). Multiple curves correspond to MS/MS = MS2 fragments. (**E**) Example PRM analysis of one of the peptides of the Cct8 protein that served as a “loading control”. Data were represented as in (**D**). See Materials and methods for information about the reference proteins used in PRM experiments. (**F**) PRM signal intensity of Exosc1 peptides in wild-type and Exosc1-KO cells shown as mean ± sd. No signal was detected in Exosc1-KO samples and is depicted as n.d. (not determined; *n* = 3 biological replicates). (**G**) Competitive proliferation assay of wild-type and Exosc1-KO cells. The percentage of wild-type or Exosc1-KO cells was monitored with flow cytometry. Values were normalized to Day0 and are shown as mean ± sd (*n* = 2 technical replicates). (**H**) Schematic of Exosc1 rescue experiments. Rescue constructs harboring mCherry-V5 or V5-Exosc1 or Exosc1-V5 were virally delivered to Exosc1-KO cells, followed by G418 selection. (**I**) Western blot analysis of the cells with rescue constructs. Membranes were probed with αV5 and αExosc1 antibody to confirm transgene expression. Dilution series (10, 25, 50, and 100%) were used to estimate the sensitivity of the antibody, and actin served as a loading control. (**J**) Competitive proliferation assay, as in (**G**), for Exosc1 rescue cells. (**K**,**L**) Total RNA from wild-type, Exosc1-KO (**K**) and rescue (**L**) experiments was isolated, and PROMPTs abundance was measured with RT-qPCR. Values were normalized to Gapdh mRNA, and mean ± sd is shown relative to wild-type (*n* = 3 biological replicates). Statistical significance of the difference between a given sample and wild-type was determined with an unpaired *t*-test (for **K**) or two-way ANOVA (for **L**) and Holm-Šídák multiple comparison correction. (*n* = 3 biological replicates, (*p_adj_ < 0.05, **p_adj_ < 0.0021, ***p_adj_ < 0.0002, ****p_adj_ < 0.0001, non-significant *p*_adj_ > 0.05 is not marked). Exact adjusted *p* values: (**K**) WT vs Exosc1-KO: 2.0 × 10⁻⁴, 7.0 × 10⁻⁴, 7.9 × 10⁻³, 2.2 × 10⁻²; (**L**) WT vs Exosc1-KO: 3.38 × 10⁻^7^, 4.42 × 10⁻^13^, 8.73 × 10⁻^6^, 3.73 × 10⁻^7^; WT vs Exosc1-KO + mCherry-V5: 1.14 × 10⁻^7^, 5.8 × 10⁻^14^, 6.05 × 10⁻^7^, 9.57 × 10⁻^8^; WT vs Exosc1-KO + Exosc1-V5: 4.34 × 10⁻². (**M**,** N**) Total RNA from wild-type, Exosc1-KO (**M**) and rescue (**N**) experiments was resolved on the agarose gel, followed by northern blot analysis. (**O**,**P**) Heatmap of northern blot quantifications for wild-type, Exosc1-KO (**O**) and rescue (**P**) experiments. Values were normalized to 7SK RNA, and the mean is presented relative to wild-type (*n* = 3 biological replicates). Statistical significance of the difference between a given sample and wild-type was determined with an unpaired *t*-test (for **O**) or two-way ANOVA (for **P**) and Holm–Šídák multiple comparison correction (*n* = 3 biological replicates. Black dots indicate instances with *p*_adj_ < 0.05.

To determine whether mouse embryonic stem cells (mESCs) can tolerate a complete loss of Exosc1 protein, we generated three independent clonal knockout mES cell lines with frameshift mutations in the second exon of the Exosc1 gene using CRISPR/Cas9 genome engineering (Exosc1-KO; Fig. EV4A). The absence of Exosc1 protein was confirmed by Western blot analysis (Fig. EV4B) and targeted parallel reaction monitoring (PRM) mass spectrometry (Fig. EV4C–F). A competitive proliferation assay revealed moderate growth defects in clonal Exosc1-KO compared to wild-type (WT) mES cells (Fig. EV4G), similar to our observations with the iCas9 system (Fig. 1E). To rule out off-target effects, we complemented Exosc1-KO cells with ectopic expression of N- or C-terminally V5-tagged Exosc1 (Fig. EV4H,I). Both V5-tagged Exosc1 constructs, but not the control mCherry-V5, rescued the proliferation defects (Fig. EV4J).

Functional characterization of the RNA exosome in Exosc1-KO clones showed moderate upregulation of PROMPTs (Fig. EV4K), which was fully rescued by re-expression of V5-tagged Exosc1 (Fig. EV4L). Analysis of rRNA processing in Exosc1-KO clones revealed a mild downregulation of the 18S-E precursor and weak upregulation of 7S and 12S precursors, consistent with results from inducible knockout experiments (Fig. EV4M,O). Re-expression of V5-tagged Exosc1 successfully restored normal rRNA processing (Fig. EV4N,P). The rRNA processing defects were more pronounced in the Exosc1-KO clones compared to Exosc1 depletion with iCas9 (Fig. 4), likely due to terminal depletion effects in the clonal Exosc1 knockout.

**Figure EV5:**
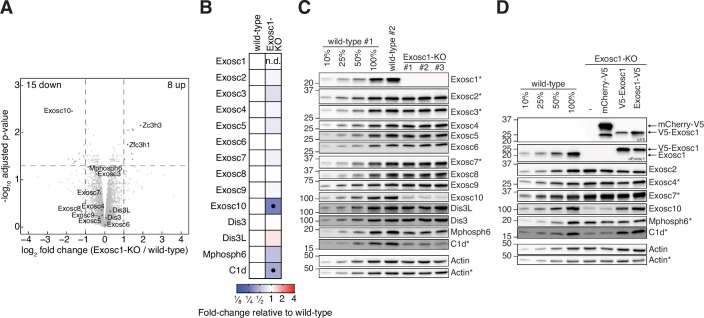
Proteomics characterization of clonal Exosc1-KO mESCs, related to Fig. 4. (**A**) Volcano plots displaying whole proteome changes for Exosc1-KO. The log_2_ fold change (x axis) is plotted against the −log10 of Benjamini–Hochberg adjusted limma-moderated two-sided *t*-test *p*-value (y axis; *p*_adj_). Dashed lines indicate the thresholds of *p*_adj_ < 0.05 and log_2_FC <−1 or >1 (*n* = 3 biological replicates). (**B**) Relative protein abundance of RNA exosome subunits in Exosc1-KO determined by shotgun and PRM mass-spectrometry. Values are shown relative to wild-type sample. Statistical significance of the difference between Exosc1-KO and wild-type was determined with an unpaired *t*-test and Holm–Šídák multiple comparison correction (*n* = 3 biological replicates). Black dots indicate instances where *p*_adj_ < 0.05. No signal for Exosc1 peptides was measured in Exosc1-KO samples and is depicted as n.d. (not determined). (**C**, **D**) Western blot analysis of wild-type, Exosc1-KO (**C**) and rescue cells (**D**) with the indicated antibody. Dilution series (10, 25, 50, and 100%) were used to estimate the sensitivity of the antibody and Actin represents a loading control. Asterisk (*) refers to the corresponding gel.

Further analysis of global proteome alterations in Exosc1-KO mES clones using shotgun mass spectrometry revealed that among 5877 detected proteins, only 15 were significantly down- and eight upregulated (*p* < 0.05; limma and Benjamini–Hochberg multiple comparison correction; Fig. EV5A; Dataset EV5). GO-term enrichment analyses for these differentially expressed proteins did not produce significant results. Reassuringly, Exosc10 stood out among the downregulated proteins (Fig. EV5A,B), consistent with our observations using the iCas9 system (Fig. 2), and confirmed by Western blot analyses (Fig. EV5C,D).

We concluded that long-term and complete depletion of Exosc1 has a minor impact on RNA exosome function, mES cell growth and global proteostasis. Hence, Exosc1 is dispensable for viability in mESCs.

## Discussion

Multiprotein complexes are ubiquitous, yet systematically understanding the role of individual subunits in their function, assembly and stability remains challenging, primarily due to technical limitations in protein depletion technologies (Havugimana et al, 2012; Huttlin et al, 2017; Drew et al, 2017). We addressed these challenges by combining the scalability and ease of parallel gRNA-based gene targeting with doxycycline-inducible Cas9 (iCas9) expression, which accounts for indirect effects and the essential role of many protein complexes (de Almeida et al, 2021). For gRNA design, we utilized the VBC score, which integrates predicted gRNA activity, indel formation frequency, as well as amino acid composition and conservation of the targeted region to increase knockout efficiency (Michlits et al, 2020). By leveraging dual gRNA targeting, we further enhanced knockout penetrance within cell populations, mitigating the potential poor editing efficiency and in-frame repair of single sites. We benchmarked this approach against time- and labor-intense acute protein depletion strategies (i.e., auxin-inducible degron), demonstrating that the dual-gRNA iCas9 systems provide a robust and scalable method to systematically investigate direct proteostatic dependencies (Figs. 1 and EV1).

By applying the dual-gRNA iCas9 system in mESCs to each of the twelve components of the RNA exosome (Fig. 1C), we confirmed the essential cellular function of most RNA exosome subunits and observed specific patterns of exosome protein co-destabilization via the ubiquitin proteasome system (Figs. 1D and 2A–F). These findings unveil the elaborate proteostatic control mechanisms governing an essential molecular machine that directs RNA processing and turnover of most coding and non-coding RNA species in eukaryotes (Chlebowski et al, 2013). In fact, most RNA exosome components, including all core components (i.e., Exosc1 to Exosc9), as well as the cytoplasmic Dis3L and the nuclear Exosc10, are subject to orphan protein decay (Figs. 2A,B and EV1I–S). These observations align with and significantly extend previous studies reporting selective orphan protein decay of RNA exosome components in *Trypanosoma brucei*, and human cells, suggesting that proteostatic control of the RNA exosome complex is evolutionarily conserved (Tomecki et al, 2010; van Dijk et al, 2007; Gockert et al, 2022; Fujiwara et al, 2022; Yoshino et al, 2020; Boczonadi et al, 2014; Kammler et al, 2008; Mure et al, 2018; Belair et al, 2019; Fasken et al, 2017; Yang et al, 2020; Estévez et al, 2003).

Interestingly, despite their structural similarity, the nuclear Dis3, unlike its cytoplasmic paralog Dis3L, does not rely on association with the RNA exosome core for protein stability (Figs. 2 and EV1I–Q). Both proteins depend on the PIN domain for RNA exosome association, yet Dis3 appears to escape proteostatic control that targets the cytoplasmic Dis3L (Tomecki et al, 2010; Staals et al, 2010). Perhaps, Dis3 is specifically shielded prior to nuclear import or lacks destabilizing sequence features present in Dis3L. Alternatively, Dis3 may function independently of the RNA exosome core, as suggested by in vitro and in vivo studies (Liu et al, 2006; Latini et al, 2025; Dziembowski et al, 2007; Lorentzen et al, 2008). However, our findings indicate that both Dis3 and the RNA exosome core are crucial for clearing PROMPTs or processing of 12S and 7S rRNA precursors, arguing against an independent role of Dis3 at least in these contexts (Fig. 4B,D,E).

Despite detailed information on RNA exosome architecture and inter-subunit interactions emerging from biochemical and structural analyses of reconstituted yeast and human complexes, how the complex assembles in cells has remained unclear (Liu et al, 2006; Wasmuth et al, 2014; Gerlach et al, 2018; Weick et al, 2018; Kowalinski et al, 2016; Makino et al, 2013; Schuller et al, 2018; Liu et al, 2016; Wasmuth et al, 2017; Kögel et al, 2024). Based on the selective proteolytic degradation of orphan RNA exosome components observed in our study (Figs. 2A,B and EV1I–T), we propose a model for RNA exosome assembly that substantially differs from previous in vitro models invoking the association of pre-formed barrel dimers bridged by cap components (Liu et al, 2006). Instead, our data suggest that RNA exosome assembly in cells is initiated by the formation of an interdependent heterotrimer consisting of the cap component Exosc2 and the barrel subunits Exosc4 and Exosc7. Formation of this core module enables subsequent association of Exosc6, Exosc8, Exosc9, and Exosc5 to complete barrel assembly, followed by recruitment of Exosc3 and, finally, Exosc1 to finalize the cap structure (Fig. 2G).

We note that variation may occur at early assembly stages, with multiple non-mutually exclusive assembly subcomplexes, including Exosc2/4/7/9, Exosc2/4/7/6/9, and Exosc2/4/7/6/8. Interestingly, native mass-spectrometry analysis of the yeast RNA exosome identified the presence of analogous Exosc2/4/7/9 and Exosc2/4/7/6/9 subcomplexes, albeit in association with Dis3 (Olinares and Chait, 2020); whether these represent bona fide assembly intermediates in yeast cells remains to be determined. Notably, our size-exclusion analysis of unperturbed wild-type mESCs suggests additional heterogeneity at the earliest assembly stages. In the lightest SEC fractions (B8–B11), Exosc2 and Exosc7 showed particularly strong enrichment, whereas Exosc4 was less abundant (Figs. 3I and EV2J,L). This pattern raises the possibility that mammalian RNA exosome assembly may initiate through smaller, transient species—such as Exosc2/7 dimers—prior to formation of the Exosc2/4/7 core trimer. However, given the limited resolution of the SEC, definitive assignment of such minimal assemblies is not possible. Importantly, irrespective of potential early heterogeneity, our biochemical fractionation, kinetic incorporation assays, and proteostatic dependency mapping converge on a defined series of stable assembly intermediates. These intermediates are captured along a coherent assembly trajectory that is fully consistent with the ordered proteostatic dependencies observed upon targeted subunit depletion, supporting a stepwise and regulated cellular pathway for mammalian RNA exosome assembly.

While our model is largely consistent with known structural interaction data, it suggests one notable deviation during cellular RNA exosome assembly. Specifically, we propose that a structural reconfiguration may occur after Exosc3 incorporation into the Exosc2/4/7/6/8/9/5 complex but prior to Exosc1 recruitment (Figs. 2A,B and 3F,G). In the human RNA exosome structure, EXOSC3 engages extensively with EXOSC9 and EXOSC5 but makes only limited direct contact with EXOSC1. This raises the possibility that Exosc3 incorporation promotes a cap-binding-competent conformation of the exosome barrel that facilitates subsequent Exosc1 association with Exosc6 and Exosc8 (Liu et al, 2006; Weick et al, 2018). While the precise molecular nature of this rearrangement remains unresolved, it may involve exposed interaction interfaces, chaperon-assisted subunit incorporation, or context-dependent masking of destabilizing motifs that are relieved only at specific assembly states. Alternatively, it is also possible that the limited direct contacts between Exosc3 and Exosc1 observed in the mature structure are already sufficient to stabilize Exosc1 binding once the appropriate assembly context is achieved.

The orphan protein decay of Exosc10 and Dis3L implies that catalytic subunit recruitment occurs at a late stage of RNA exosome assembly via distinct core assemblies (Figs. 2 and 3). Notably, Dis3 and Dis3L bind to the RNA exosome core through interactions with Exosc4, Exosc7 and Exosc9, which we predict form a complex before Exosc5, Exosc3, and Exosc1 recruitment (Weick et al, 2018; Kögel et al, 2024; Makino et al, 2013; Bonneau et al, 2009). Given that Dis3L protein stability depends on Exosc5 and Exosc3, but not Exosc1, stable Dis3L binding to the RNA exosome core must be masked prior to the recruitment of Exosc3 (Fig. 2A,B). Based on their similar domain architecture, Dis3 may integrate into the RNA exosome core at a similar stage as Dis3L (Tomecki et al, 2010; Staals et al, 2010). In contrast, Exosc10 stability depends on all RNA exosome core components, including Exosc1, indicating that its stable association relies on a completely assembled RNA exosome cap structure (Fig. 2A,B). Overall, the presented assembly model provides a testable framework for future structural studies of experimentally tractable RNA exosome assembly intermediates (Fig. 3).

The ordered assembly of the RNA exosome core complex establishes a functional hierarchy among its subunits (Fig. 4). Early-assembled exosome components (i.e., Exosc2, Exosc4, Exosc7, Exosc6, Exosc8, and Exosc9) are equally essential for PROMPT degradation, rRNA processing, and cell survival in mESCs. In contrast, depleting structural components that associate with the RNA exosome at late stages results in milder phenotypic consequences. For example, Exosc5 or Exosc3 depletion caused consistently weaker PROMPT stabilization compared to other core components, perhaps because Dis3 retains the ability to associate with Exosc4, Exosc7 and Exosc9 in a core subcomplex that lacks Exosc5, Exosc3 and Exosc1 (Weick et al, 2018; Makino et al, 2013; Bonneau et al, 2009). However, both Exosc5 and Exosc3 are essential for rRNA processing, which largely depends on Exosc10, recruited to the RNA exosome core via Exosc6 and Exosc8, beyond Exosc1 (Weick et al, 2018; Makino et al, 2013). Strikingly, Exosc1, the final component in RNA exosome core assembly, is largely dispensable for PROMPT turnover, rRNA processing, and mESC growth. This suggests that partial, low-affinity interactions of Dis3 and Exosc10 with the immature RNA exosome core may be sufficient to prevent apoptosis and sustain mESC viability, even upon complete, long-term depletion of Exosc1. In the absence of Exosc1, the RNA exosome may retain activity through interactions between the immature core and cofactors that provide substrate specificity, such as Mtr4 (Mtrex/Skiv2l2), C1d and Mphosph6, the latter two of which are only partially destabilized when Exosc1 is absent (Fig. EV5A–D)(Gerlach et al, 2018; Weick et al, 2018; Schuch et al, 2014).

In vivo, the observed functional hierarchy among RNA exosome core components could explain the differential developmental roles of RNA exosome subunits in mouse embryos (Srinivasan et al, 2024). Exosc2 knockouts result in impaired uterine implantation at embryonic day (E) 3.5–4 and cannot be recovered at post-implantation stages, while Exosc1 knockouts progress further, forming an egg-cylinder but failing to initiate gastrulation or to develop beyond E5.5. Thus, while complete RNA exosome core assembly, including Exosc1, is dispensable for mESC viability and early embryonic development, it appears crucial for establishing multiple cell layers and coordinating body plan formation during gastrulation.

Overall, our findings establish a compelling framework for future investigations into the molecular basis, biological significance and biomedical relevance of proteostatic mechanisms that govern RNA exosome assembly, homeostasis, and function (Zaki et al, 2023; Burns et al, 2018; Saramago et al, 2019; Boczonadi et al, 2014; Di Donato et al, 2016; Wan et al, 2012; Morton et al, 2018; Slavotinek et al, 2020; Somashekar et al, 2021; Damseh et al, 2023; Fasken et al, 2024).

## Methods

**Table:** Reagents and tools table

Reagent/resource	Reference or source	Identifier or catalog number
**Experimental models**		
mESCs AN3-12	(Elling et al, 2017)	N/A
mESCs: chr15 safe-harbor	(Moussa et al, 2019)	N/A
Lenti-X 293 T	Clontech	Cat#632180
mESCs: Exosc1-KO #1	This study	N/A
mESCs: Exosc1-KO #2	This study	N/A
mESCs: Exosc1-KO #3	This study	N/A
mESCs: Exosc1-KO mCherry-V5	This study	N/A
mESCs: Exosc1-KO V5-Exosc1	This study	N/A
mESCs: Exosc1-KO Exosc1-V5	This study	N/A
mESCs: OsTir1-3xMyc-T2A-eYFP	This study	N/A
mESCs: OsTir1-3xMyc-T2A-eYFP AID-Exosc3	This study	N/A
mESCs: OsTir1-3xMyc-T2A-eYFP Exosc6-L-AID-V5	This study	N/A
mESCs: OsTir1-3xMyc-T2A-eYFP Exosc2-mScarlet3-SPOT	This study	N/A
mESCs: OsTir1-3xMyc-T2A-eYFP AID-Exosc3 Exosc2-mScarlet3-SPOT	This study	N/A
mESCs: OsTir1-3xMyc-T2A-eYFP Exosc6-L-AID-V5 Exosc2-mScarlet3-SPOT	This study	N/A
mESCs: iCas9v1 (rtTA3-Hygro, TRE3G-Cas9-eBFP2)	This study	N/A
mESCs: iCas9v2 (rtTA3-Hygro, TRE3G-Cas9-iRFP713)	This study	N/A
mESCs: iCas9v2 V5-mCherry-P2A-eBFP2	This study	N/A
mESCs: iCas9v2 Exosc2-mCherry-V5-P2A-eBFP2	This study	N/A
mESCs: iCas9v2 Exosc2-HA-mNeonGreen knockout-rescue	This study	N/A
mESCs: iCas9v2 Exosc7-L-dTAG-V5	This study	N/A
mESCs: iCas9v2 Exosc8-L-dTAG-V5	This study	N/A
mESCs: iCas9v2 Exosc9-L-dTAG-V5	This study	N/A
mESCs: TRE3G-Exosc7-L-3xFLAG-P2A-mCherry	This study	N/A
**Recombinant DNA**		
pLenti-CRISPR-v2-GFP	(Herzog et al, 2017)	Internal ID: pTN080
pLenti-CRISPR-v2-mCherry	This study	Internal ID: pTN089
pYR03-FRT-SFFV-OsTir1-3xMyc-T2A-eYFP-F3	This study	Internal ID: 560
pCAG-Flp-GFP	(Matsuda and Cepko, 2007)	Internal ID: pTN095
pCMV_NLS-Cre-HSVTKpA	(Leeb et al, 2010)	Internal ID: pTN202
pCR4-TOPO	Invitrogen	Cat#K457502
pCR4-TOPO-AID-Exosc3-HT	This study	Internal ID: 550
pCR4-TOPO-Exosc6-L-AID-V5-HT	This study	Internal ID: pTN118
pDonor-Exosc7-GGGS3-dTag-lox5171-IRES-mScarlet3-lox5171-HT	This study	Internal ID: pTN066
pDonor-Exosc7-GGGS3-dTag-lox5171-IRES-mNeonGreen-lox5171-HT	This study	Internal ID: pTN067
pDonor-Exosc8-GGGS3-dTag-lox5171-IRES-mScarlet3-lox5171-HT	This study	Internal ID: pTN075
pDonor-Exosc8-GGGS3-dTag-lox5171-IRES-mNeonGreen-lox5171-HT	This study	Internal ID: pTN076
pDonor-Exosc9-GGGS3-dTag-lox5171-IRES-mScarlet3-lox5171-HT	This study	Internal ID: pTN077
pDonor-Exosc9-GGGS3-dTag-lox5171-IRES-mNeonGreen-lox5171-HT	This study	Internal ID: pTN078
pX330-CMV-Cas9-hGem	(Gutschner et al, 2016)	Addgene #71707; RRID:Addgene_71707
pX330-CMV-Cas9-hGem-P2A-mCherry	This study	Internal ID: pTN071
pX330-CMV-Cas9-hGem-P2A-mTagBFP2	This study	Internal ID: pTN072
pCMVR8.74	Didier Trono lab	Addgene #22036; RRID:Addgene_22036
pMD2.G	Didier Trono lab	Addgene #12259; RRID:Addgene_12259
pRRL-SFFV-rtTA3-IRES-EcoRec-PGK-Hygro	(Michlits et al, 2020)	Internal ID: pTN091
pRRL-TRE3G-Cas9-P2A-eBFP2	(Michlits et al, 2020)	Internal ID: pTN090
pYR03-FRT-Cbh-rTetR-IH-ins2-iRFP713-P2A-Cas9-TRE3G-ins2-F3	This study	Internal ID: pTN004
pDual-hU6-sgRNA-mU6-sgRNA-EF1α-mCherry-P2A-PuroR	(Pechincha et al, 2022)	Internal ID: pTN108
pRRL	(Rebendenne et al, 2021)	Addgene #145840; RRID:Addgene_145840
pRRL-CMV-mCherry-L-V5-IRES-NeoR	This study	Internal ID: pTN135
pRRL-CMV-V5-L-Exosc1-IRES-NeoR	This study	Internal ID: pTN137
pRRL-CMV-Exosc1-L-V5-IRES-NeoR	This study	Internal ID: pTN138
pRRL-CMV-V5-mCherry-P2A-BFP-IRES-BSD	This study	Internal ID: pTN136
pRRL-CMV-Exosc2-L-mCherry-V5-P2A-BFP-IRES-BSD	This study	Internal ID: pTN148
pRRL-CMV-Exosc2-L-mScarlet3-SPOT-IRES-BSD	This study	Internal ID: pTN189
pRRL-CMV-Exosc2-L-HA-mNeonGreen-IB_gRNA_resistant	This study	Internal ID: pTN144
pYR03-FRT-Cbh-rTetR-IH-ins2-imCherry-P2A-3xFLAG-3xGGGS-Exosc7-TRE3G-ins2-F3	This study	Internal ID: pTN234
**Antibodies**		
Rabbit polyclonal anti-Exosc1	Bethyl	Cat#A303-885A-T; RRID:AB_2620235
Rabbit polyclonal anti-Exosc2	Bethyl	Cat#A303-886A; RRID:AB_2620236
Rabbit polyclonal anti-Exosc2	Proteintech	Cat#14805-1-AP; RRID:AB_2101837
Mouse monoclonal anti-Exosc2	Proteintech	Cat#66099-1-Ig; RRID:AB_2881498
Rabbit polyclonal anti-Exosc3	Novus	Cat#NBP2-22261; RRID:AB_2750849
Rabbit polyclonal anti-Exosc3	Proteintech	Cat#15062-1-AP; RRID:AB_2278183
Rabbit polyclonal anti-Exosc4	Proteintech	Cat#15937-1-AP, RRID:AB_2293806
Mouse monoclonal anti-Exosc4 (G-9)	Santa Cruz	Cat#sc-166772, RRID:AB_2278190
Rabbit polyclonal anti-Exosc5	Proteintech	Cat#15627-1-AP, RRID:AB_2293809
Rabbit polyclonal anti-Exosc6	Thermo Fisher Scientific	Cat#PA5-49138, RRID:AB_2634594
Mouse monoclonal anti-Exosc6 (D-10)	Santa Cruz	Cat#sc-398277
Mouse monoclonal anti-Exosc7 (A-1)	Santa Cruz	Cat#sc-393686
Rabbit polyclonal anti-Exosc7	Proteintech	Cat#25292-1-AP, RRID:AB_2880011
Rabbit polyclonal anti-Exosc8	Proteintech	Cat#11979-1-AP, RRID:AB_2101981
Rabbit polyclonal anti-Exosc9	Proteintech	Cat#24470-1-AP, RRID:AB_2879559
Mouse monoclonal anti-Exosc9	Proteintech	Cat#67636-1-Ig, RRID:AB_2882837
Mouse monoclonal anti-Exosc9 (D-6)	Santa Cruz	Cat#sc-271815, RRID:AB_10708720
Mouse monoclonal anti-Exosc10 (B8)	Santa Cruz	Cat#sc-374595, RRID:AB_10990273
Rabbit polyclonal anti-Dis3	Proteintech	Cat#14689-1-AP, RRID:AB_2091025
Mouse monoclonal anti-Dis3L (E-12)	Santa Cruz	Cat#sc-398739
Rabbit polyclonal anti-C1d	Proteintech	Cat#10711-1-AP, RRID:AB_2067014
Rabbit polyclonal anti-Mphosph6	Proteintech	Cat#10695-1-AP, RRID:AB_2282161
Rabbit polyclonal anti-Parp	Cell Signaling Technology	Cat#9542; RRID:AB_2160739
Rabbit monoclonal anti-Caspase3 (D3R6Y)	Cell Signaling Technology	Cat#14220, RRID:AB_2798429
Rabbit polyclonal anti-Cleaved Caspase3	Cell Signaling Technology	Cat#9661; RRID:AB_2341188
Rabbit monoclonal anti-Actin	Sigma-Aldrich	Cat#A2066, RRID:AB_476693
Mouse monoclonal anti-Tubulin	Proteintech	Cat#66240-1-Ig, RRID:AB_2881629
Rabbit polyclonal anti-Vinculin	Proteintech	Cat#26520-1-AP
Mouse monoclonal anti-Hsp70	Abcam	Cat#ab2787, RRID:AB_303300
Mouse monoclonal anti-V5	Thermo Fisher Scientific	Cat#R960-25, RRID:AB_2556564
Mouse monoclonal anti-SPOT	Proteintech	Cat#28a5, RRID:AB_2892245
Mouse monoclonal anti-FLAG	Sigma-Aldrich	Cat#F3165
Rat monoclonal anti-HA	Proteintech	Cat#7C9
HRP-conjugated goat polyclonal anti-mouse IgG (H + L)	Thermo Fisher Scientific	Cat#G-21040, RRID:AB_2536527
HRP-conjugated goat polyclonal anti-rabbit IgG (H + L)	Thermo Fisher Scientific	Cat#G-21234, RRID:AB_2536530
HRP-conjugated goat polyclonal anti-rat IgG (H + L)	Proteintech	Cat#SA00001-15
eFluor 450-conjugated rat monoclonal anti-CD9 (eBioKMC8)	Thermo Fisher Scientific	Cat#48-0091-82, RRID:AB_2574008)
**Oligonucleotides and other sequence-based reagents**		
Guide RNA oligonucleotides	This study	Table EV1
RT-qPCR oligonucleotides and Northern blot probes	This study	Table EV2
**Chemicals, Enzymes and other reagents**		
2-Mercaptoethanol	Sigma-Aldrich	Cat#M6250; CAS:60-24-2
Ammonium persulfate (APS)	Sigma-Aldrich	Cat#A3678; CAS:7727-54-0
BbsI-HF	New England Biolabs	Cat#R3539
Blasticidin S HCl	Gibco	Cat#A1113903
Doxycycline hyclate	Sigma-Aldrich	Cat#D9891; CAS: 24390-14-5
dTAGV-1 TFA	MedChemExpress	Cat#HY-145514; Cas:2624313-15-9
Epoxomicin	Gentaur Molecular Products	Cat#607-A2606; CAS:134381-21-8
Esp3I	New England Biolabs	Cat#R0734
Ethidium Bromide	Sigma-Aldrich	Cat#E1510; CAS:1239-45-8
Hoechst33342	Sigma-Aldrich	Cat#B2261; CAS:875756-97-1
Hygromycin B	Roche	Cat#10843555001; CAS:31282-04-9
Igepal CA-630	Sigma-Aldrich	Cat#I8896; CAS:9002-93-1
Indole-3-acetic acid sodium salt	Sigma-Aldrich	Cat#I5148; CAS:6505-45-9
MOPS (3-Morpholinopropanesulfonic Acid)	PanReacAppliChem	Cat#A1076; CAS:1132-61-2
N-Lauroylsarcosine sodium salt	Sigma-Aldrich	Cat#L5125; CAS:137-16-6
Ponceau S solution	Sigma-Aldrich	Cat#P7170; CAS 6226-79-5
Proteinase K	Promega	Cat#MC5005
Puromycin Dihydrochloride	Gibco	Cat#A1113803
Sodium dodecyl sulfate (SDS), 20% solution	Thermo Fisher Scientific	Cat#AM9820; CAS:151-21-3
TAK-243	ChemScence	Cat#CS-0019384; CAS:1450833-55-2
TEMED	Thermo Fisher Scientific	Cat#17919; CAS:110-18-9
Triton X-100	Sigma-Aldrich	Cat#T8787; CAS:9036-19-5
Trizol	Thermo Fisher Scientific	Cat#15596018
Tween-20	Sigma-Aldrich	Cat#655205; CAS:9005-64-5
Bio-Rad Protein Assay Dye Reagent Concentrate	Bio-Rad	Cat#5000006
Micro BCA Protein Assay kit	Thermo Fisher Scientific	Cat#23235
TURBO DNA-free Kit	Thermo Fisher Scientific	Cat#AM1907
SuperScript III Reverse Transcriptase	Thermo Fisher Scientific	Cat#18080085
GoTaq qPCR Master Mix	Promega	Cat#A6002
RNAseOUT	Thermo Fisher Scientific	Cat#10777019
Anti-FLAG M2 magnetic beads	Millipore	Cat#M8823
Anti-HA magnetic beads	Thermo Fisher Scientific	Cat#88837
V5-Trap Magnetic Agarose	Proteintech	Cat#v5tma; RRID:AB_2868498
SPOT-Trap Magnetic Agarose	Proteintech	Cat#etma; RRID:AB_2827591
Sera-Mag Carboxylate-Modified Magnetic Beads E3	Cytivia	Cat# 65152105050250
Sera-Mag Carboxylate-Modified Magnetic Beads E7	Cytivia	Cat# 45152105050250
Random primers	Thermo Fisher Scientific	Cat#48190011
NEBuilder HiFi DNA Assembly Master Mix	New England Biolabs	Cat#E2621
Halt Protease Inhibitor Cocktail, EDTA-Free	Thermo Fisher Scientific	Cat#78437
Restore Western blot stripping buffer	Thermo Fisher Scientific	Cat#21063
iST kit	PreOmics	Cat#P.O.00027
HeLa protein digest standard	Thermo Fisher Scientific	Cat#88329
4–20% Mini-PROTEAN TGX Precast Protein Gels	Bio-Rad	Cat#4561096
Clarity Western ECL Substrate	Bio-Rad	Cat#1705060
Fetal Bovine Serum	Sigma-Aldrich	Cat#F7524; Lot# BCBV4855
Fetal Bovine Serum	Gibco	Cat#10270-106
Hybond-N+ Membrane	Thermo Fisher Scientific	Cat#45-000-927
Immobilon-P Membrane, PVDF, 0.45 µm	EMD Millipore	Cat#IPVH00010
Gel Loading Buffer II	Thermo Fisher Scientific	Cat#AM8547
GlutaMAX Supplement	Gibco	Cat#35050061
L-Glutamine	Gibco	Cat#25030081
Lipofectamine 2000	Thermo Fisher Scientific	Cat#11668019
MEM non-essential amino acids (NEAA), 100x	Gibco	Cat#11140035
OPTI-MEM	Gibco	Cat#31985070
Penicillin-Streptomycin	Sigma-Aldrich	Cat#P0781
Polyethylenimine, Linear, MW 25000	Polysciences	Cat#23966-100; CAS:9002-98-6
Polybrene	Merck	Cat#TR-1003-G
Precision Plus Protein Dual Color Standards	Bio-Rad	Cat#1610394
ProtoGel (30%), 37.5:1 Acrylamide to Bisacrylamide Stabilized Solution	National Diagnostics	Cat#EC-890
Sodium pyruvate (100 mM)	Sigma-Aldrich	Cat#S8636; CAS:113-24-6
Quick-Load Purple 1 kb Plus DNA Ladder	New England Biolabs	Cat#N0550
RiboRuler High Range RNA Ladder	Thermo Fisher Scientific	Cat#SM1823
Trypan Blue Stain (0.4%)	Thermo Fisher Scientific	Cat#T10282
Trypsin-EDTA (0.5%), no phenol red	Gibco	Cat#15400054
**Software**		
VBC score	(Michlits et al, 2020)	https://www.vbc-score.org/
CHOPCHOP (v3)	(Labun et al, 2019)	RRID:SCR_015723; https://chopchop.cbu.uib.no/
ImageJ (v.2.14.0)	(Schindelin et al, 2012)	RRID:SCR_003070; https://imagej.net/ij/
Image Lab (v.5.1.1)	Bio-Rad	RRID:SCR_014210; https://www.bio-rad.com/de-at/product/image-lab-software?ID=KRE6P5E8Z
ImageQuant TL (v.10.2)	Cytiva	RRID:SCR_014246; https://www.cytivalifesciences.com/en/us/shop/protein-analysis/molecular-imaging-for-proteins/imaging-software/imagequant-tl-10-2-analysis-software-p-28619
CFX Manager (v.1.0)	Bio-Rad	RRID:SCR_017251; https://www.bio-rad.com/en-us/product/cfx-maestro-software-for-cfx-real-time-pcr-instruments
GraphPad Prism (v.10.1.1)	Graphpad Software	RRID:SCR_002798; https://www.graphpad.com/
SnapGene (v.7.1)	SnapGene	https://www.snapgene.com/
FlowJo (v.10.9.0)	Becton, Dickinson and Company	RRID:SCR_008520; https://www.flowjo.com/
R (v.4.3.1)	R Foundation for Statistical Computing	RRID:SCR_001905; https://www.r-project.org/
R Studio (v.2023.09.1 + 494)	Posit	RRID:SCR_000432; https://www.rstudio.com/
Adobe Photoshop (v.25.4.0)	Adobe	RRID:SCR_014199; https://www.adobe.com/ products/photoshop.html
Adobe Illustrator (v.27.9.1)	Adobe	RRID:SCR_010279; http://www.adobe.com/ products/illustrator.html
Proteome Discoverer (v. 2.5.0.400)	Thermo Fisher Scientific	RRID:SCR_014477; https://www.thermofisher.com/at/en/home/industrial/mass-spectrometry/liquid-chromatography-mass-spectrometry-lc-ms/lc-ms-software/multi-omics-data-analysis/proteome-discoverer-software.html
MS Amanda (v2.0.0.19924)	(Dorfer et al, 2014)	https://ms.imp.ac.at/?goto=msamanda
Percolator algorithm	(Käll et al, 2007)	https://github.com/percolator/percolator/wiki
ptmRS algorithm	(Taus et al, 2011)	https://ms.imp.ac.at/?action=ptmrs
IMP-apQuant	(Doblmann et al, 2018)	https://ms.imp.ac.at/?action=apQuant
MaxLFQ algorithm	(Cox et al, 2014)	N/A
Skyline (v. 22.2.0.351)	(MacLean et al, 2010)	RRID:SCR_014080; https://skyline.ms/project/home/software/Skyline/begin.view
**Other**		
Superdex 200 Increase 10/300 GL	Sigma-Aldrich	Cat#GE28-9909-44

### Experimental model and subject details

#### mES and Lenti-X cell culture and chemical treatments

All presented experiments were performed in feeder-independent mouse embryonic stem cells (mESCs), clone AN3-12 (Elling et al, 2017). The mESCs were cultured in high-glucose DMEM (in-house), supplemented with 13.5% fetal bovine serum (Sigma-Aldrich or Gibco), 1x Penicillin-Streptomycin (Sigma-Aldrich), 2 mM L-glutamine (Gibco) or 1x GlutaMax (Gibco), 1x MEM non-essential amino acid solution (Gibco), 1 mM sodium pyruvate (Sigma-Aldrich), 50 µM 2-mercaptoethanol, and 20 ng/ml LIF (in-house). Cells were kept at 37 °C with 5% CO_2_ and passaged every second day.

For antibiotic selection in mESCs, Hygromycin (Roche) was used at 250 µg/ml, Blasticidin S (Gibco) at 5 µg/ml, G418 (Gibco) at 800 µg/ml and Puromycin (Gibco) at 1 µg/ml. For Cas9 induction, cells were cultured in Doxycycline-containing media (Dox, Sigma-Aldrich) at 500 ng/ml for the indicated periods of time. For depletions in AID-tagged cell lines, indole-3-acetic acid sodium salt (IAA, Sigma-Aldrich) was used at 250 µM final for the indicated time-points. For depletions in dTAG-tagged cell lines, dTAGv-1 (MedChemExpress) was used at 500 nM final for the indicated time-points. For proteasome inhibition, Epoxomicin (Gentaur Molecular Products) was used at a final concentration of 1 µM, and TAK-243 (ChemScence) was used for Uba1 inhibition at a final concentration of 2.5 µM.

Lenti-X 293T cells (Clontech, 632180) were used only for lentivirus production. Cells were cultured in high-glucose DMEM (in-house), supplemented with 10% fetal bovine serum (Sigma-Aldrich), 2mM L-Glutamine (Gibco) or 1x GlutaMax (Gibco), 1x MEM non-essential amino acid solution (Gibco), 1 mM sodium pyruvate (Sigma-Aldrich), 50 µM 2-mercaptoethanol. Cells were kept at 37 °C with 5% CO_2_ and passaged every third day.

All cell lines were regularly tested to control for the absence of mycoplasma contamination.

#### Design and cloning of dual sgRNAs

The top-performing gRNA from the Vienna gRNA (Michlits et al, 2020) library was used to predict pairing gRNAs. All gRNAs targeting murine exosome genes were filtered stringently for off-targets, and polyA and restriction-site containing sequences were removed. Then, relative cutting positions to the original guide sequences were calculated and filtered for a window of ±50–1000 bp, and further filtered for out-of-frame cut site distances. Resulting matching gRNAs were ranked by VBC score, and the top guide was selected.

Dual gRNAs were ordered as oligonucleotides (see Table EV1), annealed, and cloned into pDual-hU6-sgRNA-mU6-sgRNA-EF1α-mCherry-P2A-PuroR expression vector using BbsI-HF (NEB) and Esp3I (NEB) restriction enzymes.

#### Cloning of constructs for exogenous expression

Expression vectors for the ectopic expression of Exosc1 and Exosc2 were based on the pRRL backbone (Addgene #145840). pRRL vector was modified to contain CMV promoter and Blasticidin S resistance cassette (only for Exosc2).

Full-length cDNA for Exosc1 (NM_025644.4) and Exosc2 (NM_144886.3) was prepared with reverse transcription from total RNA of wild-type mESCs, followed by a PCR amplification of the region containing an open reading frame. Fragments for mCherry, eBFP2, mScarlet3, and mNeonGreen were separately amplified, and linker sequence (3xGGGS), V5-tag, SPOT-tag, and HA-tag were introduced with the primers.

For the generation of a gRNA-resistant Exosc2 variant, synonymous mutations were introduced into the PAM site and seed sequence of the gRNA target region, without altering the Exosc2 amino acid sequence.

For the generation of the plasmid with Dox-inducible expression of Exosc7-3xFLAG-P2A-mCherry, full-length cDNA for Exosc7 (NM_001081188.2) was prepared with reverse transcription from total RNA of wild-type mESCs, followed by a PCR amplification of the region containing an open reading frame. The Exosc7 coding sequence was subsequently cloned into the donor vector, resulting in the final construct with the following configuration: Cbh-rtTA3-IRES-HygroR-insulator-insulator-mCherry-P2A-3xFLAG-3xGGGS-Exosc7-TRE3G-insulator-insulator.

All described constructs were assembled with NEBuilder HiFi DNA Assembly Master Mix (NEB) from the respective fragments and verified by Sanger sequencing. The list of plasmids is in the Key resource table.

#### Generation of Exosc1-KO cell line

To generate Exosc1-KO cells, gRNA targeting the second exon of Exosc1 was selected with a VBC score (Michlits et al, 2020). gRNA was cloned into the pLenti-CRISPR-v2-GFP vector as described before (Herzog et al, 2017). Prior to transfection with gRNA-containing plasmid, wild-type mESCs were stained with Hoechst33342 (Sigma-Aldrich) at 10 µg/ml for 25 min, followed by FACS-sorting (FACSAria III cell sorter, BD Biosciences) to obtain a haploid population. 5 × 10^5^ cells were reverse transfected with 2.5 µg of Exosc1 gRNA-containing plasmid using Lipofectamine 2000 (Thermo Fisher Scientific) according to the manufacturer protocol. Forty-eight hours post transfection, GFP-positive cells were FACS-sorted, and 2000 cells were seeded on a 15-cm dish. Single colonies were picked after 8 days and expanded. For genotyping, cells were lysed with DNA isolation buffer (10 mM Tris-HCl, pH 7.5, 10 mM EDTA, 10 mM NaCl, 0.5% *N*-lauroylsarcosine, 1 mg/ml Proteinase K) at 65 °C for 2 h followed by 95 °C for 15 min. About 1 µl of the extracted genomic DNA was added to 20 µl of the PCR reaction with primers flanking the edited loci. PCR products were Sanger sequenced, and for selected clones, the absence of Exosc1 was confirmed with Western blot and targeted PRM mass-spectrometry. Cells were stained with Hoechst33342 (Sigma-Aldrich) and diploid FACS-sorted prior to other experiments.

The gRNA sequence used to generate Exosc1-KO is indicated in Table EV1.

#### Generation and validation of iCas9v1 cell line

As a parental cell line for iCas9v1 generation, we used previously generated in the lab for other purposes OsTir1-3xMyc-T2A-eYFP expressing Xrn1-L-AID-V5 cells. About 300k cells were seeded on a 6-well plate and sequentially transduced with pRRL-SFFV-rtTA3-IRES-EcoRec-PGK-Hygro and pRRL-TRE3G-Cas9-P2A-eBFP2 and selected with Hygromycin B. Next, Doxycycline (Dox, Sigma-Aldrich) was added to induce eBFP2 expression and eBFP2+ cells were FACS-sorted, followed by single cell clone isolation as described in the “Generation of Exosc1-KO mESCs cell line” section. To assess the function of Cas9 and the tightness of the TRE3G promoter in the selected clones, cells were transduced with the construct expressing gRNA eYFP and Cas9 expression was induced with Dox (Sigma-Aldrich). All measurements were performed with flow cytometry using iQue Screener PLUS (Intellicyt) or LSRFortessa (BD Biosciences).

#### Generation of iCas9v2 cell line

For the generation of iCas9v2, we inserted an all-in-one cassette, containing SFFV-rtTA3-IRES-HygroR-insulator-insulator-iRFP713-P2A-Cas9-TRE3G, into the expression-stable locus on Chr15 (mESCs: chr15 safe-harbor) (Moussa et al, 2019) with Flp-recombinase mediated cassette exchange (RMCE). A total of 5 × 10^5^ cells were reverse co-transfected with a plasmid containing the cassette flanked by FRT/F3 sites (pYR03-FRT-Cbh-rTetR-IH-ins2-iRFP713-P2A-Cas9-TRE3G-ins2-F3), and a plasmid expressing Flp (pCAG-Flp-GFP) at an equimolar ratio, using Lipofectamine 2000 according to the manufacturer's instructions. Forty-eight hours post transfection, GFP-positive cells were FACS-sorted and passaged for 10 days. Afterward, BFP-negative cells were FACS-sorted, and single clones were isolated as described in the “Generation of Exosc1-KO mESCs cell line” section. Single clones were genotyped using integration site-specific PCR and Sanger sequencing. To assess the function of Cas9 and the tightness of the TRE3G promoter, clones were transduced with the construct expressing gRNAs against CD9 and GFP, followed by Cas9 induction and CD9-immunostaining. All measurements were performed with flow cytometry using LSRFortessa (BD Biosciences) and Penteon (Novocyte).

#### Generation of endogenously tagged AID-Exosc3 and Exosc6-3xGGGS-AID-V5 cell lines

A parental cell line expressing Tir1 E3-ligase was generated by inserting SFFV-Tir1-3xMyc-T2A-eYFP into Rosa26 loci by Flp-recombinase mediated cassette exchange (RMCE) into donor AN3-12 mESCs cells (GeneTrap Gt(Rosa26)Sor, Haplobank). To this end, 5 × 10^5^ cells were reverse co-transfected with a plasmid containing Tir1 flanked by FRT/F3 sites (pYR03_FRT-SFFV-OsTir1-3xMyc-T2A-eYFP-F3), a plasmid expressing Flp (pCAG-Flp-GFP) at equimolar ratio, using Lipofectamine 2000 according to the manufacturer instruction. Seven days after transfection, eYFP+cells were FACS-sorted, and single clones were isolated as described in the “Generation of Exosc1-KO mESCs cell line” section. Single clones were genotyped using integration site-specific PCR and Sanger sequencing. Cells were stained with Hoechst33342 (Sigma-Aldrich) and haploid FACS-sorted before proceeding with the endogenous tagging of Exosc3 and Exosc6.

For the endogenous tagging of Exosc3 and Exosc6, gRNAs targeting the N-terminus of Exosc3 and the C-terminus of were predicted with the CHOPCHOP tool (v.3) (Labun et al, 2019) and cloned into pX330-CMV-Cas9-hGem-P2A-mCherry, which was modified from pX330-CMV-Cas9-hGem (Addgene # 71707) by addition of P2A-mCherry. 5’- and 3’-homology arms were amplified from the genomic DNA and cloned along with AID-tag and V5-tag (for Exosc6) in pCR4-TOPO (Invitrogen) backbone. Next, haploid OsTir1-3xMyc-T2A-eYFP cells were reverse co-transfected (Lipofectamine 2000, Thermo Fisher Scientific) with an equimolar ratio of gRNA-expressing plasmid and a homology template plasmid (5’–homology arm–AID-tag–3’-homology arm; pCR4-TOPO-AID-Exosc3-HT). For the endogenous C-terminal tagging of Exosc6 with linker-AID-V5-tag, haploid Tir1 cells were reverse co-transfected (Lipofectamine 2000, Thermo Fisher Scientific) with gRNA-expressing plasmid and the homology template plasmid (5 –homology arm–3xGGGS linker-AID-tag–V5-tag–3’-homology arm; pCR4-TOPO-Exosc6-L-AID-V5-HT) at 1:1 molar ratio. Forty-eight hours post transfection, mCherry-positive cells were FACS-sorted, and single clones were isolated as described in the “Generation of Exosc1-KO mESCs cell line” section. Successful endogenous tagging was confirmed by integration site-specific PCR, Sanger sequencing and Western blot. Note that AID-tagging of Exosc3 resulted in a modest but measurable reduction in Exosc3 protein levels compared to the parental cell line (Fig. EV1E), in line with a previous report of Exosc3 AID-tagging (Gockert et al, 2022).

Sequences of gRNAs used for endogenous AID-tagging of Exosc3 and Exosc6 are indicated in Table EV1.

#### Generation of endogenously tagged Exosc7-, Exosc8-, and Exosc9-3xGGGS-dTAG-V5 cell lines

For endogenous C-terminal tagging of Exosc7, Exosc8 and Exosc9, gRNAs targeting the C-termini of the respective genes were designed with CHOPCHOP tool (v.3) (Labun et al, 2019) and cloned into pX330-CMV-Cas9-hGem-P2A-mTagBFP2, which was modified from pX330-CMV-Cas9-hGem (Addgene # 71707) by addition of P2A-mTagBFP2. 5’- and 3’-homology arms were amplified from the genomic DNA and cloned into donor backbones containing either GGGS3-dTag-lox5171-IRES-mScarlet3-lox5171 or GGGS3-dTag-lox5171-IRES-mNeonGreen-lox5171 sequence. Parental iCas9v2 cells were reverse co-transfected with an equimolar ratio of the gRNA-expressing plasmid and a 1:1 mixture of the homology donor plasmids using Lipofectamine 2000 (Thermo Fisher Scientific). Forty-eight hours post transfection, mTagBFP2-positive cells were isolated by FACS and passaged for 12 days. Subsequently, a second round of FACS-sorting was performed to isolate double-positive mScarlet3/mNeonGreen cells, which were expanded for an additional 7 days. Cells were then transfected (Lipofectamine 2000, Thermo Fisher Scientific) with a Cre recombinase-expressing plasmid (pCMV_NLS-Cre-HSVTKpA) to excise the IRES-mScarlet3 and IRES-mNeonGreen cassettes, passaged for 7 days and subjected to double negative mScarlet3/mNeonGreen FACS-sorting. Single clones were isolated as described in the “Generation of Exosc1-KO mESCs cell line” section. Successful endogenous tagging was confirmed by integration site-specific PCR, Sanger sequencing and Western blot.

Sequences of gRNAs used for endogenous dTAG-tagging of Exosc7, Exosc8, and Exosc9 are indicated in Table EV1.

#### Generation of mESCs with doxycycline-inducible expression of Exosc7-3xGGGS-3xFLAG-P2A-mCherry

To generate mESCs carrying the TRE3G-Exosc7-L-3xFLAG-P2A-mCherry construct, an all-in-one cassette containing Cbh-rtTA3-IRES-HygroR-insulator-insulator-mCherry-P2A-3xFLAG-3xGGGS-Exosc7-TRE3G was inserted into the expression-stable locus on chromosome 15 (mESCs: chr15 safe-harbor) (Moussa et al, 2019) using Flp-recombinase mediated cassette exchange (RMCE). About 5 × 10^5^ cells were reverse co-transfected with a plasmid containing the cassette flanked by FRT/F3 sites (pYR03-FRT-Cbh-rTetR-IH-ins2-imCherry-P2A-3xFLAG-3xGGGS-Exosc7-TRE3G-ins2-F3), and a plasmid expressing Flp (pCAG-Flp-GFP) at equimolar ratio, using Lipofectamine 2000 (Thermo Fisher Scientific) according to the manufacturer instruction. Forty-eight hours post transfection, GFP-positive cells were FACS-sorted and passaged for 10 days. Afterward, BFP-negative cells were FACS-sorted, and single clones were isolated as described in the “Generation of Exosc1-KO mESCs cell line” section. The tightness and induction of the Exosc7 expression in single clones was assessed with flow cytometry using LSRFortessa (BD Biosciences) and Penteon (Novocyte).

#### Generation of mESCs with knockout-rescue of Exosc2-3xGGGS-HA-mNeonGreen

To generate a knockout-rescue cell line expressing Exosc2-3×GGGS-HA-mNeonGreen, parental iCas9v2 cells were lentivirally transduced with a construct encoding a gRNA-resistant version of Exosc2 (pRRL-CMV-Exosc2-L-HA-mNeonGreen-IB_gRNA_resistant) and selected with Blasticidin S (Gibco) for 6 days. Cells were subsequently transfected with a plasmid expressing a gRNA targeting Exosc2 in the pLenti-CRISPR-v2-mCherry backbone using Lipofectamine 2000 (Thermo Fisher Scientific). 48 h post-transfection, mCherry-positive cells were isolated by fluorescence-activated cell sorting (FACS), and single-cell clones were derived as described in the “Generation of Exosc1-KO mESC cell line” section. Selected clones were validated by Western blot analysis.

#### Surface marker immunostaining

Cells were pelleted (200 g, 3 min, 4 °C) and stained with eFluor450-conjugated anti-CD9 antibody (1:500, Thermo) in FACS buffer (1x PBS, 5% FBS) for 10 min at 4 °C. Cells were washed once with FACS buffer, spun down, resuspended in FACS buffer, and analyzed with flow cytometry using LSRFortessa (BD Biosciences) and Penteon (Novocyte).

#### Competitive proliferation assays

Proliferation analyses of inducible knockouts were performed as follows: dual gRNA mCherry+ iCas9 cells were mixed with a parental iCas9 cell line, Dox was added to induce Cas9 expression and a fraction of mCherry+ cells was monitored over time with flow cytometry.

For growth competition experiments in AID-tagged cell lines (in Tir1-T2A-eYFP background), AID-Exosc3 and Exosc6-AID cells were co-cultured with wild-type cells in the absence/presence of indol-3-acetic acid (IAA, Sigma-Aldrich). The percentage of eYFP+cells was followed over time with flow cytometry.

To address proliferation defects of monoclonal Exosc1-KO and rescue cell lines, respective cell lines were co-cultured with OsTir1-3xMyc-T2A-eYFP cells and the percentage of eYFP- cells was derived from flow cytometry measurements.

All measurements were performed with iQue Screener PLUS (Intellicyt) or LSRFortessa (BD Biosciences). Data analysis was done with FlowJo (v.10.9.0) software.

#### Lentivirus production and infections

For lentiviral production, 4 × 10^5^ Lenti-X cells were plated on a six-well plate and transfected the next day. To prepare a transfection mixture, 2300 ng of donor plasmid (i.e., expressing dual sgRNAs) were mixed with 1140 ng pCMVR8.74 (Addgene #22036), 670 ng pMD2.G (Addgene #12259), 12 µl polyethylenimine (PEI, Polysciences) in 200 µl OPTI-MEM (Gibco). The transfection mix was vortexed, incubated for 25 min at room temperature and added dropwise to the cells. Media was changed 4–10 h after transfection and lentiviruses were harvested 24 h later. Viral supernatant was filtered through a 0.45 µm filter and used for infection of mESCs in the presence of 4 µg/ml polybrene (Merck). The list of plasmids used for lentiviral transduction can be found in the Key Resource Table.

#### Western blotting

Whole cell protein extracts were prepared by resuspending cells in the ice-cold Western blot lysis buffer (50 mM Tris-HCl, pH 7.5, 150 mM NaCl, 1% Triton X-100, 0.5% Igepal CA-630, 0.1% SDS, 0.5 mM EDTA) supplemented with 1x fresh HALT protease inhibitors (Thermo). Lysates were incubated on ice for 30 min and spun down at 20,000 × *g*, 4 °C for 20 min. Supernatant, containing total protein lysate, was collected, and its concentration was determined with Bio-Rad Protein Assay Dye Reagent (Bio-Rad) according to the manufacturer’s instructions. About 50–150 µg of total protein were mixed with 6X loading buffer (375 mM Tris-HCl, pH 6.8, 12% SDS, 60% glycerol, 6% 2-mercaptoethanol, bromphenol blue) and denatured at 95 °C for 5 min. Samples were separated either on self-made acrylamide gels (5% stacking and 12% separating) or on 4–20% pre-cast gradient gels (Bio-Rad) and transferred to Immobilon-P PVDF membrane (EMD Millipore) at 100 V for 1 h using a wet-chamber system. Membranes were blocked with 3% non-fat milk in TBS + 0.05% Tween-20 (TBST) for 1 h at room temperature and incubated with primary antibodies at 4 °C overnight. The next day, membranes were washed 5 × 5 min with TBST and HRP-secondary antibody were added for 1 h at room temperature, followed by 5 × 5 min TBST washes. Proteins were detected using Clarity Western ECL Substrate (Bio-Rad) with ChemiDoc Imaging System (Bio-Rad) using ImageLab V5.1.1 (Bio-Rad). For the membranes that were probed with several different antibodies, membranes were incubated in the Restore Western blot stripping buffer (Thermo) at room temperature for 20 min followed by 3 × 5 min TBST washes, re-blocking and incubation with primary/secondary antibody.

The following antibodies were used (also see the key resource table): rabbit anti-Exosc1 (1:5000, Bethyl), rabbit anti-Exosc2 (1:2000, Bethyl), rabbit anti-Exosc2 (1:5000, Proteintech), mouse anti-Exosc2 (1:3000, Proteintech), rabbit anti-Exosc3 (1:1000, Novus), rabbit anti-Exosc3 (1:5000, Proteintech), rabbit anti-Exosc4 (1:5000, Proteintech), mouse anti-Exosc4 (1:3000, Santa Cruz), rabbit anti-Exosc5 (1:2000, Proteintech), rabbit anti-Exosc6 (1:5000, Thermo), mouse anti-Exosc6 (1:1000, Santa Cruz), mouse anti-Exosc7 (1:200, Santa Cruz), rabbit anti-Exosc7 (1:10,000, Proteintech), rabbit anti-Exosc8 (1:3000, Proteintech), rabbit anti-Exosc9 (1:3000, Proteintech), mouse anti-Exosc9 (1:3000, Proteintech), mouse anti-Exosc9 (1:1000, Santa Cruz), mouse anti-Exosc10 (1:1000, Santa Cruz), rabbit anti-Dis3 (1:3000, Proteintech), mouse anti-Dis3L (1:100, Santa Cruz), rabbit anti-C1d (1:1000, Proteintech), rabbit anti-Mphosph6 (1:1000, Proteintech), rabbit anti-Parp (1:5000, Cell Signalling), rabbit anti-Caspase3 (1:3000, Cell Signalling), rabbit anti-Cleaved Caspase3 (1:2000, Cell Signalling), rabbit anti-Actin (1:5000, Sigma-Aldrich), mouse anti-Tubulin (1:20000, Proteintech), rabbit anti-Vinculin (1:10,000, Proteintech), mouse anti-Hsp70 (1:1000, Abcam), mouse anti-V5 (1:5000, Thermo), mouse anti-SPOT (1:5000, Proteintech), mouse anti-FLAG (1:10,000, Sigma Aldrich), rat anti-HA (1:5000, Proteintech) HRP-conjugated anti-mouse (1:10,000, Thermo), HRP-conjugated anti-rabbit (1:10,000, Thermo), HRP-conjugated anti-rat (1:5000, Proteintech).

Western blot quantifications were done with ImageJ software (v. 2.14.0), taking into account the linear range of chemiluminescence signal (Pillai-Kastoori et al, 2020) as judged from the dilution series (100, 50, 25, and 10%). Often the band was not visible in the lowest 10% dilution sample, thus we fixed 10% as the detection limit of our Western blot analyses and reported the maximum depletion efficiency of being less or equal 10%. For all the quantifications, the signal of a protein of interest was normalized to the loading control (Hsp70, actin, tubulin, or vinculin) and presented relative to control samples.

#### Size-exclusion chromatography (SEC)

A total of 12 × 10^6^ mESCs cells per dish were seeded on three 15-cm dishes 24 h prior to harvest. Cells were resuspended in ice-cold IP lysis buffer (50 mM Tris-HCl, pH 7.5, 150 mM NaCl, 1% Igepal CA-630, and 0.5 mM EDTA) supplemented with 1x freshly added HALT protease inhibitors (Thermo). Lysates were incubated on ice for 30 min, sonicated (40% power, three cycles, 10 s, Bandelin Sonoplus HD2070) and centrifuged at 20000 × *g*, 4 °C for 20 min. The supernatant containing total protein lysate was collected, filtered through a 0.22 µm filter and protein concentration was determined using the Bio-Rad Protein Assay Dye Reagent (Bio-Rad) according to the manufacturer’s instructions. About 2 mg of filtered lysate were adjusted to a final volume of 500 µl with IP lysis buffer and loaded on a Superdex 200 increase 10/300 GL size-exclusion chromatography column (Sigma-Aldrich), equilibrated in running buffer (50 mM Tris-HCl, pH 7.5, 150 mM NaCl, and 0.1% Igepal CA-630). Chromatography was performed at a flow rate of 0.5 ml/min. UV absorbance was monitored at 260 and 280 nm, and fractions of 0.3 ml were collected.

For downstream western blot analysis, SEC fractions were concentrated using the SP3 protocol as previously described (Hughes et al, 2019). Briefly, fractions were concentrated to a final volume of 100 µl using a SpeedVac machine (Concentrator plus, Eppendorf, 45 °C, V-AQ mode). A mixture of pre-washed (miliQ water) Sera-Mag carboxylate-modified magnetic beads (3 µl of E3 and 3 µl of E7; (Cytivia; 50 mg/ml) was added to each sample and mixed. Subsequently, 150 µl of 100% ethanol were added, and samples were incubated at 25 °C for 10 min with shaking at 900 rpm. Beads were collected using a magnetic rack and washed four times with 200 µl of 80% ethanol. Bound proteins were eluted by adding 60 µl of 2X loading buffer, followed by incubation at 95 °C for 10 min with shaking at 900 rpm.

For immunoprecipitation experiments, the indicated size-exclusion fractions (A7–A10, B3–B6, and B8–B11) were pooled, Igepal CA-630 was adjusted to a final concentration of 1%, and fresh HALT protease inhibitors were added. Samples were then subjected to anti-HA-immunoprecipitation as described in the following section.

#### Immunoprecipitation experiments

Cells were resuspended in ice-cold IP lysis buffer (50 mM Tris-HCl, pH 7.5, 150 mM NaCl, 1% Igepal CA-630, and 0.5 mM EDTA) supplemented with 1x fresh HALT protease inhibitors (Thermo). Lysates were incubated on ice for 30 min, sonicated (40% power, 3 cycles, 10 s, Bandelin Sonoplus HD2070) and centrifuged at 20,000 × *g*, 4 °C for 20 min. Supernatant, containing total protein lysate, was collected, and its concentration was determined with Bio-Rad Protein Assay Dye Reagent (Bio-Rad) according to the manufacturer’s instructions. About 1 mg of total protein was mixed with either 25 µl of V5-Trap Magnetic Agarose (Proteintech), 20 µl of SPOT-Trap Magnetic Agarose (Proteintech), 20 µl of FLAG magnetic beads (Millipore), or 20 µl of HA magnetic beads (Thermo Fisher Scientific) and incubated at 4 °C for 3–4 h with end-to-end mixing. Beads were collected on a magnetic rack, washed four times with IP lysis buffer, and proteins were eluted with 2X loading buffer at 95 °C for 10 min. Input and bead-bound proteins were resolved on 4–20% pre-cast gels (Bio-Rad), and Western blotting was performed as described above.

For mass spectrometry analysis of immunoprecipitation samples, following the final wash with IP lysis buffer, beads were additionally washed five times with buffer containing 50 mM Tris-HCl (pH 7.5) and 150 mM NaCl. After the last wash, the buffer was completely removed and the beads were snap-frozen in liquid nitrogen. Samples were subsequently analyzed by MS following on-bead digestion of bound protein complexes.

#### RNA isolation and RT-qPCR

Total RNA was isolated using 1 ml of Trizol (Thermo) according to the provider’s protocol. Isolated RNA was subjected to Turbo DNAse (Thermo) treatment following the manufacturer’s instructions. About 2 µg of total RNA were used for cDNA preparation with Superscript III reverse transcriptase (Thermo) and a mix of 20 µM oligo dT20 and 50 ng/µl random primers (Thermo). qPCR was performed with GoTaq qPCR Master Mix (Promega) using CFX 384 Touch (Bio-Rad) Real-Time PCR with Bio-Rad CFX Manager (v.1.0) software. In all cases, melting curves were assessed, and selected products were run on an agarose gel to ensure that only a specific product was amplified. qPCR primers are listed in Table EV2.

qPCR signal for targets of interest was quantified using the 2^−ΔΔCt^ method (Livak and Schmittgen, 2001), normalized to an internal control (Gapdh mRNA) and presented as a fold change relative to the control sample.

#### Northern blotting

About 8 µg of total RNA were resolved on 1.2% agarose gel containing 1x MOPS buffer (40 mM MOPS, 10 mM sodium acetate, 1 mM EDTA; pH of the whole buffer was adjusted to 7.2) and 2% formaldehyde. Before loading on the gel, RNA was mixed with 2X loading buffer (2x MOPS buffer, 9% formaldehyde, 0.3x gel loading buffer II (Thermo), and 20 µg/ml ethidium bromide), incubated at 75 °C for 10 min and quickly chilled on ice. After the run, RNA was transferred onto N+ Hybond membrane (Thermo) by an overnight capillary transfer. The membrane was UV cross-linked (Auto mode of UV Stratalinker 2400, twice) and pre-hybridized with a Church Buffer (0.5 M Na_2_HPO_4_/NaH_2_PO_4_, pH 7.2, 1 mM EDTA, and 7% SDS) at 65 °C for 1 h, followed by the addition of the radiolabeled probe and overnight incubation at 37 °C unless indicated otherwise. The next day, the membrane was washed 3 × 15 min with 1X SSC (150 mM NaCl, 15 mM sodium citrate; pH of the whole buffer was adjusted to 7.0) + 0.1% SDS buffer and radiation was recorded with a phosphor screen (GE Healthcare) for 24–48 h. Imaging was done on an Amersham Typhoon phosphor imager (Cytiva)*.* To proceed with the next probe, membranes were incubated in 0.1% SDS at 95 °C for 15 min, followed by pre-hybridization and incubation with a new radiolabeled probe. Probes were used in the following order: ITS2_probe (incubation at 42 °C) => Pre-18S_probe => 7S_B_ probe => 7SK probe => mix of 5.8S, 18S, 28S probes. Northern blot probes are listed in Table EV2.

Northern blots were quantified using ImageQuant TL (v.10.2) software. Intensities of bands of interest were normalized to a loading control (7SK RNA) and presented relative to control samples.

#### Peptide preparation for mass spectrometry

For Exosome inducible knockout experiments, cells were seeded on a six-well plate and treated for 72 h with Dox. For monoclonal Exosc1-KO experiments, wild-type and Exosc1-KO clones were seeded on a six-well plate 24 h before the harvest. Next, cells were washed twice with 1X PBS, harvested with a cell scraper and spun down at 200 × *g* for 3 min. Cell pellet was frozen in liquid nitrogen and stored at −80 °C. Peptides for mass-spectrometry analysis were prepared with the iST kit (Preomics) according to the manufacturer’s instructions with slight modifications. Frozen cell pellets were incubated with 50 µl of LYSE at 95 °C, 10 min, 1000 rpm. Lysates were sonicated for 30 s (ten cycles, 50% amplitude, UP100H, Hielscher) and total protein concentration was determined with Micro BCA kit (Thermo). About 100 µg of total protein were mixed with 50 µl DIGEST and incubated overnight at 37 °C. The next day, digestion was quenched with 100 µl of STOP solution and the sample was applied to a cartridge, followed by WASH1 and WASH2. Cleaned peptides were eluted twice with 100 µl of ELUTE and placed in the SpeedVac machine (Concentrator plus, Eppendorf, 45 °C, V-AQ mode) until all the liquid evaporated. The sample was resuspended in 100 µl of 0.1% trifluoroacetic acid (TFA) and sonicated in an ultrasonic bath (Bandelin Sonorex super RK31) for 5 min to solubilize the peptides.

Final peptide amount was determined by separation of an aliquot of the sample on an LC-UV system equipped with a monolith column (Thermo) based on the peak area of 100 ng of HeLa protein digest standard (Thermo). The peptide solution was frozen at −80 °C before further processing.

#### Shotgun LC-MS/MS analysis

The nano-HPLC system UltiMate 3000 RSLC was coupled to an Orbitrap Exploris 480 mass spectrometer, equipped with a FAIMS pro interface and a Nanospray Flex ion source (all parts Thermo). Peptides were loaded onto a trap column (PepMap C18, 5 mm ×  300 μm ID, 5 μm particles, 100 Å pore size, Thermo). The trap column was switched in line with the analytical column (PepMap C18, 75 μm ID × 50 cm, 2 μm, 100 Å, Thermo). The analytical column was connected to PepSep sprayer 1 (Bruker) equipped with a 10 μm ID fused silica electrospray emitter with an integrated liquid junction (Bruker, Part#1893527). Electrospray voltage was set to 2.4 kV. Peptides were eluted using a flow rate of 230 nl/min, starting with the mobile phases 98% A (0.1% formic acid in water) and 2% B (80% acetonitrile, 0.08% formic acid) and linearly increasing to 35% B over the next 180 min, followed by a gradient to 95% B in 5 min, staying there for 5 min. Column equilibration was done at 30 °C with 2% B for 25 min for U3000.

The Orbitrap Exploris 480 mass spectrometer was operated in data-dependent mode, performing a full scan (m/z range 350–1200, resolution 60,000, normalized AGC target 100%) at three different compensation voltages (CV −45, −60, −75), followed by MS/MS scans of the most abundant ions for a cycle time of 0.8. MS/MS spectra were acquired using a collision energy of 30, isolation width of 1.0 m/z, resolution of 15,000, max fill time 100 ms, normalized AGC target of 100% and intensity threshold of 2.5 × 10^4^. Precursor ions selected for fragmentation (include charge state 2–6) were excluded for 40 s. The monoisotopic precursor selection (MIPS) filter and the exclude isotopes feature were enabled.

#### Proteomics data analysis

Raw MS data were loaded into Proteome Discoverer (v. 2.5.0.400). All MS/MS spectra were searched using MS Amanda (v2.0.0.19924)(Dorfer et al, 2014). Trypsin was specified as a proteolytic enzyme cleaving after lysine and arginine without proline restriction, allowing for up to two missed cleavages. Mass tolerances were set to ±10ppm at the precursor and fragment mass level. Peptide and protein identification was performed in two steps. An initial search was performed against the reference database (UP000000589, 21,962 sequences; 11,728,099 residues), with common contaminants appended. Here, carbamidomethylation of cysteine was searched as a fixed modification, whereas oxidation of methionine, deamidation of asparagine and glutamine and glutamine to pyro-glutamate conversion at peptide N-termini were defined as variable modifications. Results were filtered for a minimum peptide length of seven amino acids and 1% FDR at the peptide spectrum match (PSM) and the protein level using the Percolator algorithm (Käll et al, 2007) integrated in Proteome Discoverer. Additionally, an Amanda score of at least 150 was required.

A sub-database of proteins identified in this search was generated and used for a second search, where the RAW-files were analyzed using the same settings as above, plus considering additional variable modifications: phosphorylation of serines, threonines and tyrosines, acetylation on protein N-Terminus. The localization of the post-translational modification sites within the peptides was performed with the tool ptmRS, based on the tool phosphoRS (Taus et al, 2011). Identifications were filtered using the criteria described above, including an additional minimum PSM-count of 2 per protein in at least one sample. The identifications were subjected to label-free quantification using IMP-apQuant (Doblmann et al, 2019). Proteins were quantified by summing unique and razor peptides and applying intensity-based absolute quantification (iBAQ) (Schwanhäusser et al, 2011) with subsequent normalization based on the MaxLFQ algorithm (Cox et al, 2014). Identified proteins were filtered to contain at least 3 quantified peptide groups. Statistical significance of differentially expressed proteins was determined using limma (Smyth, 2004). GO-term enrichment analysis was done with the clusterProfiler R package (Yu et al, 2012).

All proteomics samples were measured in biological triplicates. For inducible knockout of Exosc5 (sgExosc5; Fig. 2B) and 90 min timepoint of Dox-Exosc7-3xFLAG pull-down experiments (Fig. 3J), one of the three replicates was classified as an outlier (based on normalization factors and PCA analysis) and excluded from the downstream analysis.

For the relative RNA exosome subunit quantification in Fig. 3I, iBAQ values were used according to the previously described procedure and presented relative to the heavy A7–A10 fractions (Smits et al, 2013).

Results of the proteome comparison between inducible knockouts of Exosc1-Exosc10, Dis3, Dis3L, Olfr10, and Olfr1 are listed in Dataset EV1.

Results of the mass-spectrometry analysis of Exosc2-V5 pull-down are listed in Dataset EV2.

Results of the mass-spectrometry analysis of Exosc2-HA pull-down from the size-exclusion fractions are listed in Dataset EV3.

Results of the mass-spectrometry analysis of doxycycline-inducible Exosc7-3xFLAG are listed in Dataset EV4.

Results of the proteome comparison between wild-type and Exosc1-KO are listed in Dataset EV5.

#### Targeted mass spectrometry via parallel reaction monitoring (PRM)

Vanquish Neo UHPLC-System or UltiMate 3000 RSLC coupled to the Orbitrap Exploris 480 mass spectrometer, equipped with an Easy spray Source (Thermo) were used. Peptides were loaded onto a trap column (PepMap C18, 5 mm × 300 μm ID, 5 μm particles, 100 Å pore size, Thermo) by using 0.1% TFA. The trap column was switched in line with the analytical column (PepMap C18, 500 mm × 75 μm ID, 2 μm, 100 Å, Thermo). The analytical column was connected to PepSep sprayer 1 (Bruker) equipped with a 10μm ID fused silica electrospray emitter with an integrated liquid junction (Bruker, Part#1893527). Electrospray voltage was set to 2.1 kV. Peptides were eluted at 230 nl/min using a binary 90-min gradient within the complete 135-min LC-MS/MS run, including column equilibration.

The gradient starts with the mobile phases: 98% A (water/formic acid, 99.9/0.1, v/v) and 2% B (water/acetonitrile/formic acid, 19.92/80/0.08, v/v/v), increases to 35% B over the next 90 min, followed by a gradient in 5 min to 90% B. Column equilibration was done with 2% B by 3 analytical column volumes in case of Neo Vanquish or for 25 min in case of U3000 at 30 °C.

The Orbitrap Exploris 480 mass spectrometer was operated by a mixed MS method, which consisted of one full scan (m/z range 380–1500; resolution 15,000; target value 100%) followed by the PRM of targeted peptides from an inclusion list (isolation window 0.8 m/z; normalized collision energy (NCE) 32; resolution 30,000, AGC target 200%). The maximum injection time was set to 800 ms.

A scheduled PRM method (sPRM) development, data processing and manual evaluation of results were performed in Skyline (v. 22.2.0.351) (MacLean et al, 2010). Spectra of unique peptides of the proteins of interest (Exosc1, Exosc2, Exosc3, Dis3L, and C1d) and normalization controls (Cct8, Eef2, Atp5f1b, Ncl, Pkm, and L1td1) were recorded. Each peptide used for the relative quantification of a protein of interest had to have at least three fragment ions.

For each protein of interest and normalization control, measured peptide areas were summed up. The obtained value for each protein of interest was divided by the value corresponding to the individual normalization control and presented relative to the respective control sample (wild-type for Exosc1-KO and sgOlfr1 for Exosome inducible knockout experiments). Median value (out of all normalization controls) was used as a relative estimation of the protein abundance.

For Fig. 2B, PRM was used to determine the relative protein abundance of Exosc1, Exosc2, Exosc3, and Dis3L. In sgExosc2 (two out of three replicates), sgExosc7 and sgExosc8 samples, no signal for Dis3L peptides was detected. Consequently, the Dis3L abundance was set to the maximum observed co-depletion.

For Fig. EV5B, PRM was used to determine the relative protein abundance of Exosc1, Exosc3, Dis3L, and C1d.

Results of the PRM measurements in inducible knockouts of Exosc1-Exosc10, Dis3, Dis3L, Olfr10, and Olfr1 are listed in Dataset EV1.

Results of the PRM measurements in wild-type and Exosc1-KO are listed in Dataset EV5.

#### Quantification and statistical analysis

Quantification methodologies of Western blots, northern blots, qPCRs and mass-spectrometry are described in the respective sections. Statistical analyses are indicated in the figure legends. Exact *p* values are reported in the figure legends where applicable; values below 1 × 10^−15^ are denoted as <1 × 10^−15^ due to limitations of GraphPad Prism.

## Supplementary information

Table EV1

Table EV2

Peer Review File

Data Set EV1

Data Set EV2

Data Set EV3

Data Set EV4

Data Set EV5

Expanded View Figures

## Data Availability

The MS proteomics data have been deposited to the ProteomeXchange Consortium via the PRIDE partner repository (Perez-Riverol et al, 2022) with the dataset identifier PXD061800. The proteomics data reported in this paper are also available as Datasets EV1–EV5. The source data of this paper are collected in the following database record: biostudies:S-SCDT-10_1038-S44318-026-00824-x.
