# Supplementary material for: Cellular assembly and functional resilience of the mammalian RNA exosome
Source: EMBO J. 2026 Jun 25;45(15):5423–56. doi: 10.1038/s44318-026-00824-x (PMC13434269; doi:10.1038/s44318-026-00824-x)
Supplement: Supplementary file 1 [file 44318_2026_824_MOESM3_ESM.pdf]

THE EMBO JOURNAL

Cellular Assembly and functional Resilience of the mammalian RNA Exosome

Tsimafei Navalayeu, Nikolaus Beer, Monika Bebjakova, Robert Kalis, Ulrich Hohmann, Karel Stejskal, Gabriela Krššáková, Nina Fasching, Veronika Herzog, Niko Popitsch, Elisabeth Roitinger, Clemens Plaschka, Johannes Zuber, and Stefan Ameres

Corresponding author(s): Stefan Ameres (stefan.ameres@maxperutzlabs.ac.at)

Review Timeline:

Submission Date:	14th Apr 25
Editorial Decision:	28th May 25
Appeal Received:	29th Jan 26
Editorial Decision:	18th Mar 26
Revision Received:	7th Apr 26
Accepted:	11th May 26

Editor: Cornelius Schneider

Transaction Report:

(Note: With the exception of the correction of typographical or spelling errors that could be a source of ambiguity, letters and reports are not edited. Depending on transfer agreements, referee reports obtained elsewhere may or may not be included in this compilation. Referee reports are anonymous unless the Referee chooses to sign their reports.)

1st Editorial Decision **28th May 2025**

Dear Prof. Ameres,

Thank you for submitting your manuscript for consideration by the EMBO Journal.

Your manuscript was sent to three referees and we have now received the comments of two of them. The third reviewer has not yet returned his/her report but the feedback by the other two referees together does not support further consideration by The EMBO Journal. Referee #1 voices concerns regarding the suitability of the experimental strategy employed in this manuscript which put most of the major findings into question. Referee #2 on the other hand while being more positive regarding the technical quality of the study does not find the overall conceptual advance striking.

Given these negative opinions and the fact that the EMBO Journal can only afford to accept papers which receive enthusiastic support from a majority of referees, we see little choice other to return the manuscript to you with the message that we cannot offer to publish it. I am sorry to have to disappoint you.

Yours sincerely,

Cornelius Schneider, PhD
Editor
The EMBO Journal
c.schneider@embojournal.org

Referee #1:

In this manuscript Navalayeu et al. analyze the impact of transient or constitutive depletion of specific subunits of the mouse exosome in ESC cells on the levels of the different subunits of this assembly, cell proliferation/viability and/or on exosome activities. The authors conclude their study by proposing an assembly pathway for the mammalian exosome and by demonstrating that the EXOSC1 subunit is dispensable for survival and exosome activity in mESC cells.

Technically, the data presented in this manuscript are overall of good quality and convincing. Yet, some experiments raise issues. For example, Figure 3 is unclear has one has to guess the meaning of I and E (I= Input? Immunoprecipitated?, E= Eluate? Other?). If "E" labelled samples correspond indeed precipitated proteins, tagged EXOSC2 fails to immunoprecipitate EXOSC1, EXOSC10 and DIS3. This suggests that the tag impairs the assembly of the fusion protein in complete exosome and questions the significance of the results obtained with this fusion. Further, it is unclear why the DIS3L signal is barely detectable in "I" fractions and so strong in "E" fractions.

However, the key issue with this manuscript is whether the data really support a defined exosome assembly pathway for the mammalian exosome. Data are collected hours, and up to 3 days, after initiating the depletion of specific subunits but assembly of the complex is likely to be much faster in vivo (in the order of minutes?). The authors observe accumulation of some subunits and partial complexes in the assay conditions and integrate the states observed after depletion of different subunits to propose a unique exosome assembly pathway. Yet, another interpretation of these data is that the exosome assembles through multiple pathways, but that upon long term depletion of subunits only some specific entities subsist, for example because they are stabilized by a specific cellular location and/or binding to some cellular factor (chaperones). Further, they may represent stable dead-end products. This possibility is particularly worrisome because cell proliferation is starting to slow-down and signs of apoptosis start to be detected: aberrant/dead assemblies are not uncommon in such stress conditions. At best, the study suggests an assembly pathway for mammalian exosome but this would need to be strengthened by showing that the partial assemblies accumulating are bona fide intermediates, e.g., that they are found in normal cells and can be chased in complete functional exosomes. More importantly, if the authors want to argue that there is a single/major pathway for exosome assembly in mammalian cells, they should provide evidence that alternative pathways do not play a significant role in exosome assembly, e.g., by showing that only the proposed intermediates are present in normal cells and/or contribute to exosome formation.

By testing PROMPT degradation and ribosomal RNA processing, authors also show that assembly of complete (or near complete) exosome is required for their in vivo activities. This is not unexpected given previously published work in this area. More importantly, they demonstrate that EXOSC1 is not required for the viability of mESC cells. This is an interesting observation.

Overall, the manuscript present solid data but those only partly support the main conclusion of the authors. More generally, the strategy of long-term depletion (with CRISPR or AID) reported here may help to understand complex assembly but has significant limitations. It is not suitable as a unique method and should be combined with independent evidence (biochemical

assays, chase experiments, pulse-chase, gradients, size fractionations...) to delineate solid assembly pathways. The current manuscript doe not seem suitable for publication in The EMBO Journal: it could be significantly enriched to support the interpretations proposed or might be submitted to a more specialized journal.

Other points:
- Introduction, page 3, line 6: It is unclear why authors only quote studies targeting human complexes while working with murine factors: earlier pioneering reports on eukaryotic complexes could be quoted.
- Page 5, line 8: "gRNA activity" is probably "predicted gRNA activity"? This also applies to discussion where this sentence is duplicated.
- Page 8 bottom, the discussion about the PIN domain is confusing given that it is found in both DIS3L and DIS3.
- Page 9, line 9: what is the evidence for "orphan protein decay"?
- Page 10, line 28 "repression" is confusing as it probably means degradation that is mechanistically different. The use of "repression" and "downregulation" and similar terms should be clarified throughout the manuscript.
- Studies of the exosome using native mass spectrometry has provided evidence for the stability of sub-assemblies. These data could be discussed here. Similarly, the molecular roles of the different (essential) subunits could be discussed briefly.
- Legend of Figure 2A: ** could not be fond.
- Figure 2B: the column sgOlfr1 should be deleted as Olfr1 was used for normalization. It is meaningless.

Referee #2:

Despite a large number of structural analyses, often involving reconstitution, the in vivo assembly pathway of the exosome complex has never been systematically addressed. Here the authors follow dependencies in exosome assembly, largely using protein degradation as a proxy for failure of proteins to be stably incorporated. The detailed analyses appear to have been well performed and the conclusion on the assembly order are logically developed. This order is not as might have anticipated, giving some novelty to the work. The finding that EXOSC1 is partially dispensable in ESCs is also of interest.

Overall, the paper reports a lot of careful analysis, and the conclusions appear sound. The general interest of the findings is moderate, and the paper format might have been more appropriate for a report style journal. However, I would be willing to support publication in EMBO J.

Minor points:

1) In the complexes that assemble in the absence of specific factors, is it clear that the components all remain monomeric? The intact complex has rotational pseudosymmetry, and it seems conceivable that duplicate copies of homologous components might be incorporated in the absence of the "correct" protein. This possibility could at least be mentioned in the text.

2) Line 25: " ..Western blot analyses of every RNA exosome subunits....."

** As a service to authors, EMBO Press provides authors with the possibility to transfer a manuscript that one journal cannot offer to publish to another EMBO publication or the open access journal Life Science Alliance launched in partnership between EMBO Press, Rockefeller University Press and Cold Spring Harbor Laboratory Press. The full manuscript and if applicable, reviewers' reports, are automatically sent to the receiving journal to allow for fast handling and a prompt decision on your manuscript. For more details of this service, and to transfer your manuscript please click on Link Not Available. **

Please do not share this URL as it will give anyone who clicks it access to your account.

Response to Reviewers

Manuscript: **Hierarchical assembly and functional resilience of the mammalian RNA exosome**
Original Manuscript ID: EMBOJ-2025-121077-T

We thank the editor and the reviewers for their careful evaluation of our manuscript and for the constructive feedback. Below we provide a detailed point-by-point response.

Reviewer #1:

In this manuscript Navalayeu et al. analyze the impact of transient or constitutive depletion of specific subunits of the mouse exosome in ESC cells on the levels of the different subunits of this assembly, cell proliferation/viability and/or on exosome activities. The authors conclude their study by proposing an assembly pathway for the mammalian exosome and by demonstrating that the EXOSC1 subunit is dispensable for survival and exosome activity in mESC cells.
Technically, the data presented in this manuscript are overall of good quality and convincing. Yet, some experiments raise issues. For example, Figure 3 is unclear has one has to guess the meaning of I and E (I= Input? Immunoprecipitated?, E= Eluate? Other?).

We thank the referee for pointing this out. We agree that the abbreviation “E” is potentially misleading in this context, as the immunoprecipitated material was not eluted from the beads but instead corresponds to the bead-bound fraction that was directly resolved by SDS–PAGE. To avoid ambiguity and to reflect the experimental procedure more accurately, we have revised the nomenclature throughout the manuscript. The bound fraction is now consistently referred to as “B” (bead-bound) or “IP” (for αV5-IP / α SPOT IP), while “I” denotes the input. Figure labels and legends have been updated accordingly throughout the manuscript, and this clarification has also been added to the Methods section to explicitly describe how bound material was processed and analyzed.

If "E" labelled samples correspond indeed precipitated proteins, tagged EXOSC2 fails to immunoprecipitate EXOSC1, EXOSC10 and DIS3. This suggests that the tag impairs the assembly of the fusion protein in complete exosome and questions the significance of the results obtained with this fusion. Further, it is unclear why the DIS3L signal is barely detectable in "I" fractions and so strong in "E" fractions.”

Under control conditions, Exosc1 and Exosc10 are reproducibly recovered in Exosc2 immunoprecipitates as detected by western blot or mass-spectrometry (Figure 3A; Figure S2G and S2I), indicating that tagged Exosc2 is stably incorporated into RNA exosome complexes. Importantly, expression of tagged Exosc2 functionally complements endogenous Exosc2 depletion without affecting cell viability (Figure S2F and S2K), arguing against tag-induced assembly defects. Immunoprecipitation is used here as a qualitative readout of stable interactions rather than as a quantitative measure of subunit stoichiometry. Consequently, reduced recovery of specific subunits upon depletion reflects genuine changes in complex composition rather than impaired assembly caused by the tag.

Regarding DIS3 and DIS3L, DIS3 is known to associate only weakly and transiently with the RNA exosome core, whereas DIS3L shows stronger association. Consistent with this, DIS3 displays limited co-migration with the core complex in our size-exclusion chromatography (SEC) analysis (Figure S2J and S2L), in agreement with recent reports (Latini et al., Cell Reports 2024). DIS3L enrichment in eluates reflects this stronger association rather than a technical artifact.

To further validate these interactions, we performed additional IP and western blot analyses using Exosc2-HA rescue cell line (see Figure R1) which independently confirm stable interactions between Exosc2 and core exosome subunits as well as the expected behavior of catalytic subunits.

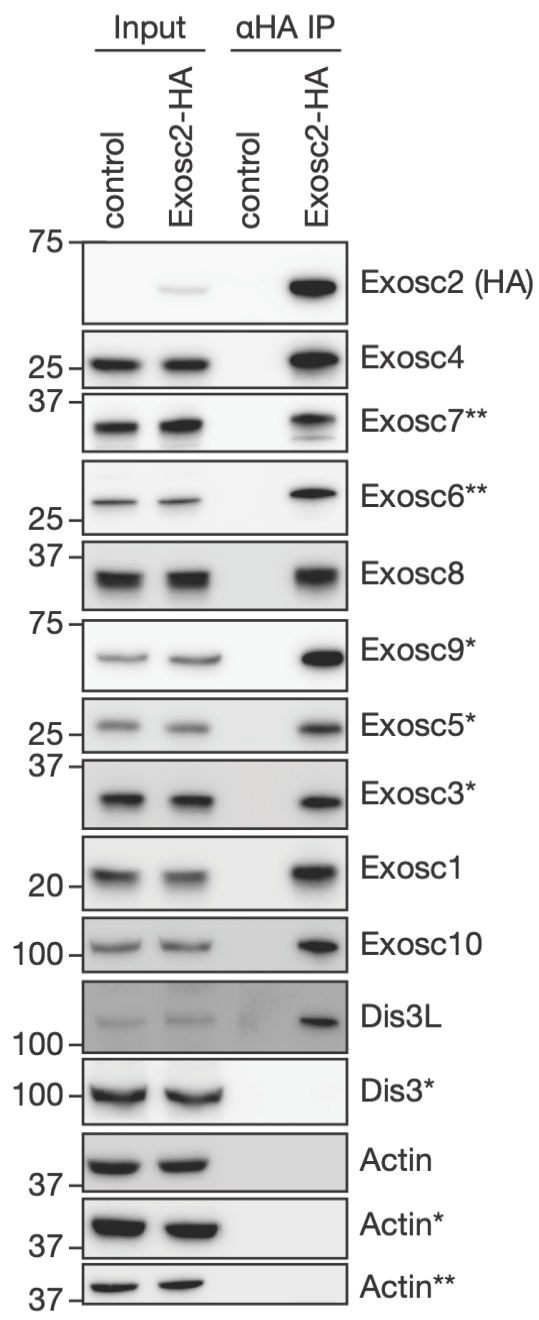

Figure R1. RNA exosome subunit interactions in an Exosc2-HA rescue cell line.
Whole-cell lysates from control and Exosc2-HA rescue mESCs were subjected to anti-HA immunoprecipitation. Input (I) and bead-bound fractions (αHA IP) were analyzed by SDS–PAGE and western blotting for the indicated RNA exosome core and catalytic subunits. Like other core exosome components (Exosc3, Exosc4, Exosc5, Exosc6, Exosc7, Exosc8, and Exosc9), Exosc1 and Exosc10 are reproducibly detected in Exosc2-HA immunoprecipitates, indicating stable incorporation of tagged Exosc2 into RNA exosome complexes. DIS3L shows robust enrichment in the bound fraction, in contrast to Dis3, consistent with its transient association with the exosome core. Asterisks denote independent analysis of identical samples on separate gels and membranes to avoid stripping artifacts. Actin serves as a loading control.

However, the key issue with this manuscript is whether the data really support a defined exosome assembly pathway for the mammalian exosome. Data are collected hours, and up to 3 days, after initiating the depletion of specific subunits but assembly of the complex is likely to be much faster in vivo (in the order of minutes?). The authors observe accumulation of some subunits and partial complexes in the assay conditions and integrate the states observed after depletion of different subunits to propose a unique exosome assembly pathway. Yet, another interpretation of these data is that the exosome assembles through multiple pathways, but that upon long term depletion of subunits only some specific entities subsist, for example because they are stabilized by a specific cellular location and/or binding to some cellular factor (chaperones). Further, they may represent stable dead-end products. This possibility is particularly worrisome because cell proliferation is starting to slow-down and signs of apoptosis start to be detected: aberrant/dead assemblies are not uncommon in such stress conditions. At best, the study suggests an assembly pathway for mammalian exosome but this would need to be strengthened by showing that the partial assemblies accumulating are bona fide intermediates, e.g., that they are found in normal cells and can be chased in complete functional exosomes.

We fully agree that distinguishing bona fide assembly intermediates from stress-induced or dead-end products is critical. Addressing this concern was the primary focus of our revision, and we therefore added multiple independent and complementary experimental approaches that go beyond long-term depletion.

First, we combined three orthogonal depletion systems (iCas9, AID, and dTAG) to examine RNA exosome assembly across distinct temporal windows, including early timepoints (2–48 h) well before detectable proliferation defects or apoptotic responses (Figure 2C-D, S1I-T and S2A-D). Across all systems and timepoints, depletion of individual subunits reproducibly yielded the same partial assemblies, arguing against stress-induced artifacts or late secondary effects.

Second, to determine whether the observed assemblies exist under physiological conditions, we performed size-exclusion chromatography (SEC) on lysates from unperturbed wild-type mESCs (Figure S2J). SEC revealed co-fractionation of RNA exosome core subunits in high–molecular weight fractions and a relative depletion of late-stage assembly components, including Exosc5, Exosc3, and Exosc1, in lighter fractions. As co-fractionation alone does not establish that these subunits reside within the same molecular complex, we next combined SEC with quantitative immunoprecipitation–mass spectrometry using a functional Exosc2-HA rescue line (Figure 3I and S2K–L). This analysis demonstrated near-stoichiometric representation of all core subunits in heavy fractions (A7–A10) and a progressive, ordered loss of late-assembling components in lighter fractions (B3–B6 and B8–B11), consistent with hierarchical assembly rather than heterogeneous aggregation.

Finally, to directly address the request for chase-type evidence, we implemented a time-resolved incorporation assay using doxycycline-inducible Exosc7-3xFLAG expression (Figure 3J and S2M). Newly synthesized Exosc7 initially associates with early-stage subunits and is subsequently incorporated into fully assembled RNA exosome complexes within defined time windows, demonstrating that early assemblies mature into complete complexes rather than accumulating as dead-end products. Please note, that timepoints earlier than 1 hour could not be reliably resolved due to very low basal leakiness of the inducible system, which convolutes detection of nascent assembly complexes at earlier stages.

Together, these independent biochemical and kinetic experiments strongly support our model that the observed partial assemblies are bona fide intermediates in mammalian RNA exosome biogenesis.

"More importantly, if the authors want to argue that there is a single/major pathway for exosome assembly in mammalian cells, they should provide evidence that alternative pathways do not play a significant role in exosome assembly, e.g., by showing that only the proposed intermediates are present in normal cells and/or contribute to exosome formation."

We have revised the manuscript to clarify that our conclusions refer to a predominant and hierarchical sequence of inter-subunit dependencies rather than a single exclusive assembly pathway. While our data support a dominant assembly trajectory, we do not exclude the existence of alternative or parallel routes. This clarification and an expanded discussion of pathway flexibility have been added to the revised Discussion.

By testing PROMPT degradation and ribosomal RNA processing, authors also show that assembly of complete (or near complete) exosome is required for their in vivo activities. This is not unexpected given previously published work in this area.
More importantly, they demonstrate that EXOSC1 is not required for the viability of mESC cells. This is an interesting observation.

We thank the referee for highlighting these points. While complete or near-complete RNA exosome assembly is required for many functions, systematic analyses of individual subunit contributions remain limited. Our study provides a comprehensive, subunit-resolved analysis and demonstrates that Exosc1 is dispensable for mESC viability, revealing functional resilience and modularity of the RNA exosome in this cellular context. This observation is now emphasized more clearly in the revised manuscript.

Overall, the manuscript present solid data but those only partly support the main conclusion of the authors. More generally, the strategy of long-term depletion (with CRISPR or AID) reported here may help to understand complex assembly but has significant limitations. It is not suitable as a unique method and should be combined with independent evidence (biochemical assays, chase experiments, pulse-chase, gradients, size fractionations...) to delineate solid assembly pathways.

We agree that long-term depletion alone has limitations and should not be used as a unique approach. In the revised study, long-term depletion is no longer used in isolation. Instead, we integrate early depletion using orthogonal systems, size-based biochemical fractionation, quantitative proteomics, and time-resolved incorporation assays, as detailed in the response above. The convergence of these independent approaches directly addresses the apparent limitations.

Other points:
Introduction, page 3, line 6: It is unclear why authors only quote studies targeting human complexes while working with murine factors: earlier pioneering reports on eukaryotic complexes could be quoted.
Page 5, line 8: "gRNA activity" is probably "predicted gRNA activity"? This also applies to discussion where this sentence is duplicated.
Page 8 bottom, the discussion about the PIN domain is confusing given that it is found in both DIS3L and DIS3.
Page 9, line 9: what is the evidence for "orphan protein decay"?
Page 10, line 28 "repression" is confusing as it probably means degradation that is mechanistically different. The use of "repression" and "downregulation" and similar terms should be clarified throughout the manuscript.
Studies of the exosome using native mass spectrometry has provided evidence for the stability of sub-assemblies. These data could be discussed here. Similarly, the molecular roles of the different (essential) subunits could be discussed briefly.
*Legend of Figure 2A: ** could not be fond.*
Figure 2B: the column sgOlfr1 should be deleted as Olfr1 was used for normalization. It is meaningless."

All points listed above have been addressed in the revised manuscript. Specifically, we added references to early eukaryotic exosome studies, clarified terminology and duplicated text, revised the discussion of PIN domains, provided evidence and clarification for orphan protein decay, clarified the use of terms such as repression and downregulation, expanded the discussion to include native mass spectrometry studies and subunit roles, corrected the Figure 2A legend, and removed the sgOlfr1 column from Figure 2B.

Reviewer #2:

Despite a large number of structural analyses, often involving reconstitution, the in vivo assembly pathway of the exosome complex has never been systematically addressed. Here the authors follow dependencies in exosome assembly, largely using protein degradation as a proxy for failure of proteins to be stably incorporated. The detailed analyses appear to have been well performed and the conclusion on the assembly order are logically developed. This order is not as might have anticipated, giving some novelty to the work. The finding that EXOSC1 is partially dispensable in ESCs is also of interest.
Overall, the paper reports a lot of careful analysis, and the conclusions appear sound. The general interest of the findings is moderate, and the paper format might have been more appropriate for a report style journal. However, I would be willing to support publication in EMBO J.

We thank the referee for the careful evaluation of our work and for the positive assessment of the analyses, conclusions, and novelty. We appreciate the support for publication in EMBO Journal.

Minor points
1) In the complexes that assemble in the absence of specific factors, is it clear that the components all remain monomeric? The intact complex has rotational pseudosymmetry, and it seems conceivable that duplicate copies of homologous components might be incorporated in the absence of the "correct" protein. This possibility could at least be mentioned in the text.

We agree that this is an important conceptual consideration. Our SEC and quantitative IP–MS analyses in the revised manuscript (Figure 3I, S2J–L) support the presence of defined RNA exosome assemblies with compositions consistent with known stoichiometries and structural constraints of the complex. In particular, we observe selective and ordered loss of late-assembling subunits rather than increased abundance of homologous components, arguing against stable incorporation of duplicate subunits. While we cannot formally exclude the possibility of transient associations, our data do not provide evidence for such assemblies, nor do they affect the interpretation of the proposed assembly dependencies.

2) Line 25: " ..Western blot analyses of every RNA exosome subunits....."

This has been corrected in the revised manuscript.

Dear Dr Ameres,

Thank you for submitting a revised version of your manuscript. Your study has now been seen by one of the original referees, who finds that their previous concerns have been addressed and now recommends publication of the manuscript pending minor revisions. There remain only a few mainly editorial points that have to be addressed before I can extend formal acceptance of the manuscript. The figure check for this manuscript is unusually complex, as it involves the analysis of over 600 sub-images. We will therefore contact you separately once this process has been completed:

- MANUSCRIPT FORMAT: Please provide the manuscript in Word format

- Please make sure that there is only one Data Availability Section titled "Data Availability" (Lead Contact, Materials availability, etc. should be removed)

- Please rename the Conflict of Interest section into "Disclosure and Competing Interests Statement", in accordance with our updated Guide to Authors (https://link.springer.com/partners/embo-press/editorial-policies#Competing%20interest%20disclosures)

- AFFILIATIONS: Please remove the affiliations from the footnotes on the title page, as we need them on the title page

- As we are switching from a free-text author contribution statement towards a more formal statement based on Contributor Role Taxonomy (CRediT) terms, please remove the present Author Contribution section and instead specify each author's contribution(s) directly in the Author Information page of our submission system during upload of the final manuscript. See https://casrai.org/credit/ for more information.

- Please adjust the format of the reference list and of the in-text citations according to EMBO Journal format (alphabetical order, author name et al + year.../up to 10 author names in the reference list before et al / please refer to our Guide to Authors for additional information on EMBO J reference format).

- Please provide either a "Yes" or a "Not Applicable" answer to each one of the questions in your Author Checklist (https://media.springernature.com/original/springer-cms/rest/v1/content/27825796/data/v1). In the last column of this checklist, only the sections of the manuscript where the relevant information can be found should be listed (the information per se should be included in the main manuscript file).

- FUNDING INFO: Boehringer Ingelheim Fonds PhD fellowship needs to be removed from the Comments box and provided as a separate funding entry via More Funders option; Austrian Academy of Sciences is missing from the system as a separate funder

- Please provide Figure S4 (EV4) on a single page.

- FIGURE CALLOUTS: Figure 1E-H called out but Figure 1 only has A-E panels

- DATASET EV LEGENDS: 7 EV tables provided, but the nomenclature and file names need to be updated in all places: Table S1 should be renamed to Table EV1, Table S2 should be renamed to Table EV2, Table EV3 is a dataset and needs to be updated to Dataset EV1, Table EV4 - EV7 cannot be opened (if these are also complex Excel tables, then we need them updated to datasets - Dataset EV2, etc.)

- Please provide suggestions for a short 'blurb' text prefacing and summing up the conceptual aspect of the study in two sentences (max. 250 characters), followed by 3-5 one-sentence 'bullet points' with brief factual statements of key results of the paper; they will form the basis of an editor-written 'Synopsis' accompanying the online version of the article. Please also provide an altered synopsis image, making sure that the aspect ratio conforms to our website's format - it should be exactly 550 pixels wide and between 300-600 pixels high.

- R&T TABLE: in the ms - needs to be removed and upldd separately

- The manuscript sections should be in the following order: Title page - Abstract & Keywords - Introduction - Results - Discussion - Methods - Data Availability - Acknowledgments - Disclosure Statement & Competing Interests - References - Figure Legends - (Main Tables with legends if applicable) - Expanded View Figure Legends.

- Summary should be renamed to Abstract

- EV figures have wrong nomenclature, which should be corrected in all places to Figure EV1, Figure EV2, etc. (NOT Figure S1, etc)

- Please note that the specific URL for PXD061800 dataset is not provided in the data availability statement.

- Figure legends:
1. Please note that information related to n is missing in the legends of figures 3I, S4 F

2. Please note that n=2 in figures S3 A, B

3. Please note that the error bars are not defined in the legends of figures 2F, 3I; 4G, S4 F

- Figure Legends (main + EV):
1. Please note that the exact p values are not provided in the legends of figures 2F, 4B, G;S3 A, B; S4 K, L

2. Please indicate the statistical test used for data analysis in the legends of figures S2 G, L; S4Q

Please let me know if you have any questions regarding any of these points. You can use the link below to upload the revised files.
Thank you again for giving us the chance to consider your manuscript for The EMBO Journal. I look forward to receiving the final version.
With best regards,
Cornelius Schneider

____
Cornelius Schneider, PhD
Editor | The EMBO Journal
c.schneider@embojournal.org

Please refer to our figure preparation guideline in order to ensure proper formatting and readability in print as well as on screen:

https://link.springer.com/journal/44318/submission-guidelines#cms-Figure-and-data-presentation

Use the link below to submit your revision:

https://emboj.msubmit.net/cgi-bin/main.plex

Referee #1:

In this revised version of their manuscript, the authors provide additional evidence for the exosome assembly pathway they deduce from their experiments. This includes for example alternative strategies to deplete exosome subunits, or cell fractionation combined with mass spectrometry to identify putative assembly intermediates. Further, the authors clarify that the proposed pathway may only be one of multiple assembly pathway. Overall, these new data strengthen the manuscript.
Some points remain weak. For example, there is no real chase experiment demonstrating that complexes accumulating following the depletion of a subunit are not dead-end products. Also, the authors' answer to the low level of DIS3, Exosc1 and Exosc10 found co-precipitating with Exosc2 is not entirely convincing. (i) For example, structures of human exosome containing DIS3 have solved without apparent issues for the stable incorporation of this subunit these entities. (ii) In their answer, the authors argue that "Immunoprecipitation is used here as a qualitative readout of stable interactions rather than as a quantitative measure of subunit stoichiometry. Consequently, reduced recovery of specific subunits upon depletion reflects genuine changes in complex composition rather than impaired assembly caused by the tag.". This argument is not convincing: the authors argue that the assay is not quantitative but qualitative to conclude that a "reduced recovery" (a quantitative assessment) "reflects genuine changes"!
Overall, the manuscript has been strengthened and I believe that a revised manuscript answering the main point listed above and the detailed comments below may be suitable for publication in the EMBO Journal.

Detailed comments:
- Title: "Hierarchical assembly" still suggests that there is a single assembly pathway. This should probably be adjusted. Further, "Hierarchical assembly" was previously demonstrated in vitro. The authors provide evidence for a stepwise assembly in vivo: this new point should be clear in the title.
- While outside of the scope of the current manuscript, it would be of interest to know the kinetic of exosome assembly in vivo.

The authors might want to comment on this point as data reported in Figure 3J argue set a maximum value of 90 min for this process. SILAC labelling could have provided a more precise estimation.
- Interaction between complex subunits have been shown in some case to initiate as early as co-translationally (e.g. work from the Bukau lab). Are they evidence of this mechanism for the exosome worth mentioning? This general point could be presented in the introduction.
- Page 6, line 6: mentions Figure 1 E-H but there is no Figure 1 F-H.

Referee #1 (Report for Author)

In this revised version of their manuscript, the authors provide additional evidence for the exosome assembly pathway they deduce from their experiments. This includes for example alternative strategies to deplete exosome subunits, or cell fractionation combined with mass spectrometry to identify putative assembly intermediates. Further, the authors clarify that the proposed pathway may only be one of multiple assembly pathway. Overall, these new data strengthen the manuscript.

We thank the referee for the positve evaluation of our work and for recognizing the value of the additional experiments and clarifications introduced in this revision.

Some points remain weak. For example, there is no real chase experiment demonstrating that complexes accumulating following the depletion of a subunit are not dead-end products.

We agree that a classical pulse-chase experiment demonstrating that RNA exosome sub-complexes accumulating upon subunit depletion represent productive assembly intermediates rather than dead-end products would be highly informative. Such an approach is, however, technically challenging for a multi-subunit complex in mammalian cells. To address this question, we employed a complementary time-resolved incorporation assay based on doxycycline-inducible expression of Exosc7-3xFLAG (Figure 3J and EV2M). This analysis shows that newly synthesized Exosc7 initially associates with early assembly subunits, and only subsequently appears in higher-order complexes, consistent with progressive incorporation into the RNA exosome rather than accumulation in dead-end states. Importantly, these observations are supported by independent approaches, including size-exclusion chromatography (Figure 3I) and perturbation analyses, which together provide convergent evidence for a productive assembly trajectory. To avoid overinterpretation, we have revised the manuscript to clarify the level of evidence supporting this conclusion.

Also, the authors' answer to the low level of DIS3, Exosc1 and Exosc10 found co-precipitating with Exosc2 is not entirely convincing. (i) For example, structures of human exosome containing DIS3 have solved without apparent issues for the stable incorporation of this subunit these entities. (ii) In their answer, the authors argue that "Immunoprecipitation is used here as a qualitative readout of stable interactions rather than as a quantitative measure of subunit stoichiometry. Consequently, reduced recovery of specific subunits upon depletion reflects genuine changes in complex composition rather than impaired assembly caused by the tag.". This argument is not convincing: the authors argue that the assay is not quantitative but qualitative to conclude that a "reduced recovery" (a quantitative assessment) "reflects genuine changes"!

We thank the referee for pointing out the ambiguity in our previous wording. We agree that our initial description was imprecise. Immunoprecipitation coupled to western blotting analysis in our study provides a semi-quantitative measure of subunit co-enrichment rather than a strictly qualitative or fully quantitative assessment of stoichiometry. To ensure that the observed differences in subunit recovery reflect changes in complex composition rather than tag-specific effects, we performed orthogonal

experiments using multiple tagged Exosc2 variants (V5, HA, SPOT; Figure EV2G-I and EV2L). In addition, we carried out inducible depletion of early- and late-assembling subunits followed by HA-immunoprecipitation and quantitative mass spectrometry (Figure R1). Across all approaches, we observe consistent changes in subunit association patterns, supporting the interpretation that these reflect bona fide differences in assembly state rather than artefacts of the tagging strategy. We have revised the manuscript to clarify this point.

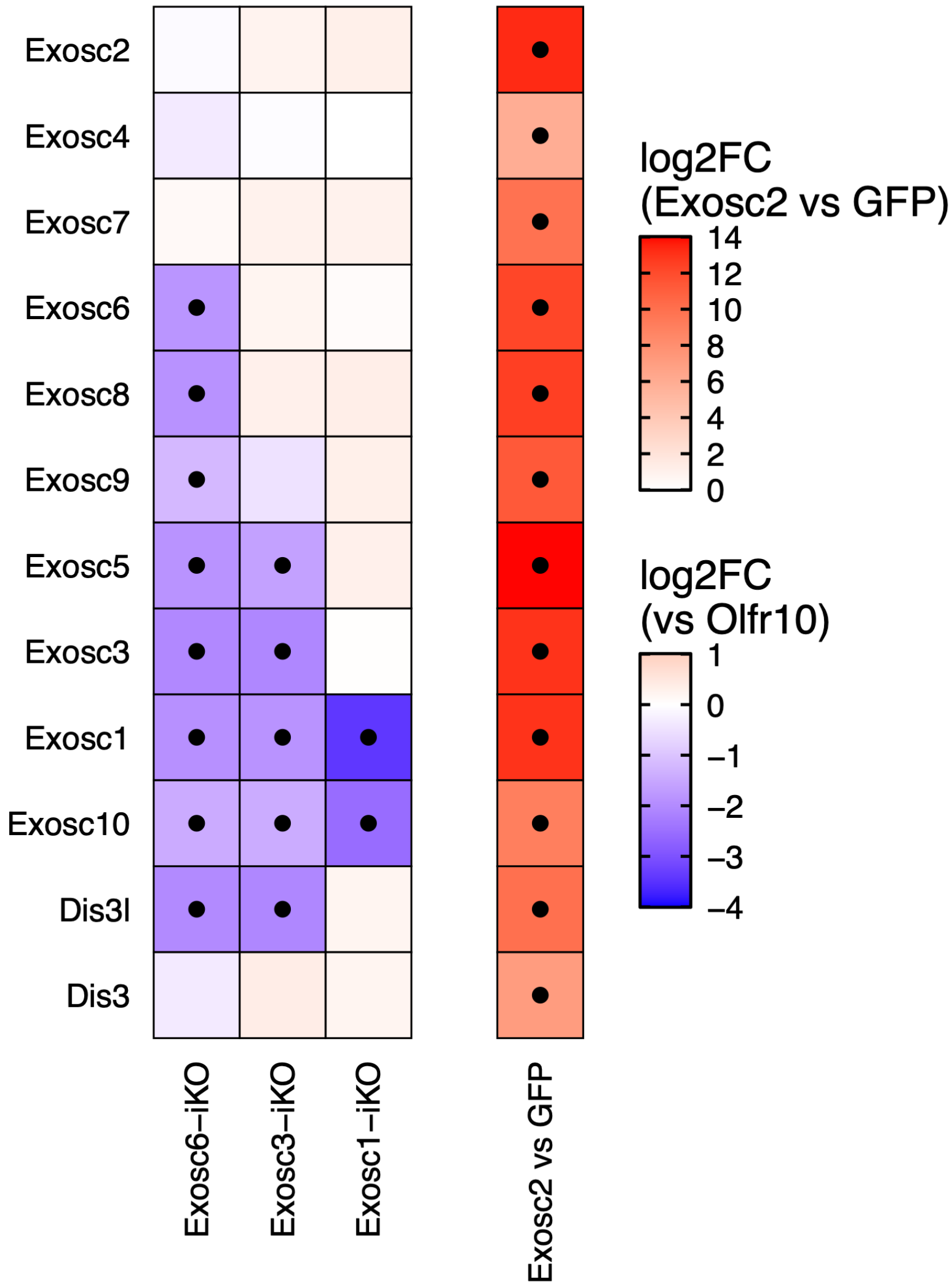

Figure R1. Composition of RNA exosome subcomplexes in Exosc6-iKO, Exosc3-iKO and Exosc1-iKO cells.
(Left) Whole cell lysates from GFP-HA or Exosc2-HA mESCs were subjected to anti-HA immunoprecipitation followed by mass-spectrometry analysis. Enrichment of the RNA exosome subunits in Exosc2-HA over the GFP-HA control is indicated on the heatmap. Black dots indicate instances with log2FC >1 and $p_{adj} < 0.05$.
(Right) Whole-cell lysates of Exosc2-HA mESCs with inducible depletion of Exosc6, Exosc3, Exosc1 and Olfr10 were subjected to anti-HA immunoprecipitation and mass-spectrometry analysis. Enrichment of the RNA exosome subunits in the respective depletions over the Olfr10-iKO control is shown on the heatmap. Black dots indicate instances with log2FC < -1 and $p_{adj} < 0.05$.

Regarding Dis3, several previous studies have reported weak or condition-dependent association of Dis3 with the RNA exosome core in mammalian cells (Chen et al., 2001; Staals et al., 2010; Tomecki et al., 2010). More recent fractionation analyses further indicate that a substantial fraction of Dis3 is present outside of core RNA exosome complexes (Latini et al., 2024; Xiao Tao et al., 2025). Consistent with these observations, we detect only modest enrichment of Dis3 in our immunoprecipitation experiments performed at 150 mM salt. Notably, earlier work showed that Dis3

association is highly sensitive to buffer conditions, with stronger recovery observed at reduced ionic strength. Thus, the limited recovery of Dis3 in our experiments is in line with established biochemical properties and does not contradict its incorporation in fully assembled complexes.

Overall, the manuscript has been strengthened and I believe that a revised manuscript answering the main point listed above and the detailed comments below mays be suitable for publication in the EMBO Journal.

We thank the referre for the careful evaluation of our work and for supporting its publication in The EMBO Journal.

Detailed comments:

- Title: "Hierarchical assembly" still suggests that there is a single assembly pathway. This should probably be adjusted. Further, "Hierarchical assembly" was previously demonstrated in vitro. The authors provide evidence for a stepwise assembly in vivo: this new point should be clear in the title.

We agree that the original title may suggest a single obligatory assembly pathway and does not sufficiently emphasize the cellular context of our study. We have therefore revised the title to: “Cellular assembly and functional resilience of the mammalian RNA exosome” This revised title avoids implying a single assembly route and better reflects both the cellular setting and the functional insights of our study.

- While outside of the scope of the current manuscript, it would be of interest to know the kinetic of exosome assembly in vivo. The authors might want to comment on this point as data reported in Figure 3J argue set a maximum value of 90 min for this process. SILAC labelling could have provided a more precise estimation.

We agree that determining the kinetics of RNA exosome assembly in vivo would be highly informative. Our time-resolved incorporation assay (Figure 3J) provides an upper bound and suggests that assembly is largely completed within approximately 90 minutes under our experimental conditions. We agree that approaches such as SILAC-based pulse labeling could provide more precise kinetic measurements. However, such analyses would require substantial additional experimental work and were therefore beyond the scope of the present study.

- Interaction between complex subunits have been shown in some case to initiate as early as co-translationally (e.g. work from the Bukau lab). Are they evidence of this mechanism for the exosome worth mentioning? This general point could be presented in the introduction.

We agree that co-translational assembly is an important mechanism for many multiprotein complexes. However, to our knowledge no direct evidence currently exists for co- or post-translational assembly of the RNA exosome, which is why we refrain from discussing this aspect in depth in this manuscript.

- Page 6, line 6: mentions Figure 1 E-H but there is no Figure 1 F-H.

We thank the referee for noting this error. The figure reference has been corrected to Figure EV1E-H in the revised manuscript.

Dear Prof. Ameres,

I am pleased to inform you that your manuscript has been accepted for publication in the EMBO Journal.

Your manuscript will be processed for publication by EMBO Press. It will be copy edited and you will receive page proofs prior to publication. Please note that you will be contacted by Springer Nature Author Services to complete licensing and payment information.

You may qualify for financial assistance for your publication charges - either via a Springer Nature fully open access agreement or an EMBO initiative. Check your eligibility: https://link.springer.com/journal/44318/how-to-publish-with-us

Should you be planning a Press Release on your article, please get in contact with embo_production@springernature.com as early as possible in order to coordinate publication and release dates.

If you have any questions, please do not hesitate to contact the Editorial Office. Thank you for your contribution to The EMBO Journal.

Yours sincerely,

Cornelius Schneider, PhD
Editor
The EMBO Journal
c.schneider@embojournal.org

*****
Please note that it is The EMBO Journal policy for the transcript of the editorial process (containing referee reports and your response letters) to be published as an online supplement to each paper. If you should prefer removal of any referee-only figures included in the point-by-point response(s), e.g. because they may still be used for future publication or because they have been reproduced from published work by others, please do let us know immediately via response email.
More information is available here: https://link.springer.com/partners/embo-press/editorial-policies#Peer%20review

** Click here to be directed to your login page: https://emboj.msubmit.net

EMBO Press Author Checklist

Corresponding Author Name: Stefan L. Ameres
Journal Submitted to: The EMBO Journal
Manuscript Number: EMBOJ-2025-121077R-Q

USEFUL LINKS FOR COMPLETING THIS FORM
The EMBO Journal - Author Guidelines
EMBO Reports - Author Guidelines
Molecular Systems Biology - Author Guidelines
EMBO Molecular Medicine - Author Guidelines

Reporting Checklist for Life Science Articles (updated January
This checklist is adapted from Materials Design Analysis Reporting (MDAR) Checklist for Authors. MDAR establishes a minimum set of requirements in transparent reporting in the life sciences (see Statement of Task: 10.31222/osf.io/9sm4x). Please follow the journal's guidelines in preparing your
Please note that a copy of this checklist will be published alongside your article.

Abridged guidelines for figures
1. Data
The data shown in figures should satisfy the following conditions:
- the data were obtained and processed according to the field's best practice and are presented to reflect the results of the experiments in an accurate and unbiased manner.
- ideally, figure panels should include only measurements that are directly comparable to each other and obtained with the same assay.
- plots include clearly labeled error bars for independent experiments and sample sizes. Unless justified, error bars should not be shown for technical
- if n<5, the individual data points from each experiment should be plotted. Any statistical test employed should be justified.
- Source Data should be included to report the data underlying figures according to the guidelines set out in the authorship guidelines on Data

2. Captions
Each figure caption should contain the following information, for each panel where they are relevant:
- a specification of the experimental system investigated (eg cell line, species name).
- the assay(s) and method(s) used to carry out the reported observations and measurements.
- an explicit mention of the biological and chemical entity(ies) that are being measured.
- an explicit mention of the biological and chemical entity(ies) that are altered/varied/perturbed in a controlled manner.
- the exact sample size (n) for each experimental group/condition, given as a number, not a range;
- a description of the sample collection allowing the reader to understand whether the samples represent technical or biological replicates (including how many animals, litters, cultures, etc.).
- a statement of how many times the experiment shown was independently replicated in the laboratory.
- definitions of statistical methods and measures:
  - common tests, such as t-test (please specify whether paired vs. unpaired), simple χ2 tests, Wilcoxon and Mann-Whitney tests, can be unambiguously identified by name only, but more complex techniques should be described in the methods section;
  - are tests one-sided or two-sided?
  - are there adjustments for multiple comparisons?
  - exact statistical test results, e.g., P values = x but not P values < x;
  - definition of 'center values' as median or average;
  - definition of error bars as s.d. or s.e.m.

Please complete ALL of the questions below.
Select "Not Applicable" only when the requested information is not relevant for your study.

Materials

Newly Created Materials	Information included in the manuscript?	In which section is the information available? (Reagents and Tools Table, Materials and Methods, Figures, Data Availability Section)
New materials and reagents need to be available; do any restrictions apply?	Not Applicable	

Antibodies	Information included in the manuscript?	In which section is the information available? (Reagents and Tools Table, Materials and Methods, Figures, Data Availability Section)
For **antibodies** provide the following information:<br>- Commercial antibodies: RRID (if possible) or supplier name, catalogue number and or/clone number<br>- Non-commercial: RRID or citation	Yes	Reagents and Tools Table, Materials and Methods

DNA and RNA sequences	Information included in the manuscript?	In which section is the information available? (Reagents and Tools Table, Materials and Methods, Figures, Data Availability Section)
**Short novel DNA or RNA including primers, probes**: provide the sequences.	Yes	Materials and Methods, Table EV1 and Table EV2

Cell materials	Information included in the manuscript?	In which section is the information available? (Reagents and Tools Table, Materials and Methods, Figures, Data Availability Section)
**Cell lines:** Provide species information, strain. Provide accession number in repository **OR** supplier name, catalog number, clone number, and/**OR** RRID.	Yes	Reagents and Tools Table, Materials and Methods
**Primary cultures:** Provide species, strain, sex of origin, genetic modification status.	Not Applicable	
Report if the cell lines were recently **authenticated** (e.g., by STR profiling) and tested for mycoplasma contamination.	Yes	The cells are not authenticated. The cells are regularly tested for mycoplasma contamination

Experimental animals	Information included in the manuscript?	In which section is the information available? (Reagents and Tools Table, Materials and Methods, Figures, Data Availability Section)
**Laboratory animals or Model organisms:** Provide species, strain, sex, age, genetic modification status. Provide accession number in repository **OR** supplier name, catalog number, clone number, **OR** RRID.	Not Applicable	
**Animal observed in or captured from the field:** Provide species, sex, and age where possible.	Not Applicable	
Please detail **housing and husbandry conditions**.	Not Applicable	

Plants and microbes	Information included in the manuscript?	In which section is the information available? (Reagents and Tools Table, Materials and Methods, Figures, Data Availability Section)
**Plants:** provide species and strain, ecotype and cultivar where relevant, unique accession number if available, and source (including location for collected wild specimens).	Not Applicable	
**Microbes:** provide species and strain, unique accession number if available, and source.	Not Applicable	

Human research participants	Information included in the manuscript?	In which section is the information available? (Reagents and Tools Table, Materials and Methods, Figures, Data Availability Section)
If collected and within the bounds of privacy constraints report on age, sex and gender or ethnicity for all study participants.	Not Applicable	

Core facilities	Information included in the manuscript?	In which section is the information available? (Reagents and Tools Table, Materials and Methods, Figures, Data Availability Section)
If your work benefited from core facilities, was their service mentioned in the acknowledgments section?	Yes	Acknowledgments

Design

Study protocol	Information included in the manuscript?	In which section is the information available? (Reagents and Tools Table, Materials and Methods, Figures, Data Availability Section)
If study protocol has been **pre-registered, provide DOI in the manuscript**. For clinical trials, provide the trial registration number **OR** cite DOI.	Not Applicable	
Report the **clinical trial registration number** (at ClinicalTrials.gov or equivalent), where applicable.	Not Applicable	

Laboratory protocol	Information included in the manuscript?	In which section is the information available? (Reagents and Tools Table, Materials and Methods, Figures, Data Availability Section)
Provide DOI OR other citation details if **external detailed step-by-step protocols** are available.	Not Applicable	

Experimental study design and statistics	Information included in the manuscript?	In which section is the information available? (Reagents and Tools Table, Materials and Methods, Figures, Data Availability Section)
Include a statement about **sample size** estimate even if no statistical methods were used.	Yes	Figure legends
Were any steps taken to minimize the effects of subjective bias when allocating animals/samples to treatment (e.g. **randomization procedure**)? If yes, have they been described?	Not Applicable	
Include a statement about **blinding** even if no blinding was done.	Not Applicable	
Describe **inclusion/exclusion criteria** if samples or animals were excluded from the analysis. Were the criteria pre-established?<br><br>If sample or data points were omitted from analysis, report if this was due to attrition or intentional exclusion and provide justification.	Yes	Materials and Methods
For every figure, are **statistical tests** justified as appropriate? Do the data meet the assumptions of the tests (e.g., normal distribution)? Describe any methods used to assess it. Is there an estimate of variation within each group of data? Is the variance similar between the groups that are being statistically compared?	Yes	Figure legends

Sample definition and in-laboratory replication	Information included in the manuscript?	In which section is the information available? (Reagents and Tools Table, Materials and Methods, Figures, Data Availability Section)
In the figure legends: state number of times the experiment was **replicated** in laboratory.	Yes	Figure legends
In the figure legends: define whether data describe **technical or biological replicates**.	Yes	Figure legends

Ethics

Ethics	Information included in the manuscript?	In which section is the information available? (Reagents and Tools Table, Materials and Methods, Figures, Data Availability Section)
Studies involving **human participants**: State details of **authority granting ethics approval** (IRB or equivalent committee(s), provide reference number for approval.	Not Applicable	
Studies involving **human participants**: Include a statement confirming that **informed consent** was obtained from all subjects and that the experiments conformed to the principles set out in the WMA Declaration of Helsinki and the Department of Health and Human Services Belmont Report.	Not Applicable	
Studies involving **human participants:** For publication of **patient photos**, include a statement confirming that consent to publish was obtained.	Not Applicable	
Studies involving experimental **animals**: State details of **authority granting ethics approval** (IRB or equivalent committee(s), provide reference number for approval. Include a statement of compliance with ethical regulations.	Not Applicable	
Studies involving **specimen and field samples:** State if relevant **permits** obtained, provide details of authority approving study; if none were required, explain why.	Not Applicable	

Dual Use Research of Concern (DURC)	Information included in the manuscript?	In which section is the information available? (Reagents and Tools Table, Materials and Methods, Figures, Data Availability Section)
Could your study fall under dual use research restrictions? Please check biosecurity documents and list of **select agents and toxins** (CDC): https://www.selectagents.gov/sat/list.htm	Not Applicable	
If you used a select agent, is the security level of the lab appropriate and reported in the manuscript?	Not Applicable	
If a study is subject to dual use research of concern regulations, is the name of the **authority granting approval and reference number** for the regulatory approval provided in the manuscript?	Not Applicable	

Reporting

The MDAR framework recommends adoption of discipline-specific guidelines, established and endorsed through community initiatives. Journals have their own policy about requiring specific guidelines and recommendations to complement MDAR.

Adherence to community standards	Information included in the manuscript?	In which section is the information available? (Reagents and Tools Table, Materials and Methods, Figures, Data Availability Section)
State if relevant guidelines or checklists (e.g., **ICMJE, MIBBI, ARRIVE, PRISMA**) have been followed or provided.	Not Applicable	
For **tumor marker prognostic studies,** we recommend that you follow the **REMARK** reporting guidelines (see link list at top right). See author guidelines, under 'Reporting Guidelines'. Please confirm you have followed these guidelines.	Not Applicable	
For **phase II and III randomized controlled trials**, please refer to the **CONSORT** flow diagram (see link list at top right) and submit the CONSORT checklist (see link list at top right) with your submission. See author guidelines, under 'Reporting Guidelines'. Please confirm you have submitted this list.	Not Applicable	

Data Availability

Data availability	Information included in the manuscript?	In which section is the information available? (Reagents and Tools Table, Materials and Methods, Figures, Data Availability Section)
Have **primary datasets** been deposited according to the journal's guidelines (see 'Data Deposition' section) and the respective accession numbers provided in the Data Availability Section?	Yes	Data availability
Were **human clinical and genomic datasets** deposited in a public access-controlled repository in accordance to ethical obligations to the patients and to the applicable consent agreement?	Not Applicable	
Are **computational models** that are central and integral to a study available without restrictions in a machine-readable form? Were the relevant accession numbers or links provided?	Not Applicable	
If publicly available data were reused, provide the respective **data citations in the reference list**.	Not Applicable	
