# Supplementary material for: Cellular assembly and functional resilience of the mammalian RNA exosome
Source: EMBO J. 2026 Jun 25;45(15):5423–56. doi: 10.1038/s44318-026-00824-x (PMC13434269; doi:10.1038/s44318-026-00824-x)
Supplement: Supplementary file 2 [file 44318_2026_824_MOESM9_ESM.pdf]

# Expanded View Figures

**Figure EV1. Establishment, validation, and benchmarking of a dual-guide iCas9 system to interrogate proteostatic dependencies among RNA exosome components, related to Figs. 1 and 2.**

(**A**) Schematic of the generation of clonal iCas9 cell lines. Roman numbers indicate vectors used for the engineering of the first (iCas9v1) and second (iCas9v2) versions of iCas9. For iCas9v1, expression cassettes for rtTA3 and Cas9 were packaged in lentiviruses and sequentially integrated into the genome. For iCas9v2, the "all-in-one" cassette was site-specifically integrated in the expression-stable locus on Chr15 (see Materials and Methods). (**B**) (Left): Flow cytometry evaluation of inducible Cas9 expression in iCas9v1 and iCas9v2 in the presence or absence of Dox. (Right): Evaluation of the editing efficiency of endogenously expressed YFP (iCas9v1) or surface marker CD9 (iCas9v2) in iCas9 cells transduced with the respective sgRNAs. For CD9 analysis, cells were immunostained before flow cytometry measurements. (**C**) iCas9 cells expressing different pairs of dual sgRNAs against Exosc1 (pair1–4) were treated with Dox for 0, 24, 48, and 72 h. Total proteins were analysed with Western blot against Exosc1 and Hsp70. Dilution series (5, 10, 25, 50, 100%) were used to estimate the sensitivity of the antibody, and Hsp70 served as a loading control. (**D**) Competitive proliferation assays of iCas9 cells harboring dual sgRNAs against Exosc1 (pair1–4) and Olfr10 (control). The percentage of sgRNA+ cells was monitored with flow cytometry. Values were normalized to Day0 and are represented as mean ± sd ($n = 2$ technical replicates). (**E**) Evaluation of the depletion efficiency of AID-tagged proteins upon IAA administration in AID-Exosc3 (top) and Exosc6-AID (bottom) cells. Tir1 (control) is a parental cell line that served as a negative control. Total proteins were analysed with Western blot using the indicated antibody. Dilution series (10, 25, 50, 100%) were used to estimate the sensitivity of the antibody, and actin represents a loading control. (**F**) Competitive proliferation assay of AID-Exosc3 (top) and Exosc6-AID (bottom) in the presence and absence of IAA. The percentage of AID-positive cells was monitored with flow cytometry. Values were normalized to Day0 and are represented as mean ± sd ($n = 2$ biological replicates). (**G**) Evaluation of the depletion efficiency of dTAG-tagged proteins upon dTAG administration in Exosc7-dTAG, Exosc8-dTAG and Exosc9-dTAG cells. Parental iCas9v2 cell line (control) served as a negative control. Total proteins were analysed with Western blot using the indicated antibody. Dilution series (10, 25, 50, and 100%) were used to estimate the sensitivity of the antibody, and actin represents a loading control. (**H**) Competitive proliferation assay of Exosc7-dTAG, Exosc8-dTAG, and Exosc9-dTAG cells in the presence and absence of dTAG. The percentage of dTAG-positive cells was monitored with flow cytometry. Values were normalized to Day0 and are represented as mean ± sd ($n = 3$ biological replicates). (**I–T**) iCas9 cells harboring dual sgRNAs against Exosc2 (**I**), Exosc4 (**J**), Exosc7 (**K**), Exosc6 (**L**), Exosc8 (**M**), Exosc9 (**N**), Exosc5 (**O**), Exosc3 (**P**), Exosc1 (**Q**), Exosc10 (**R**), Dis3L (**S**), and Dis3 (**T**) were treated with Dox for 0, 24, 48, and 72 h. Total proteins were analyzed with a Western blot for the indicated RNA exosome components. Dilution series (10, 25, 50, and 100%) were used to estimate the sensitivity of the antibody, and Hsp70 served as a loading control. Black asterisk (*) refers to the corresponding gel and purple asterisk (*) indicates a nonspecific band.

 © The Author(s)

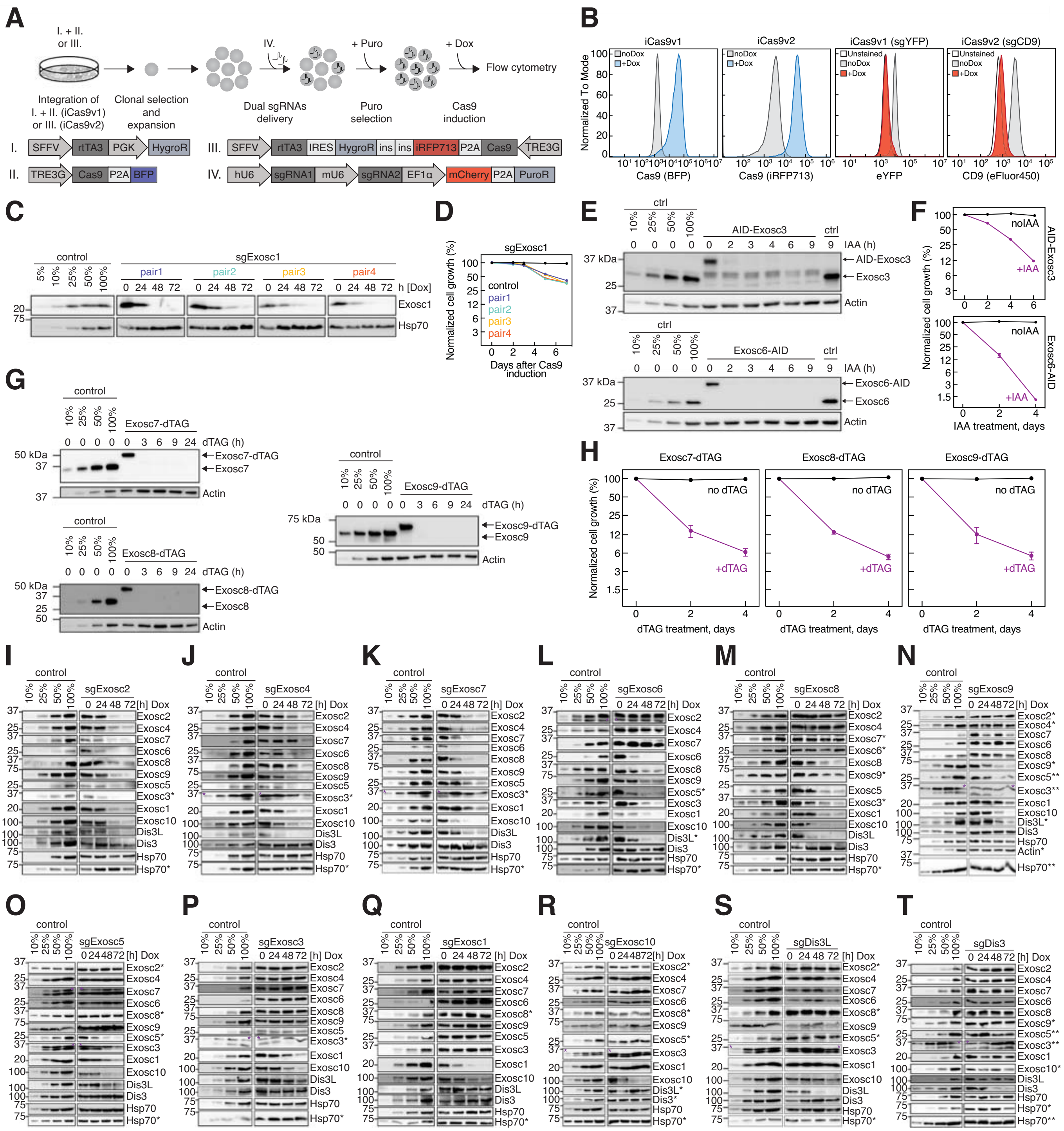

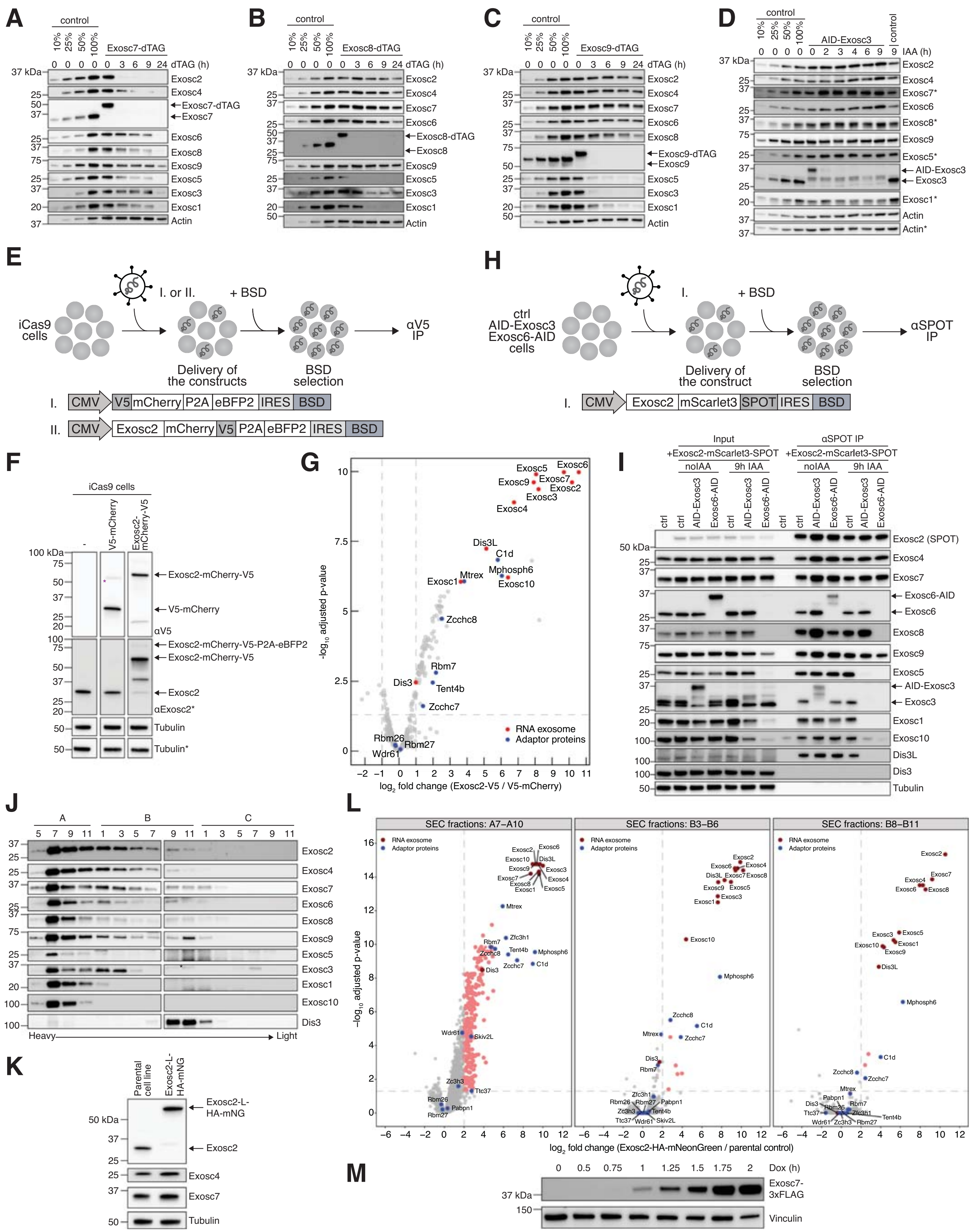
A
control
Exosc7-dTAG
dTAG (h)
Exosc2
Exosc4
Exosc7-dTAG
Exosc7
Exosc6
Exosc8
Exosc9
Exosc5
Exosc3
Exosc1
Actin
B
Exosc8-dTAG
Exosc8
C
Exosc9-dTAG
Exosc9
D
AID-Exosc3
IAA (h)
E
iCas9 cells
I. or II.
+ BSD
Delivery of the constructs
BSD selection
αV5 IP
I. CMV V5 mCherry P2A eBFP2 IRES BSD
II. CMV Exosc2 mCherry V5 P2A eBFP2 IRES BSD
H
ctrl AID-Exosc3 Exosc6-AID cells
Delivery of the construct
αSPOT IP
I. CMV Exosc2 mScarlet3 SPOT IRES BSD
F
iCas9 cells
V5-mCherry
Exosc2-mCherry-V5
Exosc2-mCherry-V5-P2A-eBFP2
αExosc2*
Tubulin
G
-log10 adjusted p-value
log2 fold change (Exosc2-V5 / V5-mCherry)
RNA exosome
Adaptor proteins
I
Input
+Exosc2-mScarlet3-SPOT
noIAA
9h IAA
αSPOT IP
Exosc2 (SPOT)
Exosc6-AID
Exosc10
Dis3L
Dis3
J
Heavy
Light
K
Parental cell line
Exosc2-L-HA-mNG
Exosc2-L-HA-mNG
L
SEC fractions: A7–A10
SEC fractions: B3–B6
SEC fractions: B8–B11
log2 fold change (Exosc2-HA-mNeonGreen / parental control)
M
Dox (h)
Exosc7-3xFLAG
Vinculin


© The Author(s)

**Figure EV2. Rapid depletion of RNA exosome subunits and interrogation of RNA exosome assembly intermediates, related to Figs. 2 and 3.**

(**A–C**) Exosc7-dTAG (**A**), Exosc8-dTAG (**B**), and Exosc9-dTAG (**C**) cells were treated with dTAG for the indicated period pf time. Parental iCas9v2 cell line (control) served as a negative control. Total proteins were analyzed with Western blot using the indicated antibody. Dilution series (10, 25, 50, and 100%) were used to estimate the sensitivity of the antibody, and Actin served as a loading control. (**D**) Parental Tir1 (ctrl) and AID-Exosc3 cells were treated with IAA for the indicated period of time. Total proteins were analyzed with Western blot using the indicated antibody. Dilution series (10, 25, 50, and 100%) were used to estimate the sensitivity of the antibody, and Actin served as a loading control. Asterisk (*) refers to the corresponding gel. (**E**) Control V5-mCherry-P2A-eBFP2 or Exosc2-mCherry-V5-P2A-eBFP2 cassettes were lentivirally delivered to iCas9 cells, followed by Blasticidin S (BSD) selection. (**F**) Western blot analysis of the cell lines from (**A**) with αV5 and αExosc2 antibody. Tubulin served as a loading control. Black asterisk (*) refers to the corresponding gel. Purple asterisk (*) indicates the signal from V5-mCherry-P2A-eBFP2. (**G**) Mass-spectrometry analysis of αV5-immunoprecipitation from Exosc2-mCherry-V5 or V5-mCherry control samples. The $\log_2$ fold change (x axis) is plotted against the $-$log10 of Benjamini–Hochberg adjusted limma-moderated two-sided *t*-test *p* value (y axis; $p_{adj}$). Dashed lines indicate the thresholds of $p_{adj} < 0.05$ and $\log_2 FC < -1$ or $>1$ ($n = 3$ biological replicates). (**H**) Generation of ectopic expression of Exosc2-mScarlet3-SPOT in the parental Tir1 (ctrl), AID-Exosc3, and Exosc6-AID cell lines. The cassette was virally delivered, and cells were selected with Blasticidin S (BSD). (**I**) αSPOT-IP of cells from (**H**). Input and bead-bound proteins were analyzed with a Western blot using the indicated antibody. Tubulin served as a loading control. (**J**) Total protein extract of wild-type mESCs was subjected to size-exclusion chromatography followed by western blot analysis using the indicated antibodies. (**K**) Generation and validation of Exosc2-HA-mNeonGreen (labeled as Exosc2-L-HA-mNG) knockout-rescue clonal cell line. Total protein was analyzed with a Western blot using the indicated antibodies. (**L**) Total protein extract of Exosc2-HA-mNeonGreen and untagged parental control was subjected to size-exclusion chromatography, and indicated fractions were pooled, followed by αHA-immunoprecipitation and mass-spectrometry analysis. The $\log_2$ fold change (x axis) is plotted against the $-$log10 of Benjamini–Hochberg adjusted limma-moderated two-sided *t*-test *p* value (y axis; $p_{adj}$). Dashed lines indicate the threshold of $p_{adj} < 0.05$ and $\log_2 FC > 2$ ($n = 3$ biological replicates). (**M**) Cells harboring doxycycline-inducible Exosc7-3xFLAG cassette were subjected to doxycycline (Dox) treatment for the indicated period of time, followed by western blot analysis. Vinculin served as a loading control.

© The Author(s) 

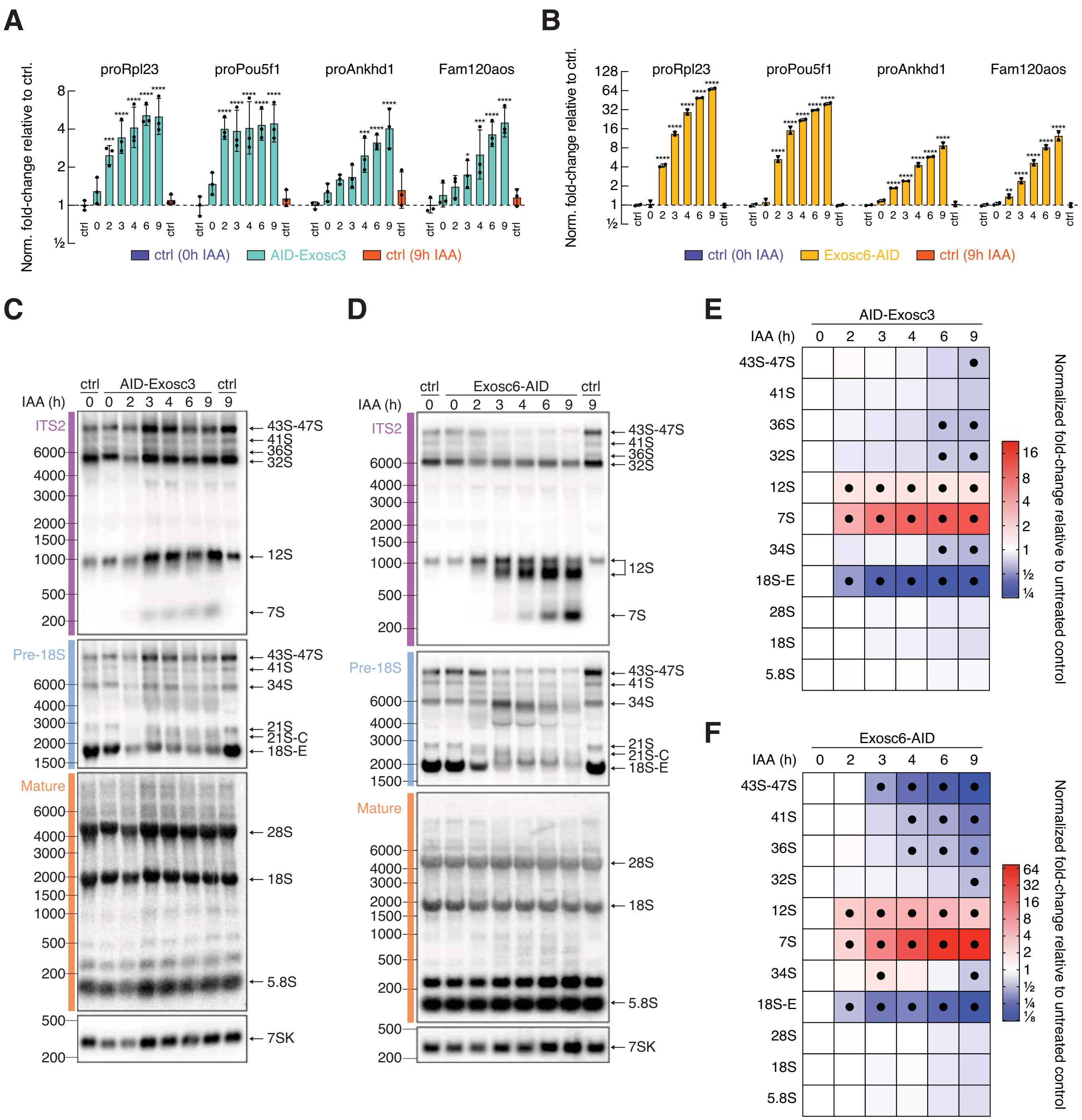
A
B
C
D
E
F
proRpl23
proPou5f1
proAnkhd1
Fam120aos
Norm. fold-change relative to ctrl.
ctrl (0h IAA)
AID-Exosc3
ctrl (9h IAA)
Exosc6-AID
IAA (h)
ITS2
Pre-18S
Mature
43S-47S
41S
36S
32S
12S
7S
34S
21S
21S-C
18S-E
28S
18S
5.8S
7SK
Normalized fold-change relative to untreated control

◀ **Figure EV3. Stabilization of PROMPTs and accumulation of rRNA processing defects upon depletion of Exosc3 and Exosc6 by AID, related to Fig. 4.**

(**A**, **B**) Parental Tir1 (ctrl), AID-Exosc3 (**A**) and Exosc6-AID (**B**) cells were treated with IAA for the indicated time period. Total RNA was isolated, and PROMPTs abundance was measured with RT-qPCR. Values were normalized to Gapdh mRNA, and mean ± sd is shown relative to ctrl 0 h IAA ($n = 3$ biological replicates for AID-Exosc3 (**A**) and $n = 2$ biological replicates for AID-Exosc6 (**B**)). Statistical significance of the difference between a given sample and "0 h IAA ctrl" was determined with a two-way ANOVA test and Holm–Šídák multiple comparison correction ($^{*}p_{adj} < 0.05$, $^{**}p_{adj} < 0.0021$, $^{***}p_{adj} < 0.0002$, $^{****}p_{adj} < 0.0001$, non-significant $p_{adj} > 0.05$ is not marked). Exact adjusted *p* values: (**A**) AID-Exosc3 vs control (0 h IAA): proRpl23: $1.38 \times 10^{-4}$, $5.4 \times 10^{-7}$, $2.23 \times 10^{-8}$, $4.34 \times 10^{-10}$, $5.89 \times 10^{-10}$; proPou5f1: $2.44 \times 10^{-8}$, $3.92 \times 10^{-8}$, $2.33 \times 10^{-8}$, $9.57 \times 10^{-9}$, $6.55 \times 10^{-9}$; proAnkhd1: $2.53 \times 10^{-4}$, $4.94 \times 10^{-6}$, $4.35 \times 10^{-8}$; Fam120aos: $3.38 \times 10^{-2}$, $1.85 \times 10^{-4}$, $2.80 \times 10^{-7}$, $5.28 \times 10^{-9}$; (**B**) Exosc6-AID vs control (0 h IAA): proRpl23: $<1.0 \times 10^{-15}$, $<1.0 \times 10^{-15}$, $<1.0 \times 10^{-15}$, $<1.0 \times 10^{-15}$, $<1.0 \times 10^{-15}$; proPou5f1: $<1.0 \times 10^{-15}$, $<1.0 \times 10^{-15}$, $<1.0 \times 10^{-15}$, $<1.0 \times 10^{-15}$, $<1.0 \times 10^{-15}$; proAnkhd1: $8.75 \times 10^{-8}$, $4.42 \times 10^{-11}$, $<1.0 \times 10^{-15}$, $<1.0 \times 10^{-15}$, $<1.0 \times 10^{-15}$; Fam120aos: $1.95 \times 10^{-3}$, $3.64 \times 10^{-11}$, $<1.0 \times 10^{-15}$, $<1.0 \times 10^{-15}$, $<1.0 \times 10^{-15}$. (**C**,**D**) Parental Tir1 (ctrl), AID-Exosc3 (**C**) and Exosc6-AID (**D**) cells were treated with IAA for the indicated time period. Total RNA was resolved on the agarose gel, followed by northern blot analysis. (**E**,**F**) Heatmap of northern blot quantifications for AID-Exosc3 (**E**) and Exosc6-AID (**F**). Abundance of rRNA processing intermediates was normalized to 7SK RNA, and the mean is shown relative to untreated AID-Exosc3 or Exosc6-AID samples ($n = 3$ for AID-Exosc3 and $n = 2$ for Exosc6-AID biological replicates). Statistical significance of the difference between a given sample and untreated AID-Exosc3 or Exosc6-AID controls was determined with a two-way ANOVA test and Holm–Šídák multiple comparison correction. Black dots indicate instances with $p_{adj} < 0.05$.

© The Author(s)

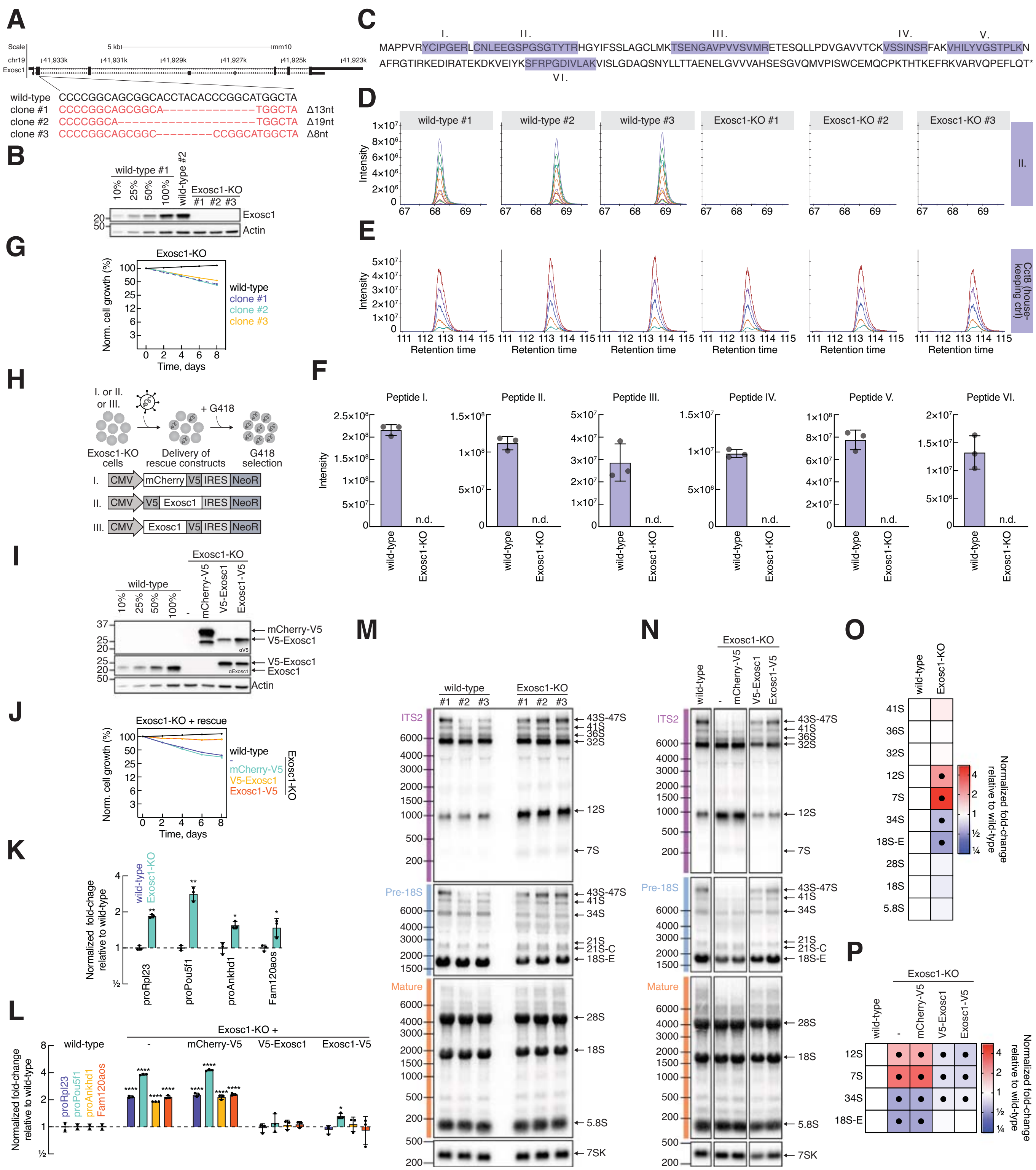
A
wild-type CCCCGGCAGCGGCACCTACACCCGGCATGGCTA
clone #1 CCCCGGCAGCGGCA--------------TGGCTA Δ13nt
clone #2 CCCCGGCA--------------------TGGCTA Δ19nt
clone #3 CCCCGGCAGCGGC--------CCGGCATGGCTA Δ8nt
B
wild-type #1
10% 25% 50% 100%
wild-type #2
Exosc1-KO
#1 #2 #3
Exosc1
Actin
C
MAPPVRYCIPGERLCNLEEGSPGSGTYTRHGYIFSSLAGCLMKTSENGAVPVVSVMRETESQLLPDVGAVVTCKVSSINSRFAKVHILYVGSTPLKN
AFRGTIRKEDIRATEKDKVEIYKSFRPGDIVLAKVISLGDAQSNYLLTTAENELGVVVAHSESGVQMVPISWCEMQCPKTHTKEFRKVARVQPEFLQT*
D
wild-type #1
wild-type #2
wild-type #3
Exosc1-KO #1
Exosc1-KO #2
Exosc1-KO #3
Intensity
E
Cct8 (house-keeping ctrl)
Retention time
F
Peptide I.
Peptide II.
Peptide III.
Peptide IV.
Peptide V.
Peptide VI.
Intensity
n.d.
wild-type
Exosc1-KO
G
Exosc1-KO
Norm. cell growth (%)
Time, days
wild-type
clone #1
clone #2
clone #3
H
I. or II. or III.
+ G418
Exosc1-KO cells
Delivery of rescue constructs
G418 selection
I. CMV mCherry V5 IRES NeoR
II. CMV V5 Exosc1 IRES NeoR
III. CMV Exosc1 V5 IRES NeoR
I
wild-type
Exosc1-KO
mCherry-V5
V5-Exosc1
Exosc1-V5
mCherry-V5
V5-Exosc1
Exosc1
Actin
J
Exosc1-KO + rescue
Norm. cell growth (%)
Time, days
wild-type
-
mCherry-V5
V5-Exosc1
Exosc1-V5
Exosc1-KO
K
Normalized fold-chage relative to wild-type
wild-type
Exosc1-KO
proRpl23
proPou5f1
proAnkhd1
Fam120aos
L
Normalized fold-change relative to wild-type
wild-type
Exosc1-KO +
-
mCherry-V5
V5-Exosc1
Exosc1-V5
proRpl23
proPou5f1
proAnkhd1
Fam120aos
M
wild-type
Exosc1-KO
#1 #2 #3
ITS2
Pre-18S
Mature
43S-47S
41S
36S
32S
12S
7S
34S
21S
21S-C
18S-E
28S
18S
5.8S
7SK
N
wild-type
Exosc1-KO
-
mCherry-V5
V5-Exosc1
Exosc1-V5
O
wild-type
Exosc1-KO
41S
36S
32S
12S
7S
34S
18S-E
28S
18S
5.8S
Normalized fold-change relative to wild-type
P
Exosc1-KO
wild-type
-
mCherry-V5
V5-Exosc1
Exosc1-V5
12S
7S
34S
18S-E
Normalized fold-change relative to wild-type

 © The Author(s)

**Figure EV4. Establishment, validation and characterization of mESC lines depleted of Exosc1 by CRISPR/Cas9 genome engineering, related to Fig. 4.**

(**A**) Schematic of Exosc1 genomic loci. Frameshift mutations were introduced in the second coding exon with the CRISPR/Cas9 system. Genotypes of three Exosc1-KO clones used in this study are indicated. (**B**) Western blot analysis of wild-type and clonal Exosc1-KO cell lines. Dilution series (10, 25, 50, 100%) were used to estimate the sensitivity of the antibody, and actin represents a loading control. (**C**) Protein sequence of Exosc1. Six peptides that were measured with parallel reaction monitoring (PRM) mass-spectrometry are highlighted with blue rectangles and labeled with roman numbers (I-VI). (**D**) Example PRM analysis of wild-type and Exosc1-KO cells for the peptide II. PRM intensity signal (y-axis) is plotted against the retention time (x-axis). Multiple curves correspond to MS/MS = MS2 fragments. (**E**) Example PRM analysis of one of the peptides of the Cct8 protein that served as a "loading control". Data were represented as in (**D**). See Materials and methods for information about the reference proteins used in PRM experiments. (**F**) PRM signal intensity of Exosc1 peptides in wild-type and Exosc1-KO cells shown as mean ± sd. No signal was detected in Exosc1-KO samples and is depicted as n.d. (not determined; $n = 3$ biological replicates). (**G**) Competitive proliferation assay of wild-type and Exosc1-KO cells. The percentage of wild-type or Exosc1-KO cells was monitored with flow cytometry. Values were normalized to Day0 and are shown as mean ± sd ($n = 2$ technical replicates). (**H**) Schematic of Exosc1 rescue experiments. Rescue constructs harboring mCherry-V5 or V5-Exosc1 or Exosc1-V5 were virally delivered to Exosc1-KO cells, followed by G418 selection. (**I**) Western blot analysis of the cells with rescue constructs. Membranes were probed with αV5 and αExosc1 antibody to confirm transgene expression. Dilution series (10, 25, 50, and 100%) were used to estimate the sensitivity of the antibody, and actin served as a loading control. (**J**) Competitive proliferation assay, as in (**G**), for Exosc1 rescue cells. (**K**,**L**) Total RNA from wild-type, Exosc1-KO (**K**) and rescue (**L**) experiments was isolated, and PROMPTs abundance was measured with RT-qPCR. Values were normalized to Gapdh mRNA, and mean ± sd is shown relative to wild-type ($n = 3$ biological replicates). Statistical significance of the difference between a given sample and wild-type was determined with an unpaired *t*-test (for **K**) or two-way ANOVA (for **L**) and Holm–Šídák multiple comparison correction. ($n = 3$ biological replicates, (*$p_{adj} < 0.05$, **$p_{adj} < 0.0021$, ***$p_{adj} < 0.0002$, ****$p_{adj} < 0.0001$, non-significant $p_{adj} > 0.05$ is not marked). Exact adjusted *p* values: (**K**) WT vs Exosc1-KO: $2.0 \times 10^{-4}$, $7.0 \times 10^{-4}$, $7.9 \times 10^{-3}$, $2.2 \times 10^{-2}$; (**L**) WT vs Exosc1-KO: $3.38 \times 10^{-7}$, $4.42 \times 10^{-13}$, $8.73 \times 10^{-6}$, $3.73 \times 10^{-7}$; WT vs Exosc1-KO + mCherry-V5: $1.14 \times 10^{-7}$, $5.8 \times 10^{-14}$, $6.05 \times 10^{-7}$, $9.57 \times 10^{-8}$; WT vs Exosc1-KO + Exosc1-V5: $4.34 \times 10^{-2}$. (**M**, **N**) Total RNA from wild-type, Exosc1-KO (**M**) and rescue (**N**) experiments was resolved on the agarose gel, followed by northern blot analysis. (**O**,**P**) Heatmap of northern blot quantifications for wild-type, Exosc1-KO (**O**) and rescue (**P**) experiments. Values were normalized to 7SK RNA, and the mean is presented relative to wild-type ($n = 3$ biological replicates). Statistical significance of the difference between a given sample and wild-type was determined with an unpaired *t*-test (for **O**) or two-way ANOVA (for **P**) and Holm–Šídák multiple comparison correction ($n = 3$ biological replicates. Black dots indicate instances with $p_{adj} < 0.05$.

© The Author(s)

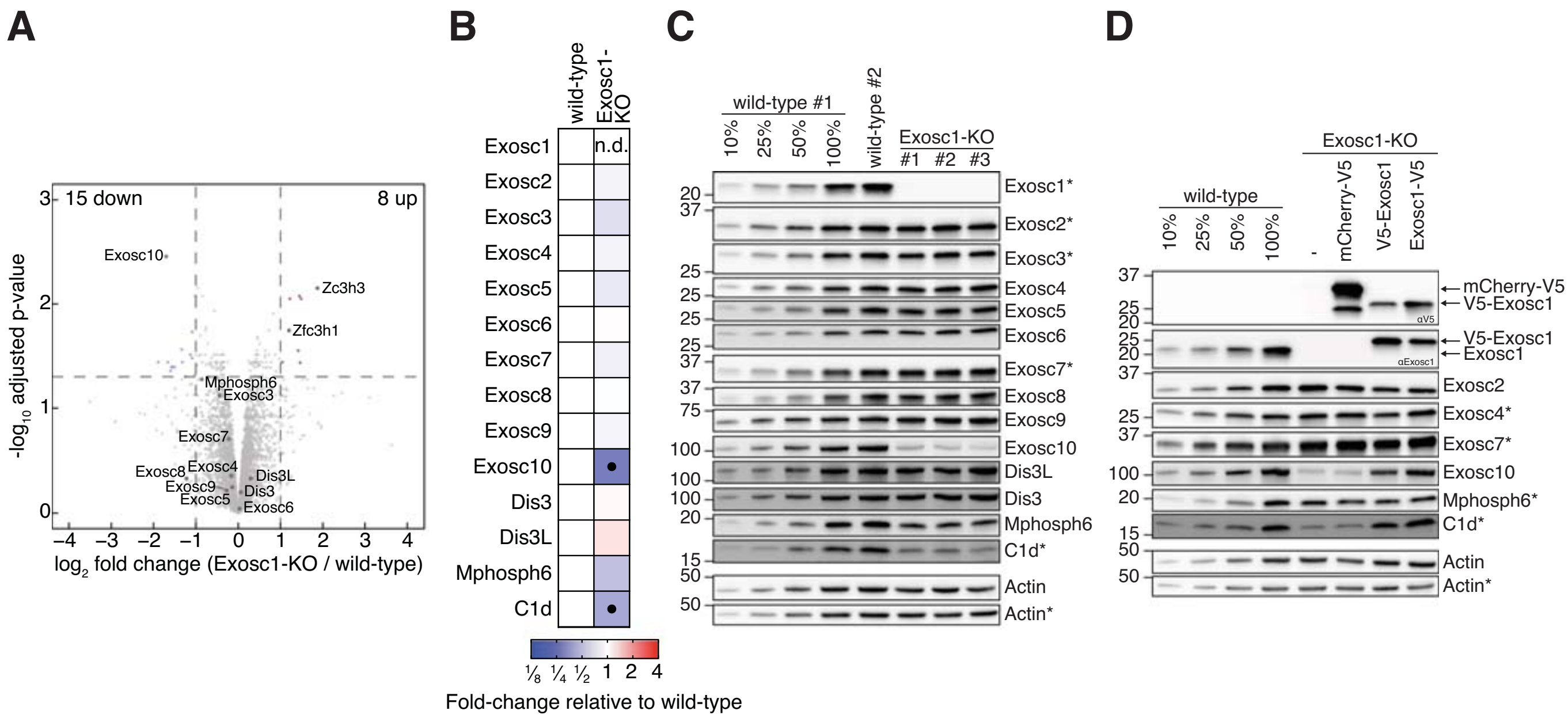


**Figure EV5. Proteomics characterization of clonal Exosc1-KO mESCs, related to Fig. 4.**

(**A**) Volcano plots displaying whole proteome changes for Exosc1-KO. The $\log_2$ fold change (x axis) is plotted against the −log10 of Benjamini–Hochberg adjusted limma-moderated two-sided *t*-test *p*-value (y axis; $p_{adj}$). Dashed lines indicate the thresholds of $p_{adj} < 0.05$ and $\log_2FC < -1$ or $>1$ ($n = 3$ biological replicates). (**B**) Relative protein abundance of RNA exosome subunits in Exosc1-KO determined by shotgun and PRM mass-spectrometry. Values are shown relative to wild-type sample. Statistical significance of the difference between Exosc1-KO and wild-type was determined with an unpaired *t*-test and Holm–Šídák multiple comparison correction ($n = 3$ biological replicates). Black dots indicate instances where $p_{adj} < 0.05$. No signal for Exosc1 peptides was measured in Exosc1-KO samples and is depicted as n.d. (not determined). (**C**, **D**) Western blot analysis of wild-type, Exosc1-KO (**C**) and rescue cells (**D**) with the indicated antibody. Dilution series (10, 25, 50, and 100%) were used to estimate the sensitivity of the antibody and Actin represents a loading control. Asterisk (*) refers to the corresponding gel.

 © The Author(s)
