# Supplementary material for: Cellular assembly and functional resilience of the mammalian RNA exosome
Source: EMBO J. 2026 Jun 25;45(15):5423–56. doi: 10.1038/s44318-026-00824-x (PMC13434269; doi:10.1038/s44318-026-00824-x)
Supplement: Supplementary file 3 [file 44318_2026_824_MOESM1_ESM.docx]

**Table EV1: Guide RNA oligonucleotides**

| **Gene** | **Oligo name** | **Oligo sequence** | **Application** |
| --- | --- | --- | --- |
| Exosc1 pair1 | Exosc1_66583_1st_gRNA_Fw | caccGATGTAGCCATGCCGGGTGT | dual gRNAs for inducible knockout of Exosc1 |
|  | Exosc1_66583_1st_gRNA_Rev | aaacACACCCGGCATGGCTACATC |  |
|  | Exosc1_66583_2nd_gRNA_Fw | cttgtttGGGGATGCAGTACCTCACgttta |  |
|  | Exosc1_66583_2nd_gRNA_Rev | ctcttaaacGTGAGGTACTGCATCCCCaaa |  |
| Exosc1 pair2 | 100_Exosc1_66583_Fw_pair2 | caccGGAACTCGGGCTGTACTC | dual gRNAs for inducible knockout of Exosc1 |
|  | 101_Exosc1_66583_Rev_pair2 | aaacGAGTACAGCCCGAGTTCC |  |
|  | 102_Exosc1_66583_BbsI_Fw_pair2 | cttgtttGACTACTGCTGAAAACGAGCTgttta |  |
|  | 103_Exosc1_66583_BbsI_Rev_pair2 | ctcttaaacAGCTCGTTTTCAGCAGTAGTCaaa |  |
| Exosc1 pair3 | 104_Exosc1_66583_Fw_pair3 | caccGGGAGCTGTCGTCACCTGTA | dual gRNAs for inducible knockout of Exosc1 |
|  | 105_Exosc1_66583_Rev_pair3 | aaacTACAGGTGACGACAGCTCCC |  |
|  | 106_Exosc1_66583_BbsI_Fw_pair3 | cttgtttGAAAACGAGCTGGGCGTTGgttta |  |
|  | 107_Exosc1_66583_BbsI_Rev_pair3 | ctcttaaacCAACGCCCAGCTCGTTTTCaaa |  |
| Exosc1 pair4 | 108_Exosc1_66583_Fw_pair4 | caccGGCCAGGTGACATAGTTT | dual gRNAs for inducible knockout of Exosc1 |
|  | 109_Exosc1_66583_Rev_pair4 | aaacAAACTATGTCACCTGGCC |  |
|  | 110_Exosc1_66583_BbsI_Fw_pair4 | cttgtttGTCCCACATCTGGAAGCAACTgttta |  |
|  | 111_Exosc1_66583_BbsI_Rev_pair4 | ctcttaaacAGTTGCTTCCAGATGTGGGACaaa |  |
| Exosc2 | Exosc2_227715_1st_gRNA_Fw | caccGGTCGGAGACATTGTAGTG | dual gRNAs for inducible knockout of Exosc2 |
|  | Exosc2_227715_1st_gRNA_Rev | aaacCACTACAATGTCTCCGACC |  |
|  | Exosc2_227715_2nd_gRNA_Fw | cttgtttGTCATCCATGAACCTACCTGGgttta |  |
|  | Exosc2_227715_2nd_gRNA_Rev | ctcttaaacCCAGGTAGGTTCATGGATGACaaa |  |
| Exosc3 | Exosc3_66362_1st_gRNA_Fw | caccGATCACTATGCCAATCACG | dual gRNAs for inducible knockout of Exosc3 |
|  | Exosc3_66362_1st_gRNA_Rev | aaacCGTGATTGGCATAGTGATC |  |
|  | Exosc3_66362_2nd_gRNA_Fw | cttgtttGTCTTACCTGGCATTTGAAGgttta |  |
|  | Exosc3_66362_2nd_gRNA_Rev | ctcttaaacCTTCAAATGCCAGGTAAGACaaa |  |
| Exosc4 | Exosc4_109075_1st_gRNA_Fw | caccGCAGCTACGACAGACCTTCG | dual gRNAs for inducible knockout of Exosc4 |
|  | Exosc4_109075_1st_gRNA_Rev | aaacCGAAGGTCTGTCGTAGCTGC |  |
|  | Exosc4_109075_2nd_gRNA_Fw | cttgtttGGATGCTGGGATACCTATGCgttta |  |
|  | Exosc4_109075_2nd_gRNA_Rev | ctcttaaacGCATAGGTATCCCAGCATCCaaa |  |
| Exosc5 | Exosc5_27998_1st_gRNA_Fw | caccGGCCTCAGGATCACTTCA | dual gRNAs for inducible knockout of Exosc5 |
|  | Exosc5_27998_1st_gRNA_Rev | aaacTGAAGTGATCCTGAGGCC |  |
|  | Exosc5_27998_2nd_gRNA_Fw | cttgtttGCCTGGCTGGAGTCTATGGGCgttta |  |
|  | Exosc5_27998_2nd_gRNA_Rev | ctcttaaacGCCCATAGACTCCAGCCAGGCaaa |  |
| Exosc6 | Exosc6_72544_1st_gRNA_Fw | caccGCAAGGGCTCCGCCTACCTGG | dual gRNAs for inducible knockout of Exosc6 |
|  | Exosc6_72544_1st_gRNA_Rev | aaacCCAGGTAGGCGGAGCCCTTGC |  |
|  | Exosc6_72544_2nd_gRNA_Fw | cttgtttGACCGGAGAAGGGAGCGCGGgttta |  |
|  | Exosc6_72544_2nd_gRNA_Rev | ctcttaaacCCGCGCTCCCTTCTCCGGTCaaa |  |
| Exosc7 | Exosc7_66446_1st_gRNA_Fw | caccGCTGGAGAAACCGAATGA | dual gRNAs for inducible knockout of Exosc7 |
|  | Exosc7_66446_1st_gRNA_Rev | aaacTCATTCGGTTTCTCCAGC |  |
|  | Exosc7_66446_2nd_gRNA_Fw | cttgtttGCCACGGCCATCCACCCGAgttta |  |
|  | Exosc7_66446_2nd_gRNA_Rev | ctcttaaacTCGGGTGGATGGCCGTGGCaaa |  |
| Exosc8 | Exosc8_69639_1st_gRNA_Fw | caccGTGAAGCTGGGGAATACCA | dual gRNAs for inducible knockout of Exosc8 |
|  | Exosc8_69639_1st_gRNA_Rev | aaacTGGTATTCCCCAGCTTCAC |  |
|  | Exosc8_69639_2nd_gRNA_Fw | cttgtttGAGCCACAACTGTCAACATgttta |  |
|  | Exosc8_69639_2nd_gRNA_Rev | ctcttaaacATGTTGACAGTTGTGGCTCaaa |  |
| Exosc9 | Exosc9_50911_1st_gRNA_Fw | caccGTGAAGAACGAGTAATGGA | dual gRNAs for inducible knockout of Exosc9 |
|  | Exosc9_50911_1st_gRNA_Rev | aaacTCCATTACTCGTTCTTCAC |  |
|  | Exosc9_50911_2nd_gRNA_Fw | cttgtttGATAATGCTGCTTAAAGACCgttta |  |
|  | Exosc9_50911_2nd_gRNA_Rev | ctcttaaacGGTCTTTAAGCAGCATTATCaaa |  |
| Exosc10 | Exosc10_50912_1st_gRNA_Fw | caccGGGACGACACCAAGTGGCA | dual gRNAs for inducible knockout of Exosc10 |
|  | Exosc10_50912_1st_gRNA_Rev | aaacTGCCACTTGGTGTCGTCCC |  |
|  | Exosc10_50912_2nd_gRNA_Fw | cttgtttGAAGGTACCTCTAAGTCCAgttta |  |
|  | Exosc10_50912_2nd_gRNA_Rev | ctcttaaacTGGACTTAGAGGTACCTTCaaa |  |
| Dis3 | Dis3_72662_1st_gRNA_Fw | caccGCAGGGAAAATTATTTGG | dual gRNAs for inducible knockout of Dis3 |
|  | Dis3_72662_1st_gRNA_Rev | aaacCCAAATAATTTTCCCTGC |  |
|  | Dis3_72662_2nd_gRNA_Fw | cttgtttGCATAGAATGAAATAGAAAGTgttta |  |
|  | Dis3_72662_2nd_gRNA_Rev | ctcttaaacACTTTCTATTTCATTCTATGCaaa |  |
| Dis3L | Dis3l_213550_1st_gRNA_Fw | caccGATCCTAGCCAACCACTGGG | dual gRNAs for inducible knockout of Dis3L |
|  | Dis3l_213550_1st_gRNA_Rev | aaacCCCAGTGGTTGGCTAGGATC |  |
|  | Dis3l_213550_2nd_gRNA_Fw | cttgtttGCGGCCCAGGAACTACTGGAgttta |  |
|  | Dis3l_213550_2nd_gRNA_Rev | ctcttaaacTCCAGTAGTTCCTGGGCCGCaaa |  |
| Olfr10 | 76_Olfr10gRNA_Fw | CACCGGAAGAGCTACAGTCACA | dual gRNAs for inducible knockout of Olfr10 |
|  | 77_Olfr10gRNA_Rev | AAACTGTGACTGTAGCTCTTCC |  |
|  | 90_Olfr10_18307_9_BbsI_Fw | cttgtttGACCTTCAATATCAGTTTGGGgttta |  |
|  | 91_Olfr10_18307_9_BbsI_Rev | ctcttaaacCCCAAACTGATATTGAAGGTCaaa |  |
| eYFP / GFP | 74_eGFP2gRNA_Fw | CACCGAGCTGGACGGCGACGTAAA | For inducible knockout of eYFP or GFP |
|  | 75_eGFP2gRNA_Rev | AAACTTTACGTCGCCGTCCAGCTC |  |
| CD9 | CD9_8 gRNA_Fw | caccGTACATTCTGATTGGAGCCG | For inducible knockout of CD9 |
|  | CD9_8 gRNA_Rev | aaacCGGCTCCAATCAGAATGTAC |  |
| Rpa3 | N/A | caccGACCGGCCCGTGTGCTTCGT | For inducible knockout of Rpa3 |
|  | N/A | aaacACGAAGCACACGGGCCGGTC |  |
| Exosc1 | Exosc1_66583_1st_gRNA_Fw | caccGATGTAGCCATGCCGGGTGT | For monoclonal knockout of Exosc1 |
|  | Exosc1_66583_1st_gRNA_Rev | aaacACACCCGGCATGGCTACATC |  |
| Exosc2 | Exosc2_227715_1st_gRNA_Fw | caccGGTCGGAGACATTGTAGTG | For knockout rescue of Exosc2 |
|  | Exosc2_227715_1st_gRNA_Rev | aaacCACTACAATGTCTCCGACC |  |
| Exosc3 | N/A | caccGTAGATACCGGAAGCTGCGA | For endogenous tagging of Exosc3 |
|  | N/A | aaacTCGCAGCTTCCGGTATCTAC |  |
| Exosc6 | mExosc6_C-term_gRNA1_Fw | caccGGTTTTTTCCAATCAGGGTG | For endogenous tagging of Exosc6 |
|  | mExosc6_C-term_gRNA1_Rev | aaacCACCCTGATTGGAAAAAACC |  |
| Exosc7 | mExosc7_LastExon_gRNA2_Fw | caccgCAGTGGAGAGAAATCACCCC | For endogenous tagging of Exosc7 |
|  | mExosc7_LastExon_gRNA2_Rev | aaacGGGGTGATTTCTCTCCACTGc |  |
| Exosc8 | mExosc8_C-term_gRNA1_Fw | caccgCAAAGAAGTGAGCAAGCTAC | For endogenous tagging of Exosc8 |
|  | mExosc8_C-term_gRNA1_Rev | aaacGTAGCTTGCTCACTTCTTTGc |  |
| Exosc9 | mExosc9_C-term_gRNA1_Fw | caccgAACTGCTAACTAAGCTAAAG | For endogenous tagging of Exosc9 |
|  | mExosc9_C-term_gRNA1_Rev | aaacCTTTAGCTTAGTTAGCAGTTc |  |
