# Supplementary material for: Cellular assembly and functional resilience of the mammalian RNA exosome
Source: EMBO J. 2026 Jun 25;45(15):5423–56. doi: 10.1038/s44318-026-00824-x (PMC13434269; doi:10.1038/s44318-026-00824-x)
Supplement: Supplementary file 4 [file 44318_2026_824_MOESM2_ESM.docx]

**Table EV2: RT-qPCR primers and Northern blot probes**.

| **Target** | **Oligo name** | **Oligo sequence** | **Application** |
| --- | --- | --- | --- |
| proRpl23 | 51_PROMPT_Rpl23_qPCR_Fw | GCCCCTCTCTGGATAACAGG | RT-qPCR |
|  | 52_PROMPT_Rpl23_qPCR_Rev | GTGATGTTGAAATCGCCGCA | RT-qPCR |
| proPou5f1 | 45_PROMPT_Pou5f1_qPCR_Fw | CACAAACCAGTTGCTCGGATG | RT-qPCR |
|  | 46_PROMPT_Pou5f1_qPCR_Rev | AGACGGGTGGGTAAGCAAGA | RT-qPCR |
| proAnkhd1 | Ankhd1 PROMPT Fw | CTTGGTCCTGAGAACTGTCCG | RT-qPCR |
|  | Ankhd1 PROMPT Rev | CGCTCCGAGGTCACTCAACA | RT-qPCR |
| Fam120aos | Fam120 aos Fw | CAGGAGTGACCCATCCATCG | RT-qPCR |
|  | Fam120 aos Rev | CCATCAAGGCCCTAAGCGC | RT-qPCR |
| Gapdh | Gapdh_Fw | CGTCCCGTAGACAAAATGGT | RT-qPCR |
|  | Gapdh_Rev | TTGATGGCAACAATCTCCAC | RT-qPCR |
| N/A | ITS2_probe_Rev | TTGGCCCTGCGAGCAAACTCCC | Northern blot |
| N/A | pre-18S_Rev | CAGTCTCCCGTTTAATGATCCT | Northern blot |
| N/A | 7SB_probe_Rev | CGATTGATCGTCAACCGACGCTC | Northern blot |
| N/A | 7SK_RNA_probe_Rev | AAGACCGGTCCTCCTCTATC | Northern blot |
| N/A | 5.8S_rRNA_probe_Rev | CCGCAAGTGCGTTCGAAGTGTCG | Northern blot |
| N/A | 18S_rRNA_probe_Rev | GAATTTCACCTCTAGCGGCG | Northern blot |
| N/A | 28S_rRNA_probe_Rev | GTGTCGAGGGCTGACTTTCAA | Northern blot |
